# General Ir-Catalyzed N–H Insertions of Diazomalonates into Aliphatic and Aromatic Amines

**DOI:** 10.1021/acs.orglett.3c03929

**Published:** 2024-01-26

**Authors:** Zhuang Zhong, Céline Besnard, Jérôme Lacour

**Affiliations:** † Department of Organic Chemistry, University of Geneva, Quai Ernest Ansermet 30, CH-1211 Genève 4, Switzerland; ‡ Laboratory of Crystallography, University of Geneva, Quai Ernest Ansermet 24, CH-1211 Genève 4, Switzerland

## Abstract

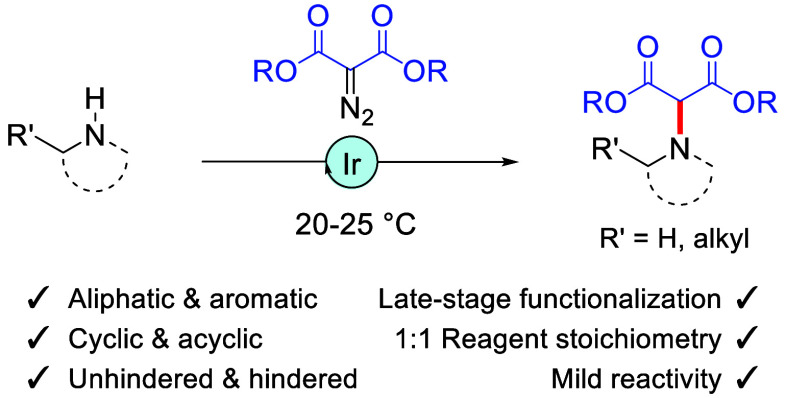

A general N–H insertion reactivity of acceptor–acceptor diazo malonate reagents is reported using [Ir(cod)Cl]_2_ as catalyst. A large range of amines, primary and secondary, aliphatic and aromatic, is possible. Mild temperatures, perfect substrate/reactant stoichiometry, and good functional group compatibility render the process particularly attractive for the (late-stage) functionalization of amines.

Nitrogen-containing molecules are key to pharmaceutical and medicinal chemistry since >80% of small molecule drugs contain at least one N atom,^1^ usually present as a heterocycle. Development of methods to access such (cyclic) compounds is thus of academic and industrial importance, and so are processes which allow the easy manipulation of the introduced N atoms.^2^ In fact, nitrogen is not a trivial atom to handle due to its basicity, nucleophilicity, high polarity, and coordination ability.^3^ For instance, processes as simple as the synthesis of trisubstituted amines from disubstituted alkyl or aryl precursors are often complex. To achieve such a goal,^4^ one prominent alternative is the insertion of reactive carbenes into pre-existing N–H bonds.^5^ Diazo derivatives are classical substrates to generate the necessary divalent intermediates, and their decomposition in the presence of transition-metal catalysts is a recognized strategy to form carbenes.^6^ Traditionally, different classes of diazo precursors are considered depending on the donor or acceptor nature of the substituents surrounding the central functional group.^7^ Donor–acceptor diazo reagents are used predominantly in N–H insertions, with much success including for asymmetric variants.^8^ However, if it becomes necessary to use acceptor–acceptor diazo reactants, one can rely on a few reported studies. In effect, little reactivity is known using diazomalonates **1**, despite the overall interest in these more stable diazo reagents,^9^ and in the resulting N-protected adducts.^10^ Livant and Moody reported, in the presence of Rh_2_(OAc)_4_, N–H insertions of **1** into bulky secondary alkylamines or anilines (Scheme 1).^11^ These specific substrates are hindered or less basic than regular amines—to avoid catalyst poisoning of the *Lewis* acidic dirhodium complexes.^11a,11d,12^ Also, previously, Sivasankar and co-workers reported the room temperature reactivity of diazomalonates **1** and anilines in water under iridium catalysis.^13^ The method caught our attention for its mildness,^14^ and we wondered how general the reaction could be in more classical solvent conditions and, importantly, with unhindered primary/secondary (heterocyclic) amines as substrates. Herein, we show that [Ir(cod)Cl]_2_ (cod = 1,5-cyclooctadiene), but also [CpRu(NCCH_3_)_3_][BAr_F_],^15^ are effective catalysts in nonpolar solvents (Scheme 1). The reactivities of both complexes are compared, and the commercial iridium dimer was overall preferred for its reactivity at room temperature and in the presence of a range of amines, including polyfunctional drugs such as Amoxapine,^16^ Vortioxetine,^17^ Pomalidomide,^18^ or sensitive unsaturated diaza macrocycles.

**Scheme 1:**
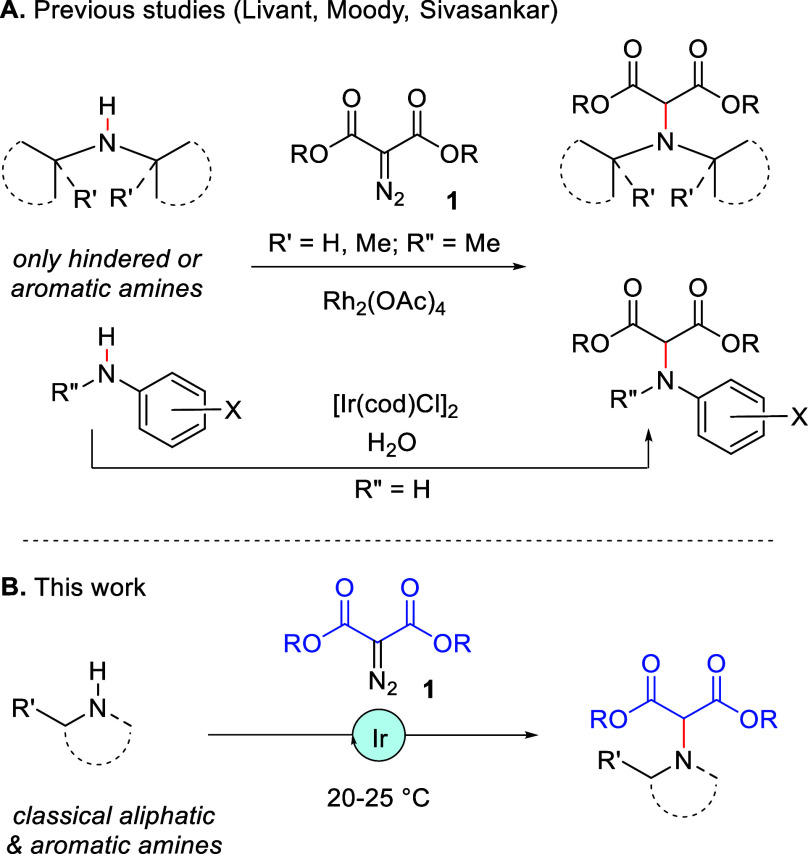
N–H Insertions of Diazomalonates **1**

With the goal of achieving NH insertions of diazomalonates into regular (unhindered) aliphatic amines, initial experiments were performed by adding dimethyl diazomalonate **1a** (0.5 mmol) to solutions of morpholine **2a** (1.0 equiv). Morpholine was chosen to test the (unlikely) competition between nitrogen and oxygen ylide pathways. 1,2-Dichloroethane was selected as solvent, and as a benchmark, the reaction was attempted first with classical Rh_2_(Oct)_4_ (1 mol %) as catalyst (Table 1, entry 1). After 15 h at 100 °C, full conversion of diazo **1a** was achieved but N–H insertion product **3aa** could not be observed, as expected from previous studies.^11a,11c,19^ This was also the case with Pd(OAc)_2_ and Pd(acac)_2_ as catalysts (entries 2 and 3).^20^ With copper salt Cu(OTf)_2_ and complex [Cu(CH_3_CN)_4_][BF_4_], adduct **3aa** was obtained in 15% and 11% NMR yields, respectively (entries 4 and 5). Then, iridium catalysts were tested. Both dimeric [Ir(cyclooctene)_2_ Cl]_2_ and [Ir(cod)Cl]_2_ gave excellent results under high temperature conditions using a strict 1:1 ratio between **1a** and **2a** (entries 6 and 7, NMR yields up to 96%), with a preference for the latter complex. [Ir(cod)Cl]_2_ was thus selected for further studies, and the reaction was performed at different temperatures (20–100 °C range) with effective conversions and yields (entries 7–9).

**Table 1:**
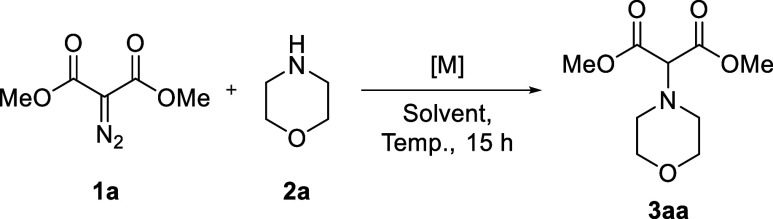
Optimization of Reaction Conditions^a^

entry	[M] (*x* mol %)	temp (°C)	yield (%)^b^
1	Rh_2_(Oct)_4_ (1)	100	ND^c^
2	Pd(OAc)_2_ (2)	100	ND^c^
3	Pd(acac)_2_ (2)	100	ND^c^
4	Cu(OTf)_2_ (2)	100	15
5	[Cu(NCCH_3_)_4_][BF_4_] (2)	100	11
6	[Ir(cyclooctene)_2_Cl]_2_ (1)	100	80
7	[Ir(cod)Cl]_2_ (1)	100	96
8	[Ir(cod)Cl]_2_ (1)	60	97
9	[Ir(cod)Cl]_2_ (1)	25	96
10^d^	[Ir(cod)Cl]_2_ (1)	25	96
11	[CpRu(NCCH_3_)_3_][BAr_F_] (2)	60	99
12^d^	[CpRu(NCCH_3_)_3_][BAr_F_] (2)	25	91

a Reaction conditions: **1a** (0.5 mmol), **2a** (0.5 mmol, 1.0 equiv), and [M] (1 or 2 mol %) in 1,2-dichloroethane (1.0 mL) for 15 h, unless otherwise noted.

b NMR yield (^1^H NMR spectroscopy using CH_2_Br_2_ as internal reference).

c ND = not detected.

d Reaction in DCM.

Then, several solvents were tested with general success (see Supporting Information); dichloromethane (DCM) was selected for its practicality (entry 10). In our group, cationic [CpRu(NCCH_3_)_3_]^+^ (Cp = cyclopentadienyl) complexes are known to be also effective catalysts for the decomposition of acceptor–acceptor diazo reagents under mild conditions.^15,21^ While an excellent result was obtained at 60 °C (99% NMR yield, entry 11), a slightly lower yield was observed at room temperature in DCM (91% NMR yield, entry 12). Considering the effectiveness of the reaction and the commercial availability of [Ir(cod)Cl]_2_, this complex was thus retained.^22^

With optimized conditions in hand (Table 1, entry 10), a variety of diazo reagents (**1a**–**h**) was tested (0.5 mmol) using morpholine **2a** as substrate, and full conversion was always reached after 15 h (Scheme 2). In the ester series from **3aa** to **3da**, from OMe to O^*t*^Bu, isolated yields were >80% (up to 91%) with a slight sensitivity to steric hindrance in the case of **3da** (81%). Excellent yields were obtained for benzylated **3ea** (93%) and **3ga** (89%). Clearly, under the Ir-catalysis and in the presence of morpholine, competitive Buchner reactions of **1e** and **1g**, in favor of products of intramolecular carbene addition onto an aromatic phenyl group, do not seem to happen.^23^ With **1f** and **1h** carrying 2,2,2-trifluoroethyl side chains on the ester groups, yields were slightly lower, 78% and 79% for **3fa** and **3ha** respectively. However, with cyclic **1i**, a complete lack of decomposition was noticed; this reagent often presented an orthogonal reactivity in comparison with acyclic derivatives.^24^

**Scheme 2:**
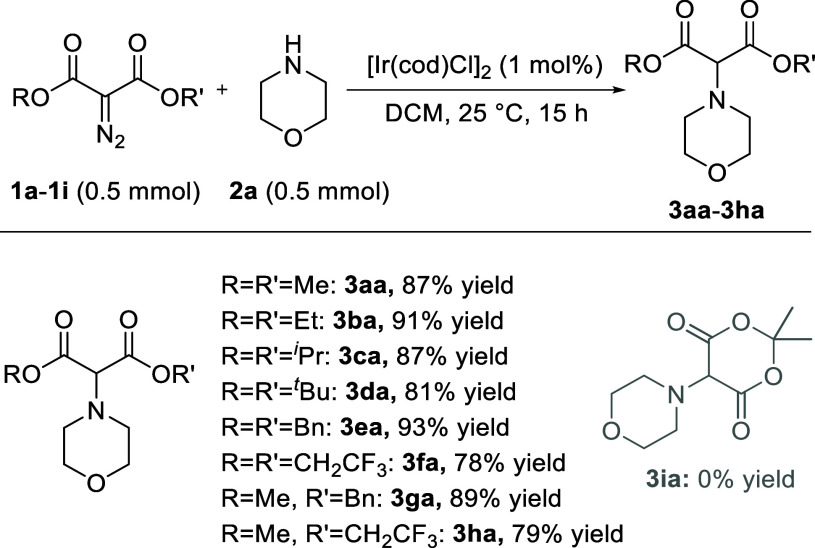
Diazomalonate Reactivity

The reaction was then extended to a variety of cyclic, acyclic, and aromatic secondary amines to afford tertiary products **3ab** to **3am** in yields up to 93% (Scheme 3). Overall, the reaction is general, and few exceptions will be noted (*vide infra*). Sometimes, an increase in reaction time and temperature was needed for full conversion but a strict 1:1 stoichiometry between diazo reagent and amine moieties could be maintained. Satisfactorily, different ring sizes were amenable, from 4- to 7-membered rings (**3ab**–**3ae**, 52–79%). While azetidine **2b** reacted to yield volatile **3ab** in a subsequently lower yield (52%, NMR), reaction with 2-methylaziridine did not form the expected three-membered azeridine but the 2-(allylamino)malonate adduct instead. With tetrahydroisoquinoline **2f**, product **3af** was obtained in 77% yield. 4-Oxo-piperidine **2g** reacted to afford **3ag** in 59% yield; the presence of the ketone moiety perturbs possibly the transformation via competitive formation of a carbonyl ylide intermediate. With piperazine **2h**, a double N–H insertion was possible using 2 equiv of diazo. Compound **3ah** was prepared in an excellent 87% yield using twice the regular amount of **1** for the 2-fold process. The structure of **3ah** was confirmed unambiguously by X-ray analysis (Scheme 3). Acyclic secondary amines **2i** and **2j** were tested. While the reaction proceeded well to form **3ai** in 81% yield, the reactivity of hindered diisopropylamine was poor. It required a higher reaction temperature (60 °C) and adduct **3aj** was isolated with only a 12% yield. This led us to test the effect of steric hindrance in the cyclic series with 2-methyl pyrrolidine **2k** and piperidine **2l** as substrates. The corresponding products were afforded in moderate yields, **3ak** (59%) and **3al** (55%); the reaction also required a slightly elevated temperature.

**Scheme 3:**
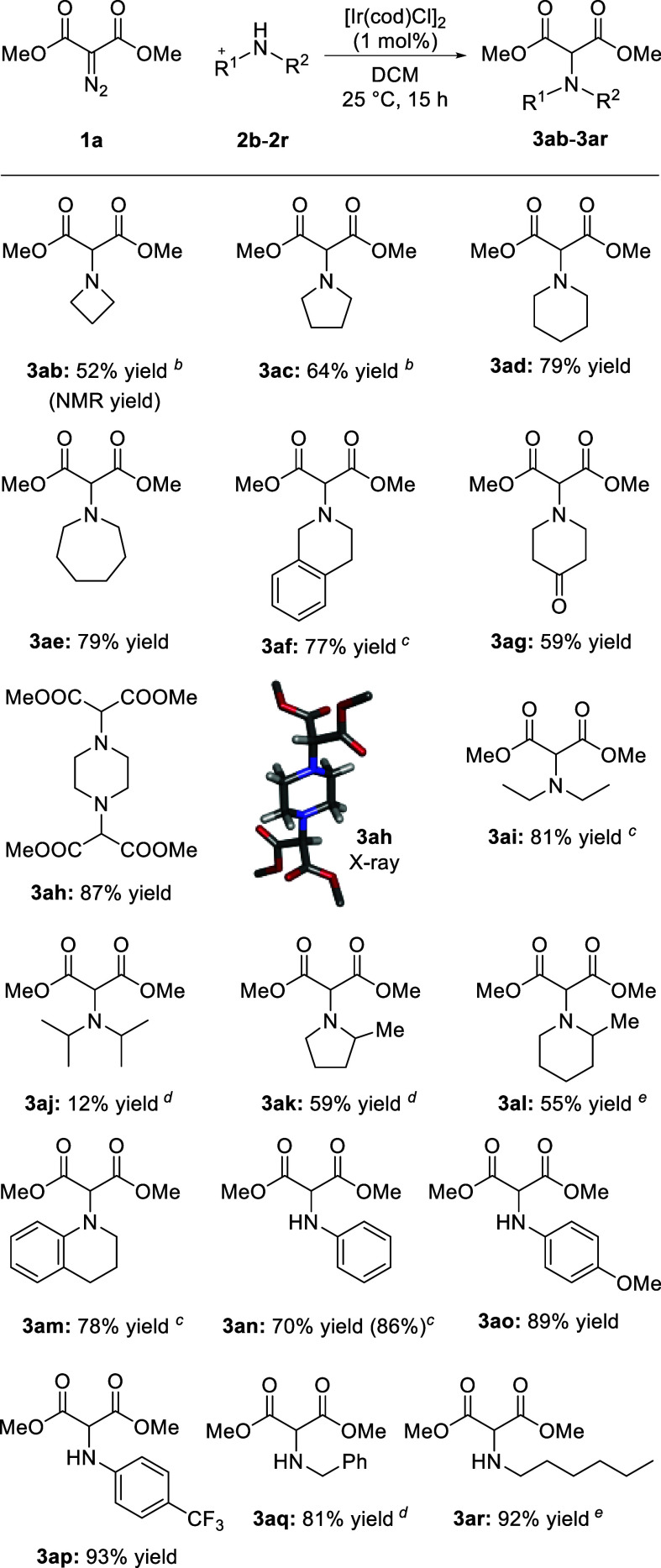
Amine Reactivity General conditions: **1a** (0.5 mmol), **2b**–**2r** (0.5 mmol, 1.0 equiv), and [Ir(cod)Cl]_2_ (1 mol %) in DCM (1.0 mL) at 25 °C for 15 h. Isolated yields to the exception of **3ab** (NMR). 48 h. 72 h. 60 °C for 36 h. 60 °C for 15 h.

Then, sp^2^-hybridized nitrogen atoms were considered using secondary tetrahydroquinoline **2m** first. Not surprisingly,^13^ the reaction proceeded well to yield **3am** (78%). Next, primary anilines were tested, and good yields were obtained irrespective of electron-neutral, -rich, or -poor character of the N atom (**3an**–**3ap**, 86–93%). Good yields could be achieved with aniline, 70% or 86%, after 15 or 72 h of reaction, respectively. Acyclic aliphatic amines **1q** and **1r** were also used as substrates. Overall, to the exception of a few polyamino or bulky substrates (see Scheme S1), excellent yields were observed for the preparation of mono N–H insertion adducts regardless of the aromatic or aliphatic nature of N-substituents **3an**–**3ar** (81–92%). Finally, gram-scale experiments were performed using dimethyl diazomalonate **1a** and morpholine **2a** in DCM. After successful attempts under regular conditions, it was found that the catalyst loading could be reduced even to 0.5 mol %, without any impact on the yield (91%) but with an elongated reaction time of 72 h. A mechanistic rationale is proposed in Scheme S2.

With a larger amount of dimethyl 2-morpholinomalonate **3aa** in hand, a series of subsequent reactions was attempted (Scheme 4). Deprotonation of the malonate moiety could be readily achieved with ^*t*^BuOK and, in the presence of formaldehyde, Knoevenagel adduct **4** was isolated in 47% yield. Alkylations with methyl iodide and propargyl bromide could be realized using NaH as a base to afford **5** (58%) and **6** (77%). Decarboxylative procedure (LiCl, 150 °C) afforded ester **7** (77% NMR yield). Unfortunately, the product was quite volatile and its isolation from DMF low yielding. Of note, this process opens the door to the formation of aminocarboxylate side chains that are useful in many metal binding studies.^25^ Reaction of **3aa** with *n*-hexylamine, used as a solvent, led to bisamide **8** (75%).

**Scheme 4:**
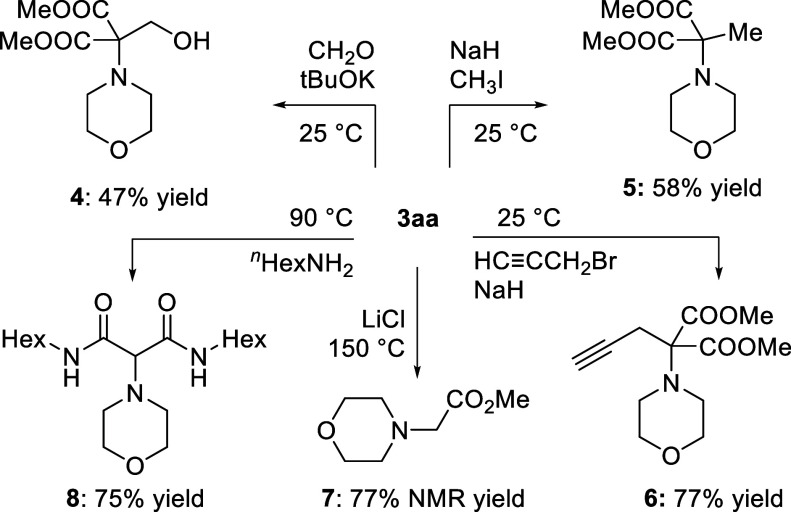
Post-transformations

Finally, to demonstrate the utility and mildness of the NH-insertion method, it was studied in the context of late-stage functionalizations with molecules from medical chemistry or our own laboratory (Scheme 5). With Amoxapine and Vortioxetine, molecules used in the treatment of (major) depression, N-protected adducts **9** and **10** were obtained in excellent 99% and 98% yields, respectively. Importantly, side reactions involving ylide formation by the addition of carbene intermediates to the Lewis basic N(sp^2^) of Amoxapine or the S atom of Vortioxetine did not occur. With Pomalidomide, an anticancer drug, **11** was obtained in 81% by insertion into the primary aniline exclusively; evidence of reactivity on the more acidic imide NH fragment could not be found.

**Scheme 5:**
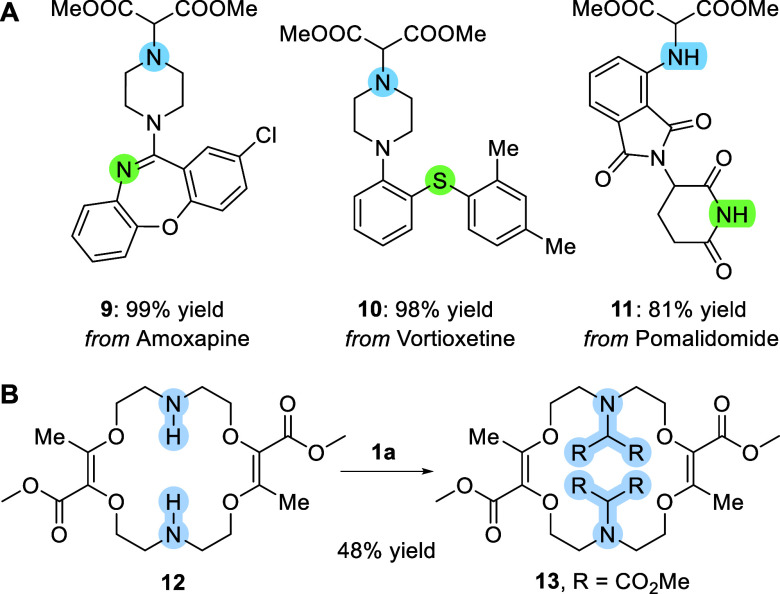
Late-Stage Reactivity **A**: amines (0.25 mmol), **1a** (0.25 mmol, 1.0 equiv), and [Ir(cod)Cl]_2_ (1 mol %) in DCM (0.5 mL) at 25 °C for 20 h (with Amoxapine and Vortioxetine) or at 60 °C for 72 h (with Pomalidomide). **B**: **12** (0.1 mmol), **1a** (0.3 mmol, 3.0 equiv), and [Ir(cod)Cl]_2_ (2 mol %) in DCM (0.6 mL) at 60 °C for 72 h.

Previously, in our own group, an original [3 + 6 + 3 + 6] macrocyclization procedure was discovered using α-diazo-β-ketoesters and cyclic ethers as substrates.^26^ This reaction was extended to the formation of diaza macrocycles with morpholine heterocycles as the starting materials. For instance, using this protocol, bis-NH derivative **12** can be isolated in 68% yield after two steps only.^26b^ In our hand, this derivative was found to be sensitive to both acidic and basic conditions, hence limiting the possibility of functionalization of the N atoms and consequently the introduction of acetic acid side chains. Care was thus taken to investigate the NH-insertion reactivity. Satisfactorily, bis-functionalized **13** was obtained in 48% yield, after an increase in catalyst loading (2 mol %) and diazo reagent (**1a**, 3.0 equiv), and conditions at 60 °C for 72 h. This unusual difficulty in forming the insertion adducts might be related to conformations of **12** that present, most likely, the two N–H bonds inward, toward the lumen of the macrocycle, thus strongly hindering the accessibility of the heteroatoms.

In conclusion, the overall reactivity demonstrates, including these latest examples, the generality of Ir-catalyzed N–H insertion even in the presence of steric encumbrance. The functional group compatibility, together with the mild reaction temperatures, and usually perfect substrate/reactant stoichiometry render the process particularly attractive for the (late-stage) functionalization of amines.

## Data Availability

The data underlying this study are openly available at yareta.unige.ch under DOI: 10.26037/yareta:blwwovyd75g5vox7g7i42zvnhm. It will be preserved for 10 years.

## Supporting Information Available

Synthetic protocols and spectroscopic characterizations; ^1^H NMR and ^13^C NMR spectra of new compounds (PDF)
