# Supplementary material for: General Ir-Catalyzed N–H Insertions of Diazomalonates into Aliphatic and Aromatic Amines
Source: Org Lett. 2024 Jan 26;26(5):983–7. doi: 10.1021/acs.orglett.3c03929 (PMC10863398; doi:10.1021/acs.orglett.3c03929)
Supplement: Supplementary file 1 — ol3c03929_si_001.pdf [file ol3c03929_si_001.pdf]

Supporting information

General Ir-Catalyzed N-H Insertions of Diazomalonates into Aliphatic and Aromatic Amines

Zhuang Zhong,[a] Céline Besnard,[b] Jérôme Lacour *[a]

a Department of Organic Chemistry, University of Geneva
Quai Ernest Ansermet 30, 1211 Geneva 4, Switzerland.
E-mail: jerome.lacour@unige.ch

b Laboratoire de Cristallographie, University of Geneva
Quai Ernest Ansermet 24, 1211 Geneva 4, Switzerland.

Table of Contents

1. General information S1
2. Experimental results S3
3. Mechanistic rationale S6
4. Synthesis and data analysis of N-H insertion adducts S7
5. Post-transformation S21
6. Late-stage reactivity S25
7. Synthesis and data analysis of aza-macrocycle S28
8. X-ray information S29
9. NMR spectra S31
10. References S73

1. General information

1.1 Reagents and solvents

Unless otherwise stated, reagents and (dry) solvents were purchased from commercial sources and used without further purification. Additionally, dry tetrahydrofuran (THF) was distilled with sodium. All reactions involving air sensitive compounds were carried out under N_2 via an inert gas/vacuum double manifold line and standard Schlenk techniques using dry solvents. Reactions involving oxygen sensitive reagents were performed using degassed solvents.

Important note: *Diazo compounds are high energy materials and should be handled with caution. Although α-diazomalonates are stable at room temperature, it is advisable to carry out reactions behind a blast shield.*

All reagents **1** were prepared according to literature specifications: **1a**,[1] **1b**,[1] **1c**,[1] **1d**,[1] **1e**,[1] **1f**,[1] **1g**,[2] **1h**,[3] **1i**.[4] Corresponding ^{1}H NMR spectra are provided in section 9.

1.2 Thin layer chromatography and purification

Analytical thin layer chromatography (TLC) was performed with Silica gel 60 F_{254} aluminum plates purchased from Merck. Column chromatography were performed using Silica flash P60 silica gel (40-63 µm, 60 Å).

1.3 Nuclear magnetic resonance (NMR)

NMR spectra were recorded on Bruker Avance III 500 MHz, Bruker Avance III HD-*NanoBay* 400 MHz and Bruker Avance III HD-*NanoBay* 300 MHz spectrometers at room temperature. NMR chemical shifts are given in ppm (δ) relative to Me_4Si with solvent resonances used as internal standards (**$CDCl_3$**: 7.27 ppm for ^{1}H and 77.16 for ^{13}C). Proton (^{1}H) NMR information is given in the following format: multiplicity (s, singlet; d, doublet; t, triplet; q, quartet; quin, quintet; sex, sextet; sept, septet; m, multiplet), the prefix br- was applied when the signal was broadened, coupling constant(s) (*J*) in Hertz (Hz), number of protons.

1.4 Infrared Spectroscopy

IR spectra were recorded on a Perkin-Elmer 1650 FT-IR spectrometer using a diamond ATR Golden Gate sampling.

1.5 High-resolution Mass Spectrometry (HRMS)

Electrospray mass spectra were obtained on a Xevo-G2-TOF HRMS by the Department of Mass Spectroscopy of the University of Geneva.

2. Experimental results

2.1 Optimization of reaction conditions

Table S1 The screening of metal catalysts [a]

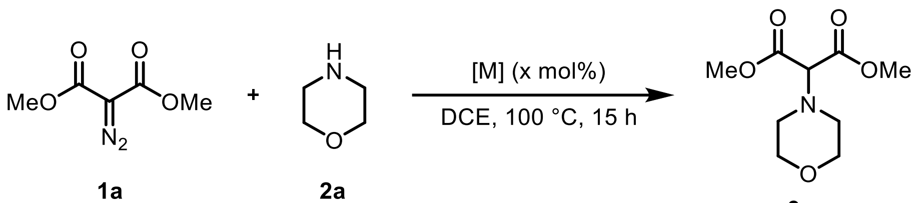

Entry	[M]	x mol%	Yield (%) [b]
1	$Rh_2(OAc)_4$	1	ND [d]
2	$Rh_2(Oct)_4$	1	ND [d]
3	$Pd(OAc)_2$	2	ND [d]
4	$PdCl_2$	2	ND [d]
5	$Pd(acac)_2$	2	ND [d]
6	$Cu(OTf)_2$	2	15
7	$[Cu(CH_3CN)_4][BF_4]$	2	11
**8**	**$[Ir(cod)Cl]_2$**	**1**	**96**
9	$[Ir(cyclooctene)_2Cl]_2$	1	80
10 [c]	$[CpRu(CH_3CN)_3][BAr_F]$	2	99
11 [c]	$[Cp^*Ru(CH_3CN)_3][PF_6]$	2	89
12 [c]	$[Cp^*Ru(CH_3CN)_3][SbF_6]$	2	92
13 [c]	$[(Cp^*\text{-}CF_3)Ru(CH_3CN)_3][SbF_6]$	2	95

[a] General reaction conditions: diazo **1a** (0.5 mmol), morpholine **2a** (1.0 equiv) and [M] (1-2 mol%) in DCE (1.0 mL) at 100 ºC for 15 h. [b] NMR yields (^1H NMR spectroscopy using CH_2Br_2 as reference). [c] At 60 ºC for 4 h. [d] ND = not desired product.

Table S2 Optimization of temperature and solvents [a]

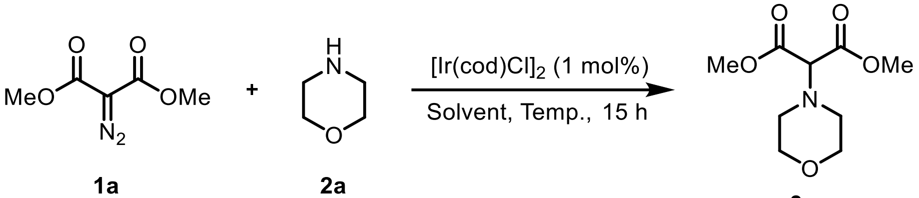

1a **2a** **3aa**

Entry	Temp. (ºC)	Solvent	Yield (%) [b]
1	100	DCE	96
2	60	DCE	97
3	r.t. [d]	DCE	96
4	r.t. [d]	$CHCl_3$	96
**5**	**r.t.** [d]	**DCM**	**96**
6	r.t. [d]	THF	86
7	r.t. [d]	*n*-Hexane	97
8	r.t. [d]	Toluene	97
9	r.t. [d]	H_2O	8
10 [c]	r.t. [d]	DCM	91

[a] General reaction conditions: diazo **1a** (0.5 mmol), morpholine **2a** (1.0 equiv) and $[Ir(cod)Cl]_2$ (1 mol%) in solvent (1.0 mL) for 15 h. [b] NMR yields (^{1}H NMR spectroscopy using CH_2Br_2 as reference). [c] $[CpRu(CH_3CN)_3][BAr_F]$ (2 mol%) was used instead of $[Ir(cod)Cl]_2$. [d] 20-25 ºC range.

2.2 The result of reaction with 2-methylaziridine

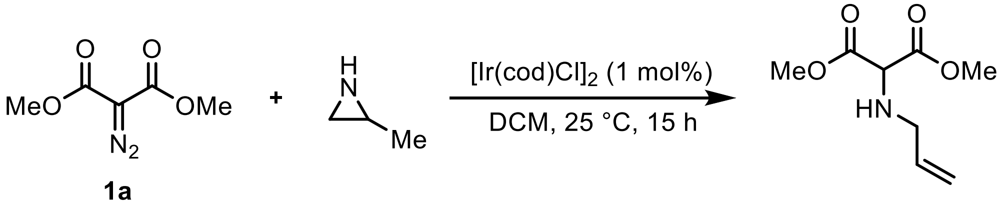

1a

(S1)

2.3 Limitations

In the study of this reactivity, some attempts did not yield the targeted results. For instance, using cyclic diazo **1i**, under the best condition of iridium catalysis, decomposition of the diazo reagent did not occur and, consequently, N-H insertion adduct **3ia** was not obtained. Also, care was taken to test the reactivity of other amines (Scheme S1). At either 25 or 60 °C with $[Ir(cod)Cl]_2$ as catalyst, the corresponding adducts were not obtained from diazo reagent **1a** and cyclic polyamines **2s**. With hindered 2,6-dimethylpiperidine **2t**, the desired N-H insertion adduct was not formed, again indicating a sensitivity to steric hindrance. Furthermore, the insertion into drug molecules, Levetiracetam **2u** (epilepsy) and Bupropion **2v** (antidepressant), could not be achieved.

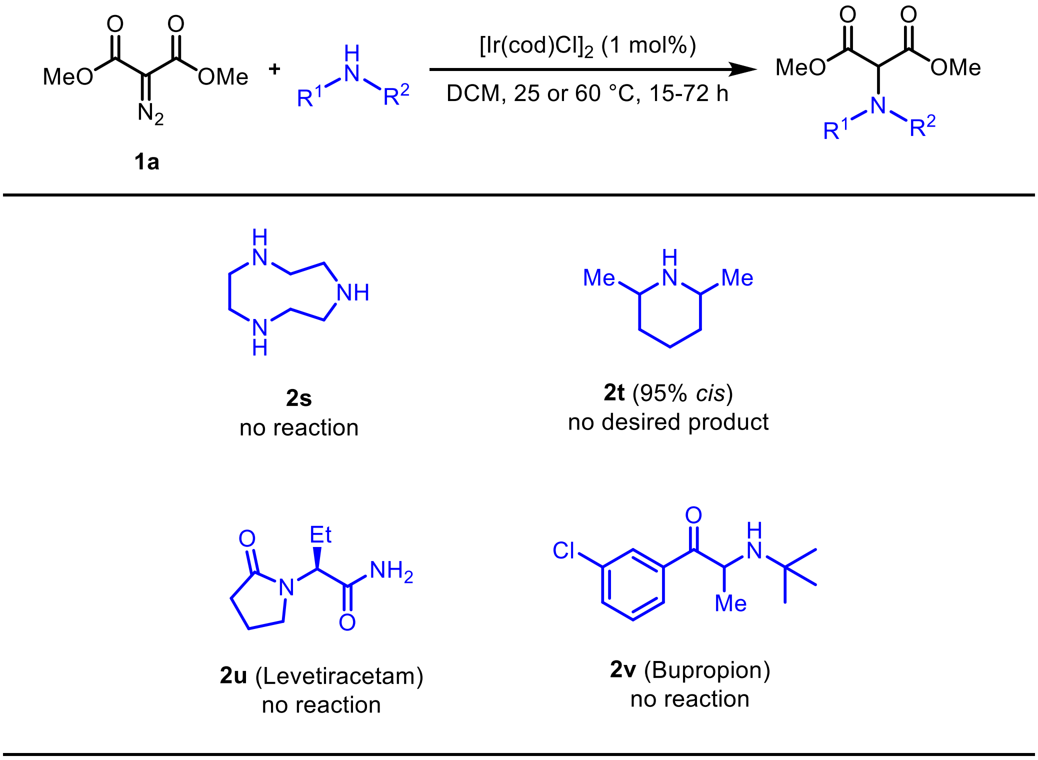

Scheme S1 Limitations of amines

3. Mechanistic rationale

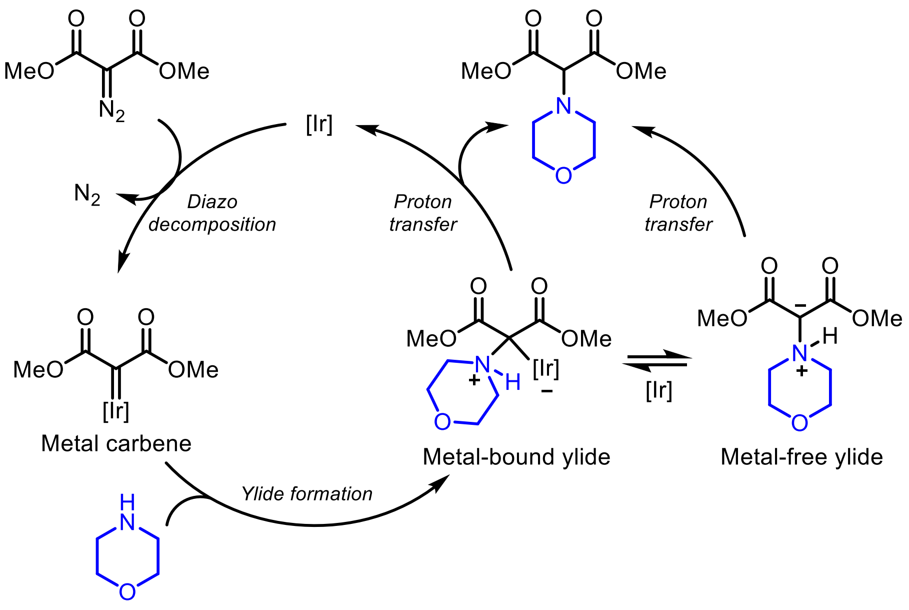

Scheme S2 Proposed mechanism

4. Synthesis and data analysis of N-H insertion adducts

4.1 General procedure A

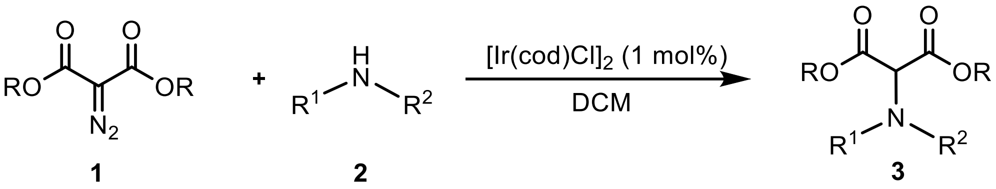

In a 2 mL screw-cap vial equipped with a magnetic stirring bar, the diazo compound **1** (0.5 mmol, 1.0 equiv), amines **2** (0.5 mmol, 1.0 equiv) and $[Ir(cod)Cl]_2$ (1 mol%) were dissolved in DCM (1.0 mL). The vial was capped and the solution was stirred at 25 or 60 °C for 15-72 h. The solution was concentrated in vacuum and the residue was purified by column chromatography to afford the desired product.

4.2 Data analysis

Dimethyl 2-morpholinomalonate (3aa) [5]

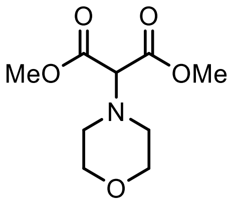

Following the general procedure A with dimethyl 2-diazomalonate **1a** (79 mg, 0.5 mmol, 1.0 equiv), morpholine **2a** (44 mg, 0.5 mmol, 1.0 equiv), $[Ir(cod)Cl]_2$ (3.3 mg, 0.005 mmol, 1 mol%) and DCM (1.0 mL), the reaction was performed at 25 °C for 15 h. The mixture was purified by column chromatography to afford the desired product **3aa** as white solid (94 mg, 87% yield). R_f = 0.3 (pentane/ethyl acetate = 1:1 v/v). M.p.: 55-56 °C.

^{1}H NMR (500 MHz, $CDCl_3$) δ 4.05 (s, 1H), 3.78 (s, 6H), 3.77 – 3.73 (m, 4H), 2.78 – 2.74 (m, 4H). **^{13}C NMR** (126 MHz, $CDCl_3$) δ 167.2, 70.8, 67.1, 52.4, 50.5. **IR** (neat) 2961, 2848, 1730, 1442, 1388, 1354, 1337, 1302, 1267, 1231, 1154, 1108, 1073, 1037, 1025, 1014, 973, 953, 937, 918, 866, 803, 776, 705 cm^{-1}. **HRMS (ESI)** Calculated for $C_9H_{16}NO_5$ $[M+H]^+$: 218.1023; Found: 218.1034 m/z.

Diethyl 2-morpholinomalonate (3ba)

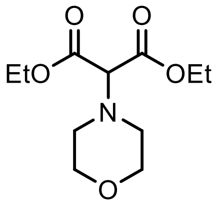

Following the general procedure A with diethyl 2-diazomalonate **1b** (93 mg, 0.5 mmol, 1.0 equiv), morpholine **2a** (44 mg, 0.5 mmol, 1.0 equiv), $[Ir(cod)Cl]_2$ (3.3 mg, 0.005 mmol, 1 mol%) and DCM (1.0 mL), the reaction was performed at 25 °C for 15 h. The mixture was purified by column chromatography to afford the desired product **3ba** as colorless oil (112 mg, 91% yield). R_f = 0.5 (pentane/ethyl acetate = 1:1 v/v).

^{1}H NMR (500 MHz, $CDCl_3$) δ 4.26 (q, J = 7.1 Hz, 4H), 4.01 (s, 1H), 3.80 – 3.73 (m, 4H), 2.83 – 2.75 (m, 4H), 1.30 (t, J = 7.1 Hz, 6H). **^{13}C NMR** (126 MHz, $CDCl_3$) δ 166.8, 71.0, 67.1, 61.5, 50.6, 14.2. **IR** (neat) 2974, 2856, 1729, 1450, 1369, 1349, 1297, 1263, 1223, 1150, 1114, 1071, 1027, 945, 913, 879, 857, 767 cm^{-1}. **HRMS (ESI)** Calculated for $C_{11}H_{20}NO_5$ $[M+H]^+$: 246.1337; Found: 246.1354 m/z.

Diisopropyl 2-morpholinomalonate (3ca)

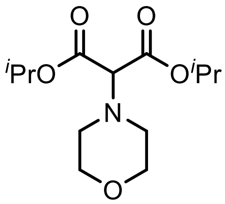

Following the general procedure A with diisopropyl 2-diazomalonate **1c** (107 mg, 0.5 mmol, 1.0 equiv), morpholine **2a** (44 mg, 0.5 mmol, 1.0 equiv), $[Ir(cod)Cl]_2$ (3.3 mg, 0.005 mmol, 1 mol%) and DCM (1.0 mL), the reaction was performed at 25 °C for 15 h. The mixture was purified by column chromatography to afford the desired product **3ca** as colorless oil (119 mg, 87% yield). R_f = 0.5 (pentane/ethyl acetate = 2:1 v/v).

^{1}H NMR (500 MHz, $CDCl_3$) δ 5.12 (hept, J = 6.3 Hz, 2H), 3.94 (s, 1H), 3.79 – 3.73 (m, 4H), 2.83 – 2.76 (m, 4H), 1.28 (d, J = 6.3 Hz, 6H), 1.27 (d, J = 6.3 Hz, 6H). **^{13}C NMR** (126 MHz, $CDCl_3$) δ 166.4, 71.2, 69.3, 67.2, 50.6, 21.8, 21.7. **IR** (neat) 2981, 2856, 1726, 1453, 1375, 1293, 1265, 1228, 1161, 1100, 1022, 989, 961, 903, 869, 837, 801 cm^{-1}. **HRMS (ESI)** Calculated for $C_{13}H_{24}NO_5$ $[M+H]^+$: 274.1649; Found: 274.1654 m/z.

Di-*tert*-butyl 2-morpholinomalonate (3da)

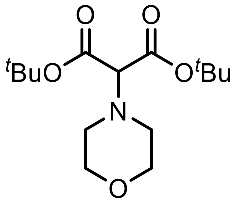

Following the general procedure A with di-*tert*-butyl 2-diazomalonate **1d** (121 mg, 0.5 mmol, 1.0 equiv), morpholine **2a** (44 mg, 0.5 mmol, 1.0 equiv), $[Ir(cod)Cl]_2$ (3.3 mg, 0.005 mmol, 1 mol%) and DCM (1.0 mL), the reaction was performed at 25 °C for 15 h. The mixture was purified by column chromatography to afford the desired product **3da** as white solid (122 mg, 81% yield). R_f = 0.2 (pentane/ethyl acetate = 10:1 v/v). M.p.: 44-45 °C.

^{1}H NMR (500 MHz, $CDCl_3$) δ 3.81 (s, 1H), 3.79 – 3.74 (m, 4H), 2.81 – 2.76 (m, 4H), 1.50 (s, 18H). **^{13}C NMR** (126 MHz, $CDCl_3$) δ 166.1, 82.3, 72.2, 67.3, 50.7, 28.1. **IR** (neat) 2977, 2953, 2849, 1750, 1718, 1450, 1391, 1368, 1316, 1272, 1240, 1203, 1139, 1113, 1071, 1036, 1020, 988, 954, 915, 876, 850, 828, 797, 740 cm^{-1}. **HRMS (ESI)** Calculated for $C_{15}H_{28}NO_5$ $[M+H]^+$: 302.1962; Found: 302.1970 m/z.

Dibenzyl 2-morpholinomalonate (3ea)

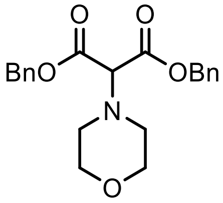

Following the general procedure A with dibenzyl 2-diazomalonate **1e** (155 mg, 0.5 mmol, 1.0 equiv), morpholine **2a** (44 mg, 0.5 mmol, 1.0 equiv), $[Ir(cod)Cl]_2$ (3.3 mg, 0.005 mmol, 1 mol%) and DCM (1.0 mL), the reaction was performed at 25 °C for 15 h. The mixture was purified by column chromatography to afford the desired product **3ea** as colorless oil (172 mg, 93% yield). R_f = 0.7 (pentane/ethyl acetate = 1:1 v/v).

^{1}H NMR (500 MHz, $CDCl_3$) δ 7.41 – 7.28 (m, 10H), 5.19 (d, *J* = 1.7 Hz, 4H), 4.13 (s, 1H), 3.75 – 3.71 (m, 4H), 2.80 – 2.76 (m, 4H). **^{13}C NMR** (126 MHz, $CDCl_3$) δ 166.6, 135.1, 128.6, 128.5, 128.4, 70.9, 67.2, 67.1, 50.5. **IR** (neat) 3035, 2959, 2855, 1730, 1498, 1454, 1379, 1294, 1263, 1215,

1144, 1114, 1071, 1023, 1002, 908, 863, 738, 696 cm^{-1}. **HRMS (ESI)** Calculated for $C_{21}H_{24}NO_5$ $[M+H]^+$: 370.1649; Found: 370.1661 m/z.

Bis(2,2,2-trifluoroethyl) 2-morpholinomalonate (3fa)

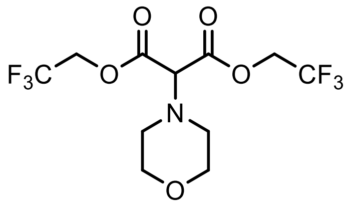

Following the general procedure A with bis(2,2,2-trifluoroethyl) 2-diazomalonate **1f** (147 mg, 0.5 mmol, 1.0 equiv), morpholine **2a** (44 mg, 0.5 mmol, 1.0 equiv), $[Ir(cod)Cl]_2$ (3.3 mg, 0.005 mmol, 1 mol%) and DCM (1.0 mL), the reaction was performed at 25 °C for 15 h. The mixture was purified by column chromatography to afford the desired product **3fa** as colorless oil (138 mg, 78% yield). R_f = 0.6 (pentane/ethyl acetate = 2:1 v/v).

^{1}H NMR (500 MHz, $CDCl_3$) δ 4.67 – 4.49 (m, 4H), 4.28 (s, 1H), 3.82 – 3.68 (m, 4H), 2.84 – 2.75 (m, 4H). **^{13}C NMR** (126 MHz, $CDCl_3$) δ 164.6, 122.5 (q, *J* = 277.2 Hz), 69.8, 67.0, 60.9 (q, *J* = 37.3 Hz), 50.3. **^{19}F NMR** (282 MHz, $CDCl_3$) δ -73.7. **IR** (neat) 2972, 2862, 1754, 1453, 1413, 1278, 1153, 1115, 1052, 1009, 978, 865, 770 cm^{-1}. **HRMS (ESI)** Calculated for $C_{11}H_{14}F_6NO_5$ $[M+H]^+$: 354.0771; Found: 354.0755 m/z.

1-Benzyl 3-methyl 2-morpholinomalonate (3ga)

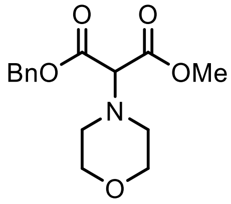

Following the general procedure A with 1-benzyl 3-methyl 2-diazomalonate **1g** (117 mg, 0.5 mmol, 1.0 equiv), morpholine **2a** (44 mg, 0.5 mmol, 1.0 equiv), $[Ir(cod)Cl]_2$ (3.3 mg, 0.005 mmol, 1 mol%) and DCM (1.0 mL), the reaction was performed at 25 °C for 15 h. The mixture was purified by column chromatography to afford the desired product **3ga** as colorless oil (130 mg, 89% yield). R_f = 0.4 (pentane/ethyl acetate = 1:1 v/v).

^{1}H NMR (500 MHz, $CDCl_3$) δ 7.40 – 7.33 (m, 5H), 5.23 (d, *J* = 2.2 Hz, 2H), 4.09 (s, 1H), 3.77 – 3.73 (m, 7H), 2.77 (td, *J* = 4.2, 2.5 Hz, 4H). **^{13}C NMR** (126 MHz, $CDCl_3$) δ 167.2, 166.7, 135.2,

128.6, 128.5, 128.4, 70.9, 67.2, 67.1, 52.3, 50.6. **IR** (neat) 2956, 2856, 1731, 1499, 1453, 1381, 1349, 1295, 1263, 1220, 1146, 1114, 1071, 1025, 905, 864, 748, 698 cm^{-1}. **HRMS (ESI)** Calculated for $C_{15}H_{20}NO_5$ $[M+H]^+$: 294.1337; Found: 294.1334 m/z.

1-Methyl 3-(2,2,2-trifluoroethyl) 2-morpholinomalonate (3ha)

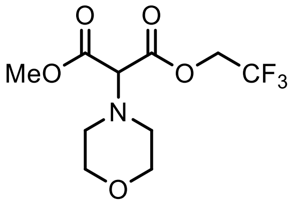

Following the general procedure A with 1-methyl 3-(2,2,2-trifluoroethyl) 2-diazomalonate **1h** (113 mg, 0.5 mmol, 1.0 equiv), morpholine **2a** (44 mg, 0.5 mmol, 1.0 equiv), $[Ir(cod)Cl]_2$ (3.3 mg, 0.005 mmol, 1 mol%) and DCM (1.0 mL), the reaction was performed at 25 °C for 15 h. The mixture was purified by column chromatography to afford the desired product **3ha** as colorless oil (127 mg, 79% yield). R_f = 0.7 (pentane/ethyl acetate = 2:1 v/v).

^{1}H NMR (500 MHz, $CDCl_3$) δ 4.65 – 4.51 (m, 2H), 4.17 (s, 1H), 3.80 (s, 3H), 3.76 (d, *J* = 4.7 Hz, 4H), 2.85 – 2.72 (m, 4H). **^{13}C NMR** (126 MHz, $CDCl_3$) δ 166.4, 165.4, 122.6 (q, *J* = 277.4 Hz), 70.3, 67.1, 60.6 (q, *J* = 37.0 Hz), 52.6, 50.4. **^{19}F NMR** (282 MHz, $CDCl_3$) δ -73.6. **IR** (neat) 2964, 2859, 1739, 1439, 1413, 1382, 1352, 1278, 1220, 1140, 1114, 1049, 1022, 977, 905, 865, 771 cm^{-1}. **HRMS (ESI)** Calculated for $C_{10}H_{15}F_3NO_5$ $[M+H]^+$: 286.0897; Found: 286.0890 m/z.

Dimethyl 2-(azetidin-1-yl)malonate (3ab)

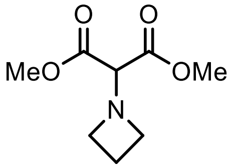

Following the general procedure A with dimethyl 2-diazomalonate **1a** (79 mg, 0.5 mmol, 1.0 equiv), azetidine **2b** (29 mg, 0.5 mmol, 1.0 equiv), $[Ir(cod)Cl]_2$ (3.3 mg, 0.005 mmol, 1 mol%) and DCM (1.0 mL), the reaction was performed at 25 °C for 48 h. The mixture (52% NMR yield) was purified by column chromatography to afford the desired product **3ab** as yellow oil (25 mg, 27% yield). R_f = 0.2 (pentane/ethyl acetate = 2:1 v/v).

^{1}H NMR (500 MHz, $CDCl_3$) δ 3.88 (s, 1H), 3.76 (s, 6H), 3.46 (t, *J* = 7.1 Hz, 4H), 2.15 (p, *J* = 7.2 Hz, 2H). **^{13}C NMR** (126 MHz, $CDCl_3$) δ 167.0, 71.4, 53.7, 52.5, 18.0. **IR** (neat) 2957, 2845, 1735, 1435, 1343, 1268, 1198, 1155, 1059, 1015, 915, 832, 749, 656, 591 cm^{-1}. **HRMS (ESI)** Calculated for $C_8H_{14}NO_4$ $[M+H]^+$: 188.0918; Found: 188.0928 m/z.

Dimethyl 2-(pyrrolidin-1-yl)malonate (3ac) [6]

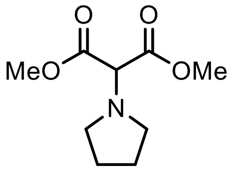

Following the general procedure A with dimethyl 2-diazomalonate **1a** (79 mg, 0.5 mmol, 1.0 equiv), pyrrolidine **2c** (36 mg, 0.5 mmol, 1.0 equiv), $[Ir(cod)Cl]_2$ (3.3 mg, 0.005 mmol, 1 mol%) and DCM (1.0 mL), the reaction was performed at 25 °C for 48 h. The mixture was purified by column chromatography to afford the desired product **3ac** as colorless oil (64 mg, 64% yield). R_f = 0.5 (pentane/ethyl acetate = 1:1 v/v).

^{1}H NMR (500 MHz, $CDCl_3$) δ 4.18 (s, 1H), 3.79 (s, 6H), 2.85 – 2.79 (m, 4H), 1.87 – 1.82 (m, 4H). **^{13}C NMR** (126 MHz, $CDCl_3$) δ 167.9, 68.8, 52.4, 50.7, 23.9. **IR** (neat) 2956, 1734, 1641, 1435, 1370, 1200, 1151, 1016, 904, 743 cm^{-1}. **HRMS (ESI)** Calculated for $C_9H_{16}NO_4$ $[M+H]^+$: 202.1074; Found: 202.1074 m/z.

Dimethyl 2-(piperidin-1-yl)malonate (3ad) [6]

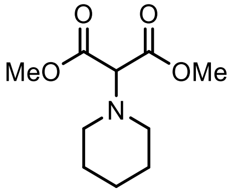

Following the general procedure A with dimethyl 2-diazomalonate **1a** (79 mg, 0.5 mmol, 1.0 equiv), piperidine **2d** (43 mg, 0.5 mmol, 1.0 equiv), $[Ir(cod)Cl]_2$ (3.3 mg, 0.005 mmol, 1 mol%) and DCM (1.0 mL), the reaction was performed at 25 °C for 15 h. The mixture was purified by column chromatography to afford the desired product **3ad** as colorless oil (85 mg, 79% yield). R_f = 0.7 (pentane/ethyl acetate = 2:1 v/v).

^{1}H NMR (500 MHz, $CDCl_3$) δ 4.06 (s, 1H), 3.78 (s, 6H), 2.70 – 2.64 (m, 4H), 1.67 – 1.61 (m, 4H), 1.50 – 1.41 (m, 2H). **^{13}C NMR** (126 MHz, $CDCl_3$) δ 167.8, 71.7, 52.2, 51.5, 26.2, 24.0. **IR** (neat)

2936, 2852, 1732, 1436, 1390, 1311, 1240, 1194, 1150, 1121, 1107, 1068, 1026, 974, 945, 861, 767, 713 cm^{-1}. **HRMS (ESI)** Calculated for $C_{10}H_{18}NO_4$ $[M+H]^+$: 216.1231; Found: 216.1232 m/z.

Dimethyl 2-(azepan-1-yl)malonate (3ae) [6]

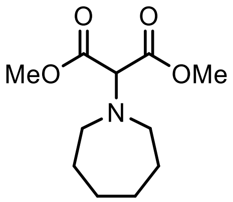

Following the general procedure A with dimethyl 2-diazomalonate **1a** (79 mg, 0.5 mmol, 1.0 equiv), azepane **2e** (50 mg, 0.5 mmol, 1.0 equiv), $[Ir(cod)Cl]_2$ (3.3 mg, 0.005 mmol, 1 mol%) and DCM (1.0 mL), the reaction was performed at 25 °C for 15 h. The mixture was purified by column chromatography to afford the desired product **3ae** as colorless oil (90 mg, 79% yield). R_f = 0.8 (pentane/ethyl acetate = 2:1 v/v).

^{1}H NMR (500 MHz, $CDCl_3$) δ 4.19 (s, 1H), 3.77 (s, 6H), 2.89 – 2.80 (m, 4H), 1.70 – 1.63 (m, 4H), 1.62 – 1.57 (m, 4H). **^{13}C NMR** (126 MHz, $CDCl_3$) δ 168.9, 71.6, 52.9, 52.2, 29.4, 27.1. **IR** (neat) 2925, 2852, 1733, 1435, 1397, 1306, 1218, 1194, 1148, 1094, 1056, 1018, 934, 740, 697 cm^{-1}. **HRMS (ESI)** Calculated for $C_{11}H_{20}NO_4$ $[M+H]^+$: 230.1387; Found: 230.1401 m/z.

Dimethyl 2-(3,4-dihydroisoquinolin-2(1H)-yl)malonate (3af) [7]

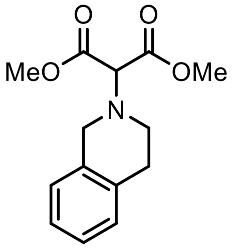

Following the general procedure A with dimethyl 2-diazomalonate **1a** (79 mg, 0.5 mmol, 1.0 equiv), 1,2,3,4-tetrahydroisoquinoline **2f** (67 mg, 0.5 mmol, 1.0 equiv), $[Ir(cod)Cl]_2$ (3.3 mg, 0.005 mmol, 1 mol%) and DCM (1.0 mL), the reaction was performed at 25 °C for 72 h. The mixture was purified by column chromatography to afford the desired product **3af** as colorless oil (101 mg, 77% yield). R_f = 0.6 (pentane/ethyl acetate = 2:1 v/v).

^{1}H NMR (500 MHz, $CDCl_3$) δ 7.16 – 7.08 (m, 3H), 7.04 – 6.98 (m, 1H), 4.33 (s, 1H), 3.98 (s, 2H), 3.81 (s, 6H), 3.06 (t, J = 5.8 Hz, 2H), 2.95 (t, J = 5.9 Hz, 2H). **^{13}C NMR** (126 MHz, $CDCl_3$) δ 167.7,

134.3, 134.0, 128.8, 126.4, 126.2, 125.6, 70.3, 52.7, 52.4, 47.9, 29.7. **IR** (neat) 2954, 2839, 1732, 1498, 1434, 1391, 1373, 1296, 1228, 1196, 1149, 1103, 1022, 1007, 936, 745 cm^{-1}. **HRMS (ESI)** Calculated for $C_{14}H_{18}NO_4$ $[M+H]^+$: 264.1231; Found: 264.1220 m/z.

Dimethyl 2-(4-oxopiperidin-1-yl)malonate (3ag)

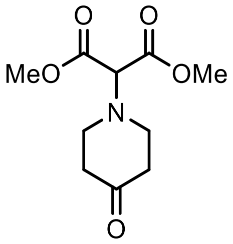

Following the general procedure A with dimethyl 2-diazomalonate **1a** (79 mg, 0.5 mmol, 1.0 equiv), piperidin-4-one **2g** (50 mg, 0.5 mmol, 1.0 equiv), $[Ir(cod)Cl]_2$ (3.3 mg, 0.005 mmol, 1 mol%) and DCM (1.0 mL), the reaction was performed at 25 °C for 15 h. The mixture was purified by column chromatography to afford the desired product **3ag** as yellow oil (68 mg, 59% yield). R_f = 0.4 (pentane/ethyl acetate = 1:1 v/v).

^{1}H NMR (500 MHz, $CDCl_3$) δ 4.26 (s, 1H), 3.80 (s, 6H), 3.07 (t, J = 6.1 Hz, 4H), 2.52 (t, J = 6.2 Hz, 4H). **^{13}C NMR** (126 MHz, $CDCl_3$) δ 208.0, 167.4, 69.9, 52.5, 50.0, 41.7. **IR** (neat) 2957, 2848, 1731, 1713, 1435, 1389, 1346, 1297, 1256, 1234, 1196, 1148, 1083, 1028, 1005, 899, 755, 598, 550, 504 cm^{-1}. **HRMS (ESI)** Calculated for $C_{10}H_{15}NO_5Na$ $[M+Na]^+$: 252.0843; Found: 252.0851 m/z.

Tetramethyl 2,2'-(piperazine-1,4-diyl)dimalonate (3ah)

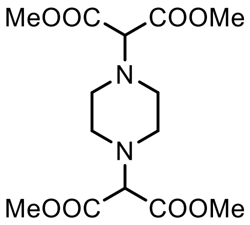

Following the general procedure A with dimethyl 2-diazomalonate **1a** (79 mg, 0.5 mmol, 2.0 equiv), piperazine **2h** (22 mg, 0.25 mmol, 1.0 equiv), $[Ir(cod)Cl]_2$ (3.3 mg, 0.005 mmol, 2 mol%) and DCM (1.0 mL), the reaction was performed at 25 °C for 15 h. The mixture was purified by column chromatography to afford the desired product **3ah** as white solid (75 mg, 87% yield). R_f = 0.2 (pentane/ethyl acetate = 2:1 v/v). M.p.: 145-147 °C.

^{1}H NMR (400 MHz, $CDCl_3$) 4.07 (s, 2H), 3.77 (s, 12H), 2.83 (s, 8H). **^{13}C NMR** (101 MHz, $CDCl_3$) δ 167.2, 70.5, 52.3, 50.3. **IR** (neat) 2954, 2852, 2824, 1754, 1727, 1438, 1395, 1351, 1318, 1301, 1274, 1215, 1164, 1129, 1033, 1009, 975, 918, 891, 840, 794, 732, 705 cm^{-1}. **HRMS (ESI)** Calculated for $C_{14}H_{23}N_2O_8$ $[M+H]^+$: 347.1449; Found: 347.1431 m/z.

Dimethyl 2-(diethylamino)malonate (3ai)

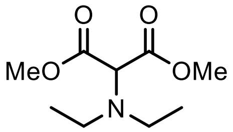

Following the general procedure A with dimethyl 2-diazomalonate **1a** (79 mg, 0.5 mmol, 1.0 equiv), diethylamine **2i** (37 mg, 0.5 mmol, 1.0 equiv), $[Ir(cod)Cl]_2$ (3.3 mg, 0.005 mmol, 1 mol%) and DCM (1.0 mL), the reaction was performed at 25 °C for 72 h. The mixture was purified by column chromatography to afford the desired product **3ai** as colorless oil (82 mg, 81% yield). R_f = 0.6 (pentane/ethyl acetate = 2:1 v/v).

^{1}H NMR (500 MHz, $CDCl_3$) 4.32 (s, 1H), 3.77 (s, 6H), 2.74 (q, J = 7.2 Hz, 4H), 1.09 (t, J = 7.1 Hz, 6H). **^{13}C NMR** (126 MHz, $CDCl_3$) δ 168.8, 66.9, 52.2, 45.8, 13.6. **IR** (neat) 2972, 1734, 1436, 1385, 1298, 1209, 1151, 1115, 1090, 1020, 908, 794, 753, 699 cm^{-1}. **HRMS (ESI)** Calculated for $C_9H_{18}NO_4$ $[M+H]^+$: 204.1231; Found: 204.1231 m/z.

Dimethyl 2-(diisopropylamino)malonate (3aj) [8]

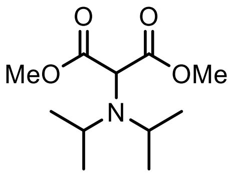

Following the general procedure A with dimethyl 2-diazomalonate **1a** (79 mg, 0.5 mmol, 1.0 equiv), diisopropylamine **2j** (56 mg, 0.5 mmol, 1.0 equiv), $[Ir(cod)Cl]_2$ (3.3 mg, 0.005 mmol, 1 mol%) and DCM (1.0 mL), the reaction was performed at 60 °C for 36 h. The mixture was purified by column chromatography to afford the desired product **3aj** as yellow oil (14 mg, 12% yield). R_f = 0.8 (pentane/ethyl acetate = 2:1 v/v).

^{1}H NMR (400 MHz, $CDCl_3$) δ 4.32 (s, 1H), 3.75 (s, 6H), 3.32 (hept, J = 6.7 Hz, 2H), 1.06 (d, J = 6.6 Hz, 12H). **^{13}C NMR** (101 MHz, $CDCl_3$) δ 171.3, 61.5, 52.2, 46.8, 22.2. **IR** (neat) 2964, 2872, 1761, 1736, 1643, 1460, 1435, 1408, 1391, 1363, 1282, 1220, 1187, 1141, 1093, 1028, 1013,

927, 850, 795, 739, 679 cm^{-1}. **HRMS (ESI)** Calculated for $C_{11}H_{22}NO_4$ $[M+H]^+$: 232.1544; Found: 232.1553 m/z.

Dimethyl 2-(2-methylpyrrolidin-1-yl)malonate (3ak)

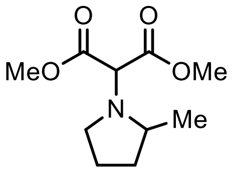

Following the general procedure A with dimethyl 2-diazomalonate **1a** (79 mg, 0.5 mmol, 1.0 equiv), 2-methylpyrrolidine **2k** (43 mg, 0.5 mmol, 1.0 equiv), $[Ir(cod)Cl]_2$ (3.3 mg, 0.005 mmol, 1 mol%) and DCM (1.0 mL), the reaction was performed at 60 °C for 36 h. The mixture was purified by column chromatography to afford the desired product **3ak** as yellow oil (63 mg, 59% yield). R_f = 0.8 (pentane/ethyl acetate = 1:1 v/v).

^{1}H NMR (500 MHz, $CDCl_3$) δ 4.38 (s, 1H), 3.78 (s, 3H), 3.76 (s, 3H), 3.21 (ddd, *J* = 9.1, 7.8, 4.4 Hz, 1H), 2.97 (dq, *J* = 13.1, 6.1 Hz, 1H), 2.90 – 2.83 (m, 1H), 1.99 – 1.91 (m, 1H), 1.86 – 1.78 (m, 1H), 1.78 – 1.69 (m, 1H), 1.50 – 1.42 (m, 1H), 1.09 (d, *J* = 6.1 Hz, 3H). **^{13}C NMR** (126 MHz, $CDCl_3$) δ 168.6, 168.2, 64.9, 56.6, 52.5, 51.9, 49.2, 32.9, 22.7, 19.2. **IR** (neat) 2957, 2871, 1734, 1435, 1377, 1288, 1211, 1151, 1022, 945, 919, 895, 748, 585, 483 cm^{-1}. **HRMS (ESI):** Calculated for $C_{10}H_{18}NO_4$ $[M+H]^+$: 216.1231; Found: 216.1225 m/z.

Dimethyl 2-(2-methylpiperidin-1-yl)malonate (3al)

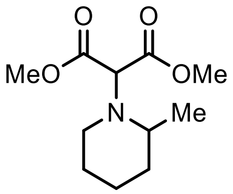

Following the general procedure A with dimethyl 2-diazomalonate **1a** (79 mg, 0.5 mmol, 1.0 equiv), 2-methylpiperidine **2l** (50 mg, 0.5 mmol, 1.0 equiv), $[Ir(cod)Cl]_2$ (3.3 mg, 0.005 mmol, 1 mol%) and DCM (1.0 mL), the reaction was performed at 60 °C for 15 h. The mixture was purified by column chromatography to afford the desired product **3al** as pale yellow oil (63 mg, 55% yield). R_f = 0.7 (pentane/ethyl acetate = 1:1 v/v).

^{1}H NMR (500 MHz, $CDCl_3$) δ 4.54 (s, 1H), 3.77 (s, 3H), 3.76 (s, 3H), 3.05 (dtd, *J* = 11.4, 3.6, 1.5 Hz, 1H), 2.58 (dtt, *J* = 12.6, 6.3, 3.2 Hz, 1H), 2.48 (td, *J* = 11.4, 2.9 Hz, 1H), 1.73 – 1.49 (m, 4H),

1.44 – 1.35 (m, 1H), 1.35 – 1.26 (m, 1H), 1.11 (d, J = 6.3 Hz, 3H). **^{13}C NMR** (126 MHz, $CDCl_3$) δ 168.9, 167.9, 66.0, 54.9, 52.5, 51.7, 49.5, 35.2, 26.4, 24.5, 19.8. **IR** (neat) 2933, 2853, 1733, 1435, 1378, 1294, 1231, 1191, 1151, 1135, 1116, 1091, 1061, 1018, 932, 903, 825, 757, 697, 602, 533, 460 cm^{-1}. **HRMS (ESI)** Calculated for $C_{11}H_{20}NO_4$ $[M+H]^+$: 230.1387; Found: 230.1402 m/z.

Dimethyl 2-(3,4-dihydroquinolin-1(2H)-yl)malonate (3am)

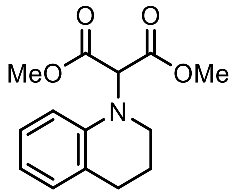

Following the general procedure A with dimethyl 2-diazomalonate **1a** (79 mg, 0.5 mmol, 1.0 equiv), 1,2,3,4-tetrahydroquinoline **2m** (67 mg, 0.5 mmol, 1.0 equiv), $[Ir(cod)Cl]_2$ (3.3 mg, 0.005 mmol, 1 mol%) and DCM (1.0 mL), the reaction was performed at 25 °C for 72 h. The mixture was purified by column chromatography to afford the desired product **3am** as colorless oil (103 mg, 78% yield). R_f = 0.7 (pentane/ethyl acetate = 2:1 v/v).

^{1}H NMR (500 MHz, $CDCl_3$) δ 7.05 (t, J = 7.8 Hz, 1H), 7.01 (d, J = 7.5 Hz, 1H), 6.69 (t, J = 7.3 Hz, 1H), 6.54 (d, J = 8.2 Hz, 1H), 5.20 (s, 1H), 3.81 (s, 6H), 3.43 – 3.36 (m, 2H), 2.84 – 2.77 (m, 2H), 2.04 – 1.94 (m, 2H). **^{13}C NMR** (126 MHz, $CDCl_3$) δ 168.2, 144.0, 129.7, 127.1, 124.0, 117.9, 110.5, 64.4, 52.8, 46.3, 27.8, 22.2. **IR** (neat) 2952, 2846, 1736, 1603, 1578, 1497, 1456, 1434, 1346, 1305, 1247, 1223, 1193, 1154, 1117, 1061, 1029, 1013, 981, 945, 899, 874, 821, 799, 744, 712, 684, 612, 542, 484 cm^{-1}. **HRMS (ESI)** Calculated for $C_{14}H_{18}NO_4$ $[M+H]^+$: 264.1231; Found: 264.1220 m/z.

Dimethyl 2-(phenylamino)malonate (3an)

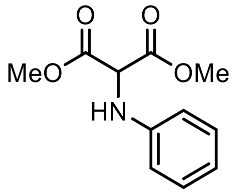

Following the general procedure A with dimethyl 2-diazomalonate **1a** (79 mg, 0.5 mmol, 1.0 equiv), aniline **2n** (47 mg, 0.5 mmol, 1.0 equiv), $[Ir(cod)Cl]_2$ (3.3 mg, 0.005 mmol, 1 mol%) and DCM (1.0 mL), the reaction was performed at 25 °C for i) 15 h or ii) 72 h. The mixture was purified by column

chromatography to afford the desired product **3an** as colorless oil, yielding i) 78 mg (70% yield) or ii) 96 mg (86% yield). R_f = 0.8 (pentane/ethyl acetate = 2:1 v/v).

^{1}H NMR (500 MHz, $CDCl_3$) δ 7.25 – 7.18 (m, 2H), 6.83 – 6.78 (m, 1H), 6.69 – 6.65 (m, 2H), 4.86 – 4.79 (m, 2H), 3.83 (s, 6H). **^{13}C NMR** (126 MHz, $CDCl_3$) δ 168.2, 145.3, 129.5, 119.2, 113.5, 60.5, 53.3. **IR** (neat) 3393, 3028, 2956, 1735, 1604, 1507, 1434, 1293, 1260, 1221, 1154, 1078, 1012, 972, 936, 877, 841, 750, 692 cm^{-1}. **HRMS (ESI)** Calculated for $C_{11}H_{14}NO_4$ $[M+H]^+$: 224.0918; Found: 224.0921 m/z.

Dimethyl 2-((4-methoxyphenyl)amino)malonate (3ao)

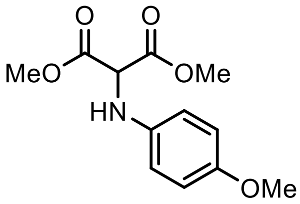

Following the general procedure A with dimethyl 2-diazomalonate **1a** (79 mg, 0.5 mmol, 1.0 equiv), 4-methoxyaniline **2o** (62 mg, 0.5 mmol, 1.0 equiv), $[Ir(cod)Cl]_2$ (3.3 mg, 0.005 mmol, 1 mol%) and DCM (1.0 mL), the reaction was performed at 25 °C for 15 h. The mixture was purified by column chromatography to afford the desired product **3ao** as yellow solid (113 mg, 89% yield). R_f = 0.6 (pentane/ethyl acetate = 2:1 v/v). M.p.: 75-76 °C.

^{1}H NMR (500 MHz, $CDCl_3$) δ 6.81 – 6.77 (m, 2H), 6.66 – 6.62 (m, 2H), 4.74 (s, 1H), 4.56 (br, 1H), 3.82 (s, 6H), 3.75 (s, 3H). **^{13}C NMR** (126 MHz, $CDCl_3$) δ 168.3, 153.3, 139.3, 115.2, 115.0, 61.6, 55.7, 53.3. **IR** (neat) 3401, 2955, 2834, 1731, 1591, 1516, 1435, 1324, 1300, 1260, 1239, 1215, 1184, 1160, 1113, 1076, 1038, 1009, 995, 942, 847, 829, 804, 769, 677, 597, 536, 513, 457 cm^{-1}. **HRMS (ESI)** Calculated for $C_{12}H_{15}NO_5Na$ $[M+Na]^+$: 276.0843; Found: 276.0855 m/z.

Dimethyl 2-((4-(trifluoromethyl)phenyl)amino)malonate (3ap)

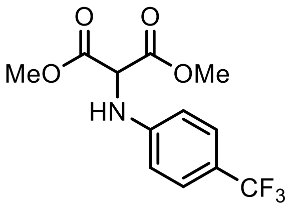

Following the general procedure A with dimethyl 2-diazomalonate **1a** (79 mg, 0.5 mmol, 1.0 equiv), 4-(trifluoromethyl)aniline **2p** (81 mg, 0.5 mmol, 1.0 equiv), $[Ir(cod)Cl]_2$ (3.3 mg, 0.005 mmol, 1

mol%) and DCM (1.0 mL), the reaction was performed at 25 °C for 15 h. The mixture was purified by column chromatography to afford the desired product **3ap** as yellow solid (135 mg, 93% yield). R_f = 0.6 (pentane/ethyl acetate = 2:1 v/v). M.p.: 83-84 °C.

^{1}H NMR (500 MHz, $CDCl_3$) δ 7.48 – 7.41 (m, 2H), 6.70 – 6.64 (m, 2H), 5.16 (d, *J* = 7.4 Hz, 1H), 4.83 (d, *J* = 7.4 Hz, 1H), 3.85 (s, 6H). **^{13}C NMR** (126 MHz, $CDCl_3$) δ 167.5, 147.7, 126.9 (q, *J* = 3.9 Hz), 124.6 (q, *J* = 270.6 Hz), 120.8 (q, *J* = 32.6 Hz), 112.7, 59.8, 53.5. **^{19}F NMR** (282 MHz, $CDCl_3$) δ -61.4. **IR** (neat) 3376, 2961, 1728, 1618, 1592, 1533, 1491, 1446, 1437, 1418, 1317, 1293, 1267, 1225, 1195, 1181, 1150, 1106, 1066, 1006, 990, 961, 942, 858, 828, 749, 673, 629, 591, 534, 506 cm^{-1}. **HRMS (ESI)** Calculated for $C_{12}H_{13}F_3NO_4$ $[M+H]^+$: 292.0792; Found: 292.0785 m/z.

Dimethyl 2-(benzylamino)malonate (3aq) [9]

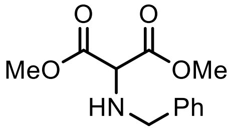

Following the general procedure A with dimethyl 2-diazomalonate **1a** (79 mg, 0.5 mmol, 1.0 equiv), phenylmethanamine **2q** (54 mg, 0.5 mmol, 1.0 equiv), $[Ir(cod)Cl]_2$ (3.3 mg, 0.005 mmol, 1 mol%) and DCM (1.0 mL), the reaction was performed at 60 °C for 36 h. The mixture was purified by column chromatography to afford the desired product **3aq** as yellow oil (96 mg, 81% yield). R_f = 0.5 (pentane/ethyl acetate = 2:1 v/v).

^{1}H NMR (500 MHz, $CDCl_3$) δ 7.36 – 7.31 (m, 4H), 7.30 – 7.25 (m, 1H), 4.11 (s, 1H), 3.82 (s, 2H), 3.78 (s, 6H), 2.47 (br, 1H). **^{13}C NMR** (126 MHz, $CDCl_3$) δ 168.9, 138.5, 128.5, 128.4, 127.4, 63.8, 52.8, 51.7. **IR** (neat) 3344, 3029, 2954, 2848, 1735, 1496, 1454, 1435, 1376, 1290, 1214, 1152, 1082, 1014, 912, 735, 698, 583, 491 cm^{-1}. **HRMS (ESI)** Calculated for $C_{12}H_{16}NO_4$ $[M+H]^+$: 238.1074; Found: 238.1084 m/z.

Dimethyl 2-(hexylamino)malonate (3ar)

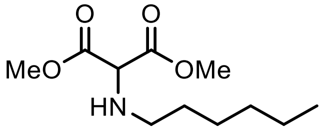

Following the general procedure A with dimethyl 2-diazomalonate **1a** (79 mg, 0.5 mmol, 1.0 equiv), hexan-1-amine **2r** (51 mg, 0.5 mmol, 1.0 equiv), $[Ir(cod)Cl]_2$ (3.3 mg, 0.005 mmol, 1 mol%) and

DCM (1.0 mL), the reaction was performed at 60 °C for 15 h. The mixture was purified by column chromatography to afford the desired product **3ar** as colorless oil (106 mg, 92% yield).

92% yield. Colorless oil. R_f = 0.4 (pentane/ethyl acetate = 2:1 v/v). **^{1}H NMR** (500 MHz, $CDCl_3$) δ 4.08 (s, 1H), 3.79 (s, 6H), 2.60 – 2.55 (m, 2H), 1.54 – 1.47 (m, 2H), 1.34 – 1.26 (m, 6H), 0.90 – 0.86 (m, 3H). **^{13}C NMR** (126 MHz, $CDCl_3$) δ 169.1, 64.9, 52.8, 48.1, 31.7, 29.9, 26.8, 22.6, 14.0. **IR** (neat) 2955, 2929, 2857, 1738, 1435, 1379, 1198, 1153, 1075, 1016, 919, 771, 726, 600 cm^{-1}. **HRMS (ESI)** Calculated for $C_{11}H_{22}NO_4$ $[M+H]^+$: 232.1544; Found: 232.1553 m/z.

4.3 Gram-scale experiment

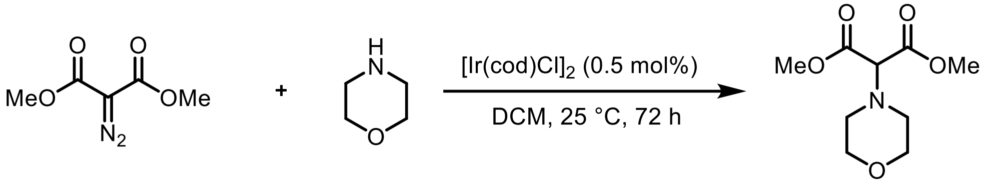

1a **2a** **3aa**

In a 50 mL flask equipped with a magnetic stirring bar, diazomalonate **1a** (1.58 g, 10 mmol, 1.0 equiv) was added to the solution of morpholine **2a** (870 mg, 10 mmol, 1.0 equiv) and $[Ir(cod)Cl]_2$ (34 mg, 0.05 mmol, 0.5 mol%) dissolved in DCM (20 mL). The mixture was stirred at 25 °C for 72 h. After full conversion of diazo reagents confirmed by TLC, the solution was concentrated using rotary evaporator and the residue was purified by column chromatography to afford the N-H insertion adduct **3aa** as white solid (1.98 g, 91% yield).

5. Post-transformation

5.1 Synthesis of dimethyl 2-(hydroxymethyl)-2-morpholinomalonate (4)

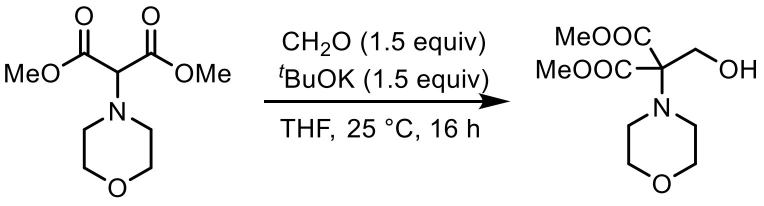

3aa **4**

In a 50 mL flask equipped with a magnetic stirring bar, **3aa** (304 mg, 1.4 mmol, 1.0 equiv) were dissolved in THF (14 mL). Then, add ^{t}BuOK (235 mg, 2.1 mmol, 1.5 equiv) in one portion. After stirring at 25 °C for 10 min, to this solution formaldehyde (126 mg, 4.2 mmol, 1.5 equiv) was added slowly. The mixture was stirred at 25 °C for 15 h. After reaction, the mixture was quenched with water and extracted with ethyl acetate (3x10 mL). The organic phase was collected and dried with anhydrous Na_2SO_4. Then, the organic solution was concentrated in vacuum and the residue was purified by column chromatography to afford the desired product **4** as white solid (163 mg, 47% yield). R_f = 0.2 (pentane/ethyl acetate = 2:1 v/v). M.p.: 70-72 °C.

^{1}H NMR (500 MHz, $CDCl_3$) δ 4.03 (d, J = 6.1 Hz, 2H), 3.81 (s, 6H), 3.78 – 3.71 (m, 4H), 2.80 – 2.73 (m, 4H), 2.71 (d, J = 6.2 Hz, 1H). **^{13}C NMR** (126 MHz, $CDCl_3$) δ 168.7, 75.5, 67.4, 62.8, 52.5, 48.3. **IR** (neat) 3423, 2992, 2960 ,2920, 2852, 2819, 1757, 1726, 1490, 1440, 1378, 1334, 1271, 1238, 1202, 1142, 1112, 1088, 1052, 1022, 979, 959, 924, 861, 841, 814, 788, 755, 703 cm^{-1}. **HRMS (ESI)** Calculated for $C_{10}H_{18}NO_6$ $[M+H]^+$: 248.1129; Found: 248.1140 m/z.

5.2 Synthesis of dimethyl 2-methyl-2-morpholinomalonate (5)

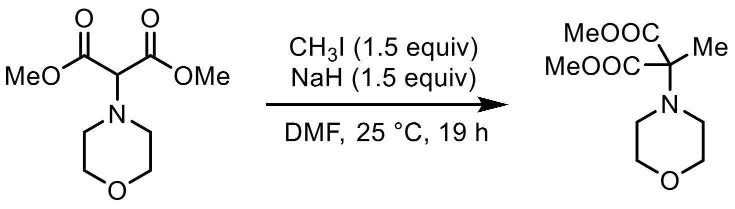

3aa **5**

NaH (300 mg, 7.5 mmol, 1.5 equiv, 60% in oil) and dry DMF (5.0 mL) were mixed in a 25 mL flask equipped with a magnetic stirring bar under nitrogen. To this solution a mixture of **3aa** (1.09 g, 5.0 mmol, 1.0 equiv) and DMF (5.0 mL) was added slowly at 0 °C. After stirring at 25 °C for 1 h,

iodomethane (1.06 g, 7.5 mmol, 1.5 equiv) was added to this solution at 0 °C. Then, the mixture was stirred at 25 °C for 19 h. After reaction, the mixture was quenched with water and extracted with ethyl acetate (3x10 mL). The organic phase was collected and dried with anhydrous Na_2SO_4. Then, the organic solution was concentrated in vacuum and the residue was purified by column chromatography to afford the desired product **5** as colorless oil (670 mg, 58% yield). R_f = 0.4 (pentane/ethyl acetate = 2:1 v/v).

^{1}H NMR (500 MHz, $CDCl_3$) δ 3.77 (s, 6H), 3.77 – 3.74 (m, 4H), 2.69 – 2.65 (m, 4H), 1.59 (s, 3H). **^{13}C NMR** (126 MHz, $CDCl_3$) δ 170.1, 71.8, 67.4, 52.4, 48.2, 19.3. **IR** (neat) 2955, 2854, 1728, 1451, 1370, 1257, 1194, 1160, 1103, 1070, 1032, 977, 924, 886, 864, 777, 710 cm^{-1}. **HRMS (ESI)** Calculated for $C_{10}H_{17}NO_5Na$ [M+Na]$^+$: 254.0999; Found: 254.0995 m/z.

5.3 Synthesis of dimethyl 2-morpholino-2-(prop-2-yn-1-yl)malonate (6) [5]

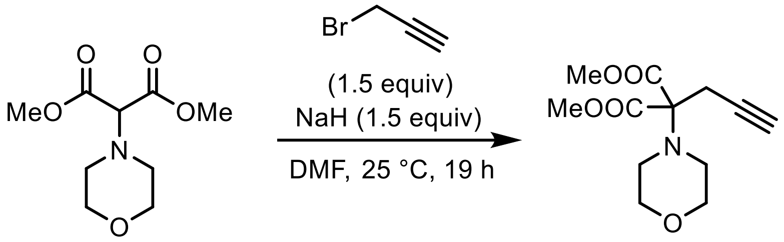

3aa **6**

NaH (60 mg, 1.5 mmol, 1.5 equiv, 60% in oil) and dry DMF (1.0 mL) were mixed in a 25 mL flask equipped with a magnetic stirring bar under nitrogen. To this solution a mixture of **3aa** (217 mg, 1.0 mmol, 1.0 equiv) and DMF (1.0 mL) was added slowly at 0 °C. After stirring at 25 °C for 1 h, propargyl bromide (179 mg, 1.5 mmol, 1.5 equiv) was added to this solution at 0 °C. Then, the mixture was stirred at 25 °C for 19 h. After reaction, the mixture was quenched with water and extracted with ethyl acetate (3x10 mL). The organic phase was collected and dried with anhydrous Na_2SO_4. Then, the organic solution was concentrated in vacuum and the residue was purified by column chromatography to afford the desired product **6** as yellow solid (196 mg, 77% yield). R_f = 0.6 (pentane/ethyl acetate = 1:1 v/v). M.p.: 65-67 °C.

^{1}H NMR (500 MHz, $CDCl_3$) δ 3.80 (s, 6H), 3.79 – 3.76 (m, 4H), 2.99 (d, *J* = 2.7 Hz, 2H), 2.74 – 2.67 (m, 4H), 2.08 (t, *J* = 2.7 Hz, 1H). **^{13}C NMR** (126 MHz, $CDCl_3$) δ 168.3, 78.0, 74.2, 71.9, 67.3, 52.5, 48.6, 23.7. **IR** (neat) 3292, 2963, 2859, 2832, 1755, 1722, 1450, 1432, 1269, 1242, 1198, 1175, 1148, 1115, 1079, 1053, 1027, 975, 939, 926, 859, 783, 751, 690, 659, 602 cm^{-1}. **HRMS (ESI)** Calculated for $C_{12}H_{18}NO_5$ [M+H]$^+$: 256.1180; Found: 256.1193 m/z.

5.4 Synthesis of methyl 2-morpholinoacetate (7) [10]

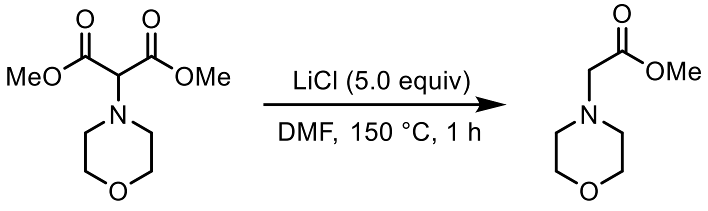

3aa **7**

In a 2 mL flask equipped with a magnetic stirring bar, **3aa** (43 mg, 0.2 mmol, 1.0 equiv) and LiCl (43 mg, 1.0 mmol, 5.0 equiv) were dissolved in DMF (1.0 mL) The mixture was stirring at 150 °C for 1 h. After reaction, 77% NMR yield was obtained (1,3,5-trimethoxylbenzene as internal reference). Then, the mixture was diluted with water and extracted with ethyl acetate. The organic phases were collected and dried with anhydrous Na_2SO_4. After concentrated by vacuum, a crude yellow oil was obtained with clean ^1H NMR spectroscopy. (The isolated yield was not precise due to volatility of the desired product **7**). R_f = 0.4 (ethyl acetate).

^{1}H NMR (400 MHz, $CDCl_3$) δ 3.79 – 3.75 (m, 4H), 3.74 (s, 3H), 3.23 (s, 2H), 2.62 – 2.56 (m, 4H). (Compound **7** has been previously reported in the literature.)[10]

5.5 Synthesis of N1,N3-dihexyl-2-morpholinomalonamide (8)

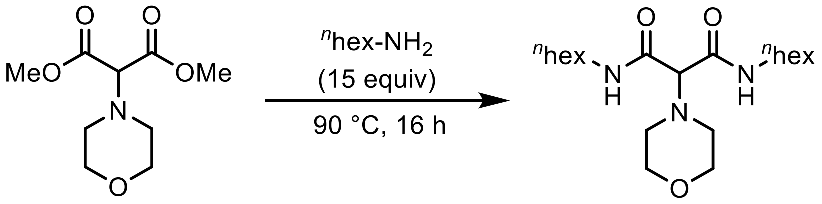

3aa **8**

In a 10 mL flask equipped with a magnetic stirring bar, **3aa** (217 mg, 1.0 mmol, 1.0 equiv) and hexylamine (1.52 g, 15 mmol, 15 equiv) were mixed. The mixture was stirring at 90 °C for 16 h. After reaction, the solution was concentrated in vacuum and the residue was purified by column chromatography to afford the desired product **8** as white solid (266 mg, 75% yield). R_f = 0.5 (ethyl acetate). M.p.: 111-113 °C.

^{1}H NMR (500 MHz, $CDCl_3$) δ 7.42 – 7.32 (m, 2H), 3.76 – 3.69 (m, 4H), 3.43 (s, 1H), 3.32 – 3.19 (m, 4H), 2.74 – 2.68 (m, 4H), 1.55 – 1.47 (m, 4H), 1.36 – 1.26 (m, 12H), 0.96 – 0.85 (m, 6H). **^{13}C NMR** (126 MHz, $CDCl_3$) δ 167.4, 73.3, 67.3, 51.0, 39.4, 31.4, 29.3, 26.6, 22.5, 14.0. **IR** (neat)

3300, 2957, 2927, 2856, 1740, 1659, 1524, 1436, 1367, 1295, 1275, 1246, 1230, 1202, 1157, 1120, 1092, 1078, 1026, 922, 886, 728, 679 cm^{-1}. **HRMS (ESI)** Calculated for $C_{19}H_{38}N_3O_3$ $[M+H]^+$: 356.2908; Found: 356.2910 m/z.

6. Late-stage reactivity

6.1 General procedure B

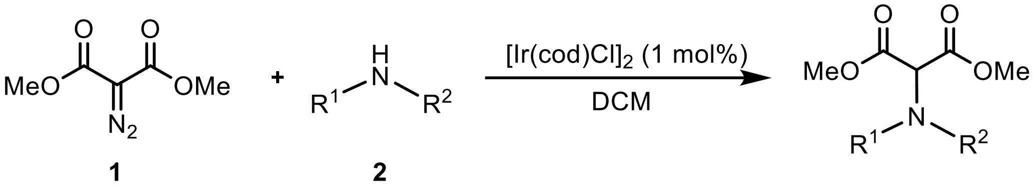

In a 2 mL screw-cap vial equipped with a magnetic stirring bar, the diazo compound **1** (0.25 mmol), amines **2** (0.25 mmol) and $[Ir(cod)Cl]_2$ (1 mol%) were dissolved in DCM (0.5 mL). The vial was capped and the solution was stirred at 25 °C for 20 h (with Amoxapine and Vortioxetine) or at 60 °C for 72 h (with Pomalidomide). The solution was concentrated in vacuum and the residue was purified by column chromatography to afford the desired product.

6.2 Data analysis

Dimethyl 2-(4-(2-chlorodibenzo[b,f][1,4]oxazepin-11-yl)piperazin-1-yl)malonate (9)

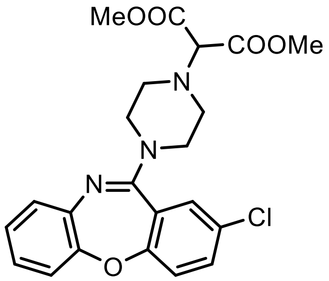

Following the general procedure B with dimethyl 2-diazomalonate **1a** (40 mg, 0.25 mmol, 1.0 equiv), Amoxapine (78 mg, 0.25 mmol, 1.0 equiv), $[Ir(cod)Cl]_2$ (1.7 mg, 0.0025 mmol, 1 mol%) and DCM (0.5 mL), the reaction was performed at 25 °C for 20 h. The mixture was purified by column chromatography to afford the desired product **9** as yellow solid (110 mg, 99% yield). R_f = 0.3 (pentane/ethyl acetate = 2:1 v/v). M.p.: 76-78 °C.

^{1}H NMR (500 MHz, $CDCl_3$) δ 7.39 (dd, *J* = 8.6, 2.6 Hz, 1H), 7.31 (d, *J* = 2.6 Hz, 1H), 7.19 (d, *J* = 8.7 Hz, 1H), 7.14 (dd, *J* = 7.8, 1.7 Hz, 1H), 7.11 – 7.06 (m, 2H), 7.01 – 6.97 (m, 1H), 4.17 (s, 1H), 3.81 (s, 6H), 3.68 – 3.49 (m, 4H), 3.02 – 2.80 (m, 4H). **^{13}C NMR** (126 MHz, $CDCl_3$) δ 167.3, 159.3, 158.8, 151.8, 140.1, 132.5, 130.3, 129.0, 127.1, 125.8, 124.9, 124.6, 122.7, 120.1, 70.5, 52.5, 50.0, 47.5. **IR** (neat) 2953, 2849, 2050, 1733, 1588, 1556, 1469, 1305, 1240, 1151, 1019, 934,

899, 830, 796, 774, 752, 676 cm^{-1}. **HRMS (ESI)** Calculated for $C_{22}H_{23}ClN_3O_5$ $[M+H]^+$: 444.1321; Found: 444.1311 m/z.

Dimethyl 2-(4-(2-((2,4-dimethylphenyl)thio)phenyl)piperazin-1-yl)malonate (10)

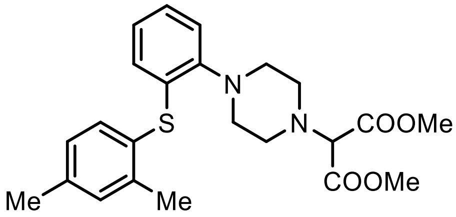

Following the general procedure B with dimethyl 2-diazomalonate **1a** (40 mg, 0.25 mmol, 1.0 equiv), Vortioxetine (75 mg, 0.25 mmol, 1.0 equiv), $[Ir(cod)Cl]_2$ (1.7 mg, 0.0025 mmol, 1 mol%) and DCM (0.5 mL), the reaction was performed at 25 °C for 20 h. The mixture was purified by column chromatography to afford the desired product **10** as white solid (105 mg, 98% yield). R_f = 0.6 (pentane/ethyl acetate = 2:1 v/v). M.p.: 109-110 °C.

^{1}H NMR (500 MHz, $CDCl_3$) δ 7.37 (d, *J* = 7.8 Hz, 1H), 7.17 – 7.14 (m, 1H), 7.10 – 7.06 (m, 2H), 7.05 – 7.00 (m, 1H), 6.87 (ddd, *J* = 8.0, 5.2, 3.5 Hz, 1H), 6.53 – 6.49 (m, 1H), 4.16 (s, 1H), 3.82 (s, 6H), 3.23 – 3.11 (m, 4H), 2.98 – 2.90 (m, 4H), 2.37 (s, 3H), 2.32 (s, 3H). **^{13}C NMR** (126 MHz, $CDCl_3$) δ 167.5, 149.0, 142.4, 139.2, 136.2, 134.6, 131.7, 128.0, 127.8, 126.2, 125.5, 124.4, 119.9, 70.9, 52.4, 51.7, 50.8, 21.2, 20.6. **IR** (neat) 2960, 2883, 2832, 1762, 1733, 1576, 1469, 1440, 1380, 1347, 1294, 1269, 1226, 1205, 1159, 1133, 1032, 1009, 919, 823, 765, 732, 686, 590, 554 cm^{-1}. **HRMS (ESI)** Calculated for $C_{23}H_{29}N_2O_4S$ $[M+H]^+$: 429.1843; Found: 429.1843 m/z.

Dimethyl 2-((2-(2,6-dioxopiperidin-3-yl)-1,3-dioxoisoindolin-4-yl)amino)malonate (11)

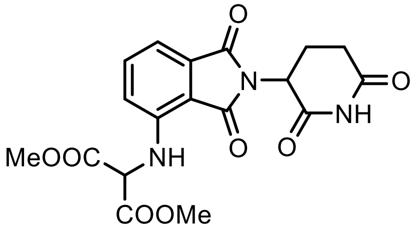

Following the general procedure B with dimethyl 2-diazomalonate **1a** (40 mg, 0.25 mmol, 1.0 equiv), Pomalidomide (68 mg, 0.25 mmol, 1.0 equiv), $[Ir(cod)Cl]_2$ (1.7 mg, 0.0025 mmol, 1 mol%) and DCM (0.5 mL), the reaction was performed at 60 °C for 72 h. The mixture was purified by column chromatography to afford the desired product **11** as yellow solid (82 mg, 81% yield). R_f = 0.8 (ethyl acetate). M.p.: 203-204 °C.

^{1}H NMR (500 MHz, CD_2Cl_2) δ 8.07 (s, 1H), 7.59 – 7.52 (m, 1H), 7.21 (d, *J* = 7.2 Hz, 1H), 7.20 (d, *J* = 7.1 Hz, 1H), 6.83 (d, *J* = 8.4 Hz, 1H), 4.98 – 4.90 (m, 2H), 3.84 (s, 3H), 3.84 (s, 3H), 2.93 – 2.70 (m, 3H), 2.21 – 2.09 (m, 1H). **^{13}C NMR** (126 MHz, CD_2Cl_2) δ 170.9, 169.0, 168.3, 167.2, 166.8, 166.8, 143.9, 136.4, 132.6, 116.9, 113.1, 112.0, 59.1, 53.6, 53.6, 49.1, 31.4, 22.6. **IR** (neat) 3373, 3251, 2960, 2163, 2050, 1980, 1732, 1694, 1624, 1507, 1435, 1405, 1339, 1295, 1254, 1161, 1115, 1023, 922, 881, 815, 745, 679, 597, 522, 467 cm^{-1}. **HRMS (ESI)** Calculated for $C_{18}H_{18}N_3O_8$ $[M+H]^+$: 404.1089; Found: 404.1077 m/z.

7. Synthesis and data analysis of aza-macrocycle

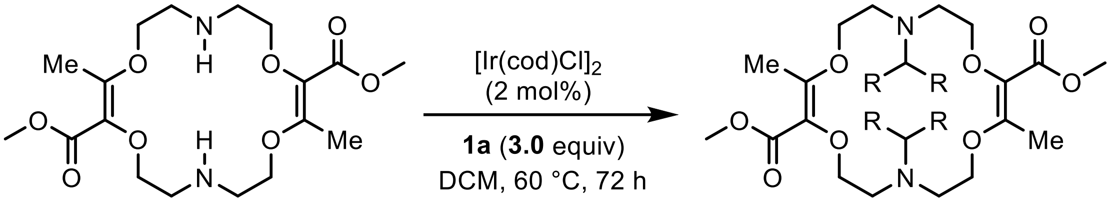

12 **13**, R = CO_2Me

In a 2 mL screw-cap vial equipped with a magnetic stirring bar, the diazo compound **1a** (47 mg, 0.3 mmol, 3.0 equiv), diaza-macrocycle **12** (40 mg, 0.1 mmol, 1.0 equiv)[11] and $[Ir(cod)Cl]_2$ (1.3 mg, 0.002 mmol, 2 mol%) were dissolved in DCM (0.6 mL). the vial was capped and the solution was stirred at 60 °C for 72 h. The solution was concentrated in vacuum and the residue was purified by column chromatography to afford the desired product **13** as yellow oil (32 mg, 48% yield). R_f = 0.3 (pentane/ethyl acetate = 1:1 v/v).

^{1}H NMR (500 MHz, $CDCl_3$) δ 4.46 (s, 2H), 4.16 – 4.12 (m, 4H), 3.81 – 3.79 (m, 4H), 3.77 (s, 12H), 3.73 (s, 6H), 3.22 – 3.14 (m, 8H), 2.31 (s, 6H). **^{13}C NMR** (126 MHz, $CDCl_3$) δ 168.8, 166.5, 158.4, 129.3, 71.3, 69.0, 68.0, 53.7, 52.5, 52.4, 51.3, 15.1. **IR** (neat) 2953, 1733, 1704, 1616, 1434, 1377, 1268, 1240, 1189, 1148, 1087, 1038, 775, 704, 601 cm^{-1}. **HRMS (ESI):** Calculated for $C_{28}H_{43}N_2O_{16}$ $[M+H]^+$: 663.2607; Found: 663.2627 m/z.

8. X-ray information

A suitable crystal was prepared in DCM/pentane (1:1) and X-ray intensity data were collected on a Rigaku XtaLAB Synergy, Dualflex, HyPix-Arc 150° diffractometer using Cu Kα radiation (λ = 1.54184 Å). Using Olex2,[12] the structure was solved with the SHELXT[13] structure solution program using dual space methods and refined with the SHELXL[14] refinement package using least squares minimization. Summaries of crystal data and structure refinement parameters are given in ***Table S3***.

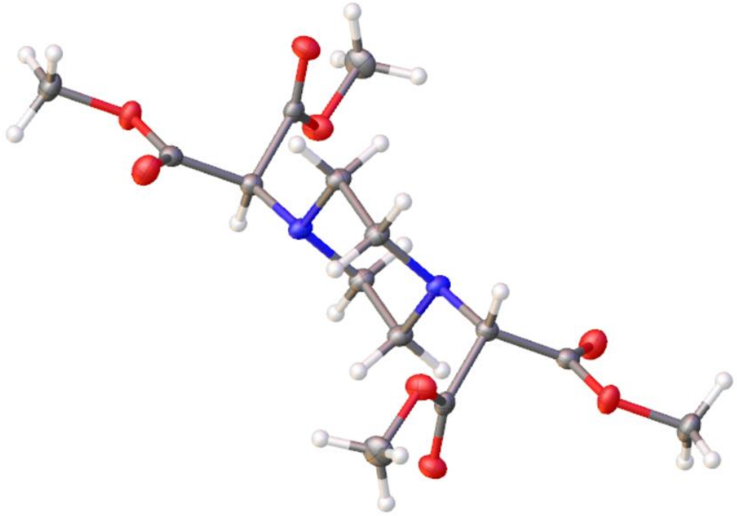

Fig S1. View of 3ah (the asymmetric unit is half a molecule) at 50 percent probability displacement ellipsoids.

Table S3. Crystal data and structure refinement for 3ah.

CCDC number	2295944	
Empirical formula	C14 H22 N2 O8	
Formula weight	346.33	
Temperature	100.01(11) K	
Wavelength	1.54184 Å	
Crystal system	Monoclinic	
Space group	P 1 21/n 1	
Unit cell dimensions	a = 8.22080(10) Å	α = 90°

	b = 9.95660(10) Å	β = 99.4410(10)°
	c = 10.1974(2) Å	γ = 90°
Volume	823.36(2) $Å^3$	
Z	2	
Density (calculated)	1.397 Mg/m^3	
Absorption coefficient	0.984 mm^{-1}	
F(000)	368	
Crystal size	0.345 x 0.244 x 0.191 mm^3	
Theta range for data collection	6.254 to 74.088°	
Index ranges	-10<=h<=10, -12<=k<=12, -12<=l<=11	
Reflections collected	17625	
Independent reflections	1652 [R(int) = 0.0177]	
Completeness to theta = 67.684°	100.0 %	
Absorption correction	Gaussian	
Max. and min. transmission	1.000 and 0.355	
Refinement method	Full-matrix least-squares on F^2	
Data / restraints / parameters	1652 / 0 / 112	
Goodness-of-fit on F^2	1.073	
Final R indices [I>2sigma(I)]	R1 = 0.0288, wR2 = 0.0743	
R indices (all data)	R1 = 0.0292, wR2 = 0.0746	
Extinction coefficient	0.0055(7)	
Largest diff. peak and hole	0.322 and -0.195 e.$Å^{-3}$	

9. NMR spectra

^{1}H NMR (400 MHz, $CDCl_3$)

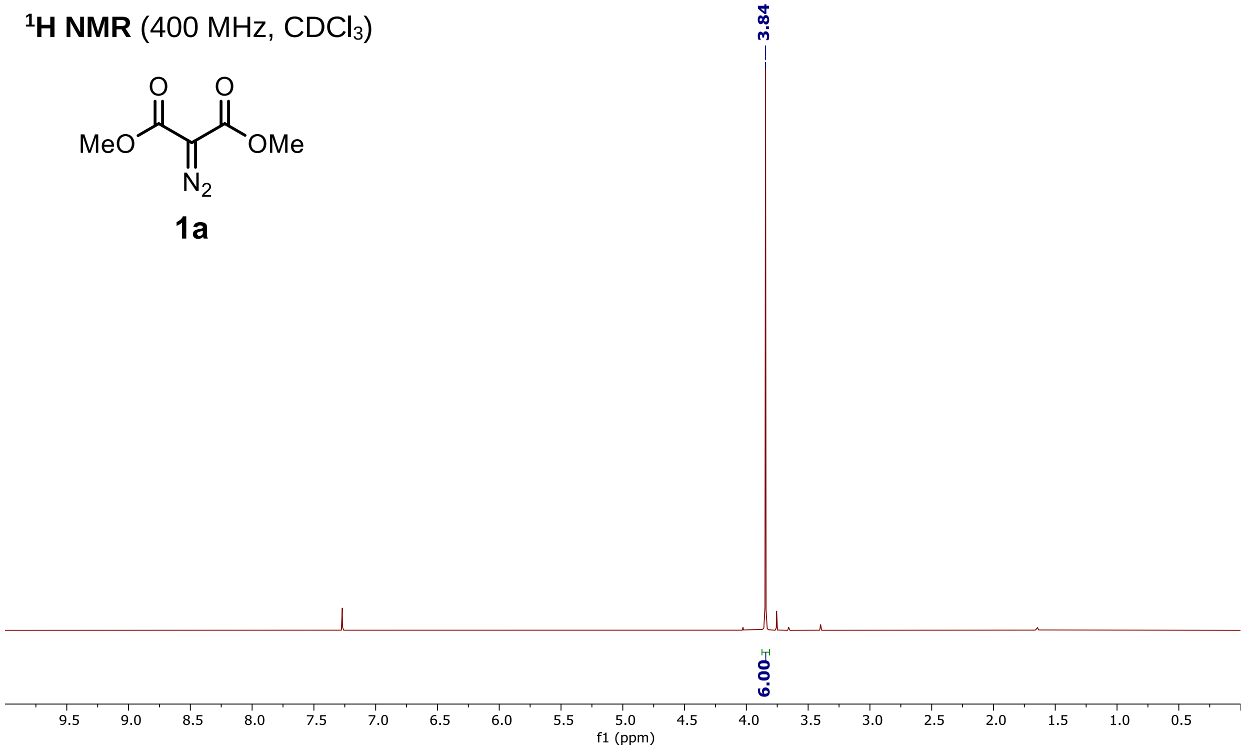

^{1}H NMR (400 MHz, $CDCl_3$)

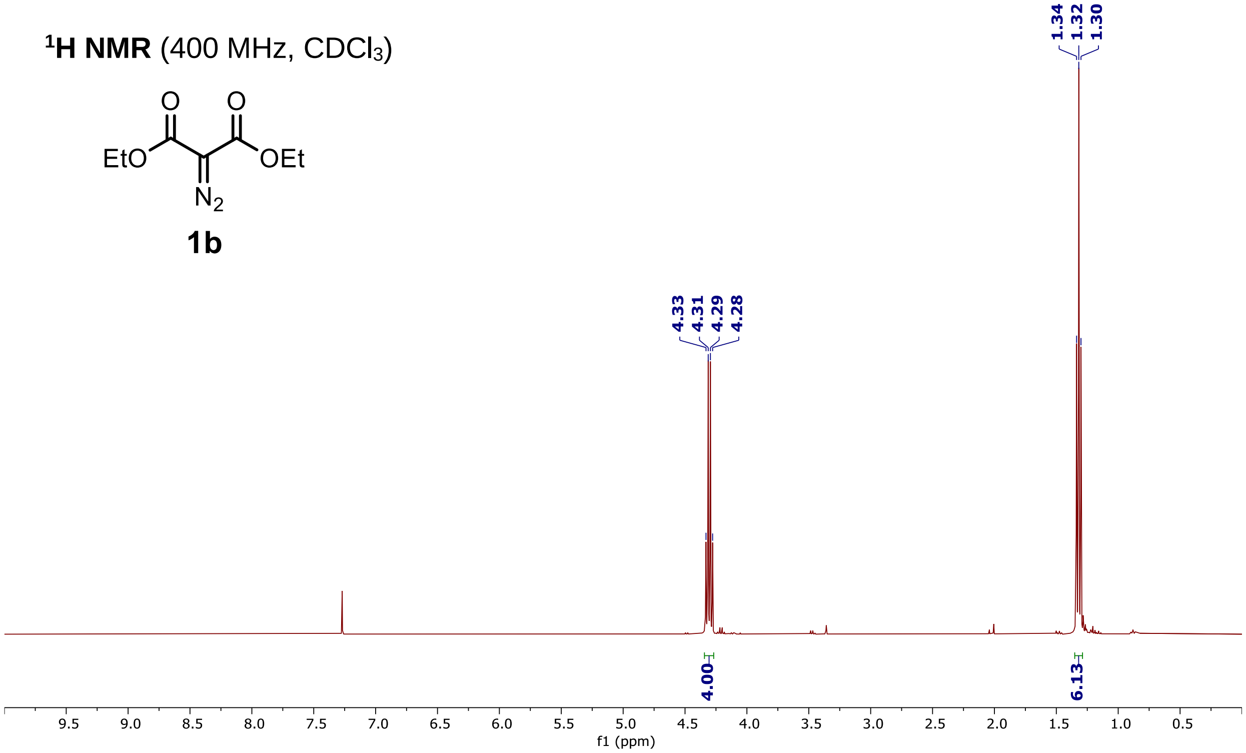

^{1}H NMR (400 MHz, $CDCl_3$)

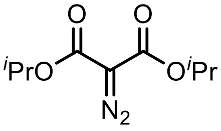

1c

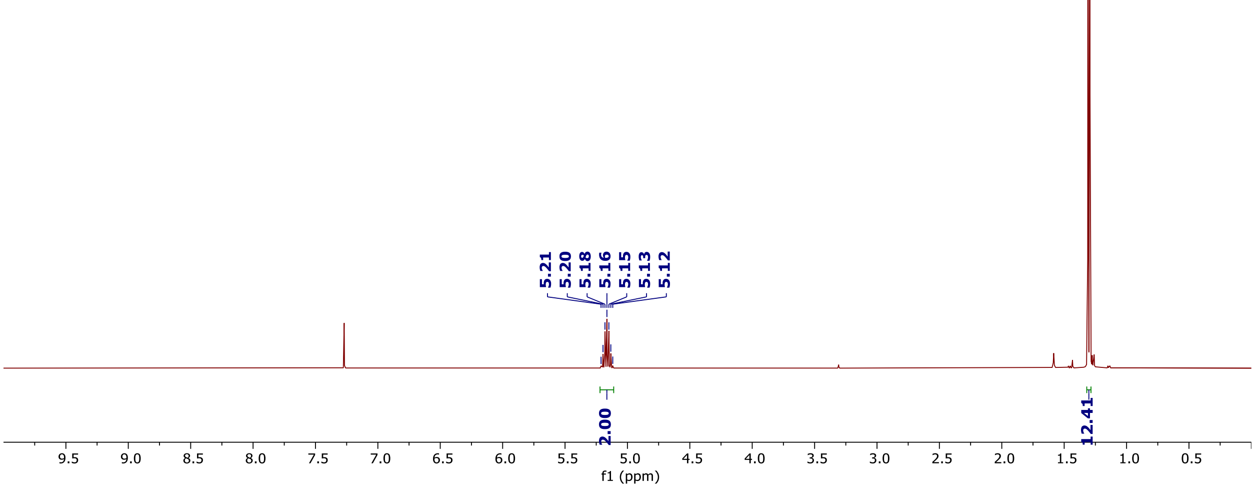

^{1}H NMR (400 MHz, $CDCl_3$)

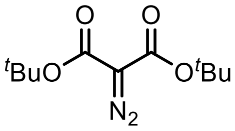

1d

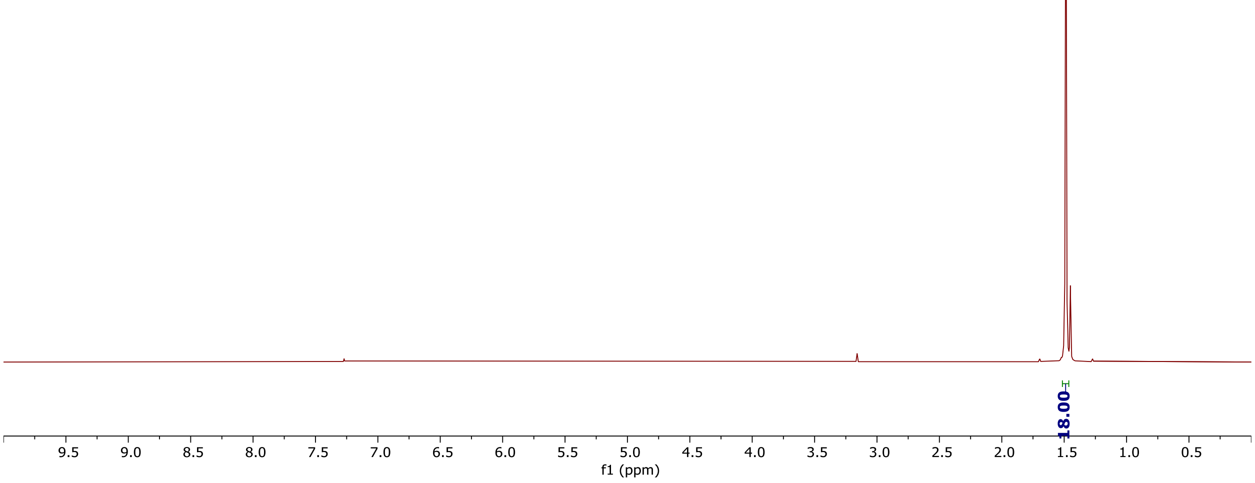

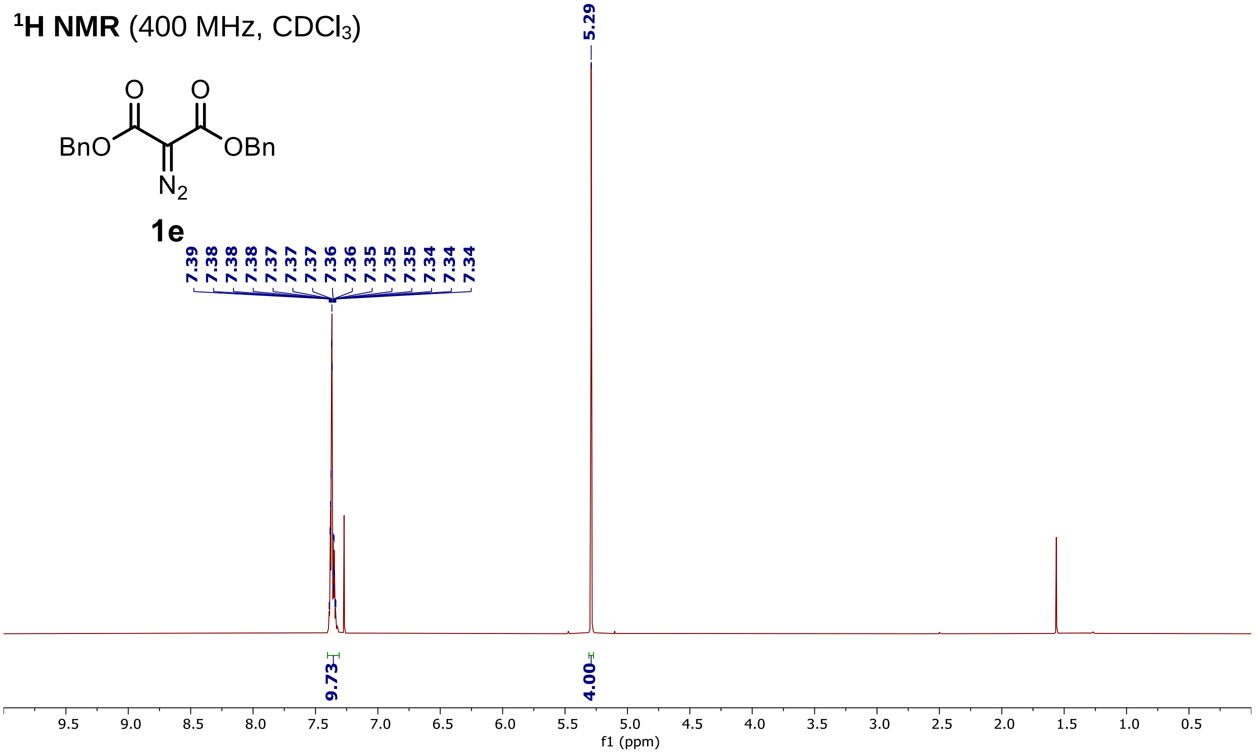

1H NMR (400 MHz, CDCl3)
BnO
OBn
O
O
N2
1e
7.39
7.38
7.38
7.38
7.37
7.37
7.37
7.36
7.36
7.35
7.35
7.35
7.34
7.34
7.34
5.29
9.73
4.00
9.5
9.0
8.5
8.0
7.5
7.0
6.5
6.0
5.5
5.0
4.5
4.0
3.5
3.0
2.5
2.0
1.5
1.0
0.5
f1 (ppm)

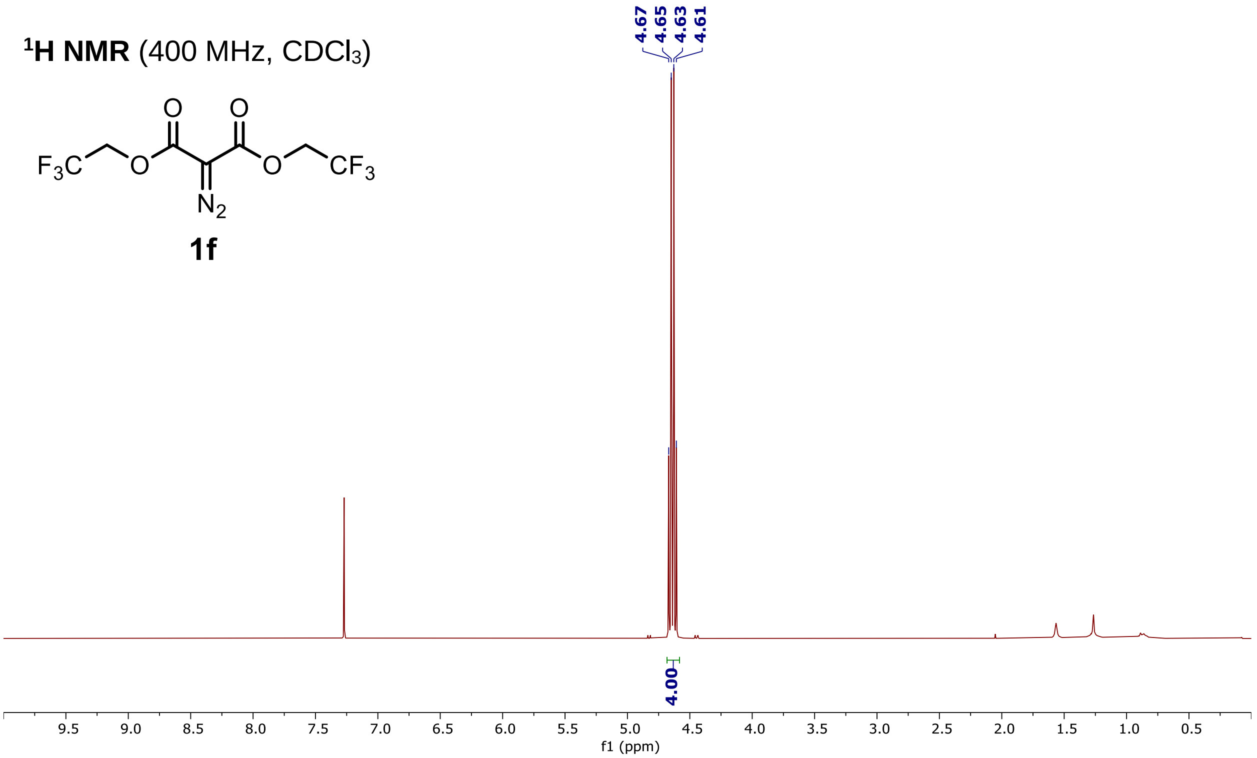

1H NMR (400 MHz, CDCl3)
F3C
CF3
O
O
O
O
N2
1f
4.67
4.65
4.63
4.61
4.00
9.5
9.0
8.5
8.0
7.5
7.0
6.5
6.0
5.5
5.0
4.5
4.0
3.5
3.0
2.5
2.0
1.5
1.0
0.5
f1 (ppm)

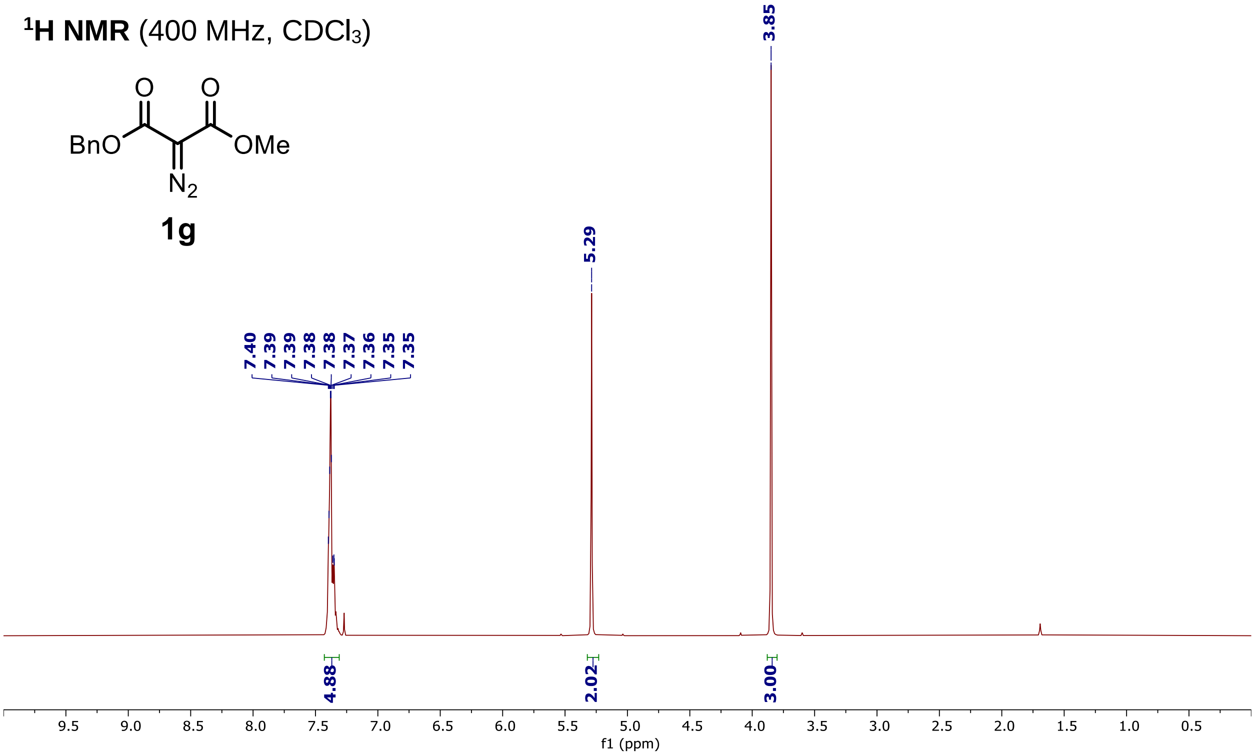
1H NMR (400 MHz, CDCl3)
BnO
OMe
O
O
N2
1g
3.85
5.29
7.40 7.39 7.39 7.38 7.38 7.37 7.36 7.35 7.35
4.88
2.02
3.00
9.5 9.0 8.5 8.0 7.5 7.0 6.5 6.0 5.5 5.0 4.5 4.0 3.5 3.0 2.5 2.0 1.5 1.0 0.5
f1 (ppm)

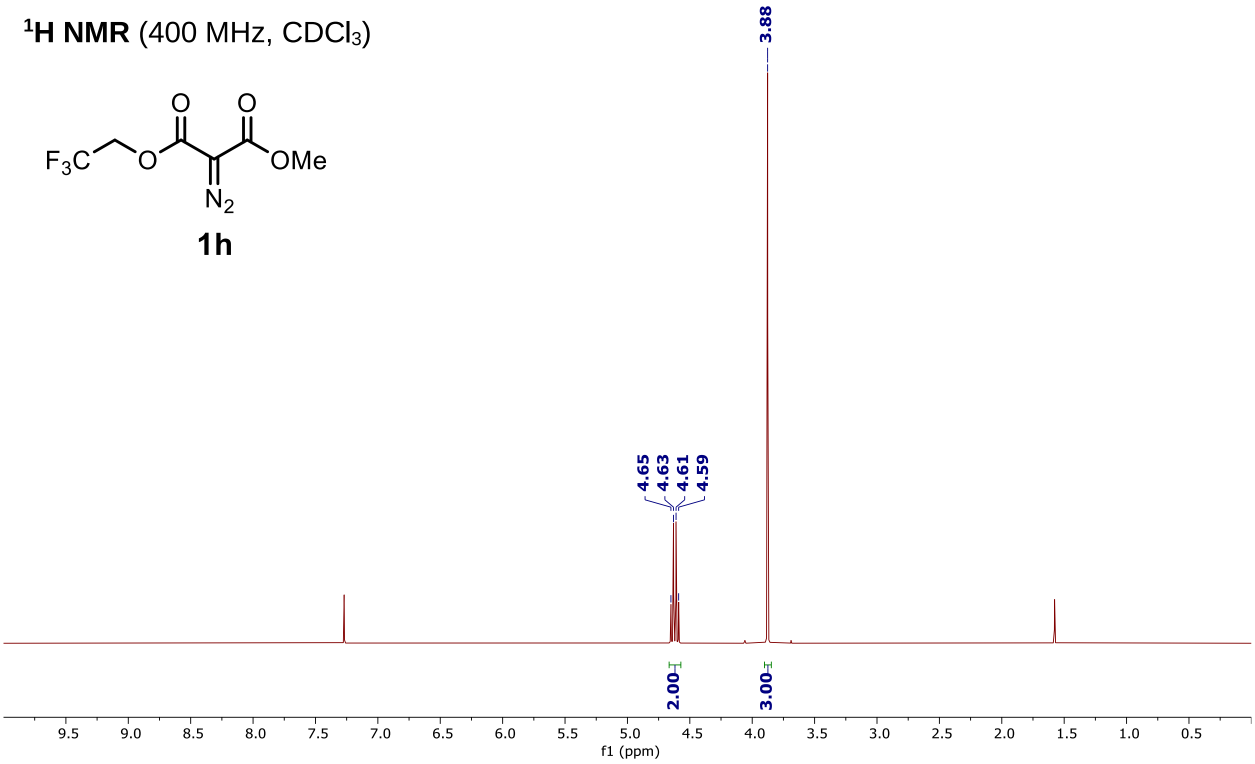
1H NMR (400 MHz, CDCl3)
F3C
O
OMe
O
O
N2
1h
3.88
4.65 4.63 4.61 4.59
2.00
3.00
9.5 9.0 8.5 8.0 7.5 7.0 6.5 6.0 5.5 5.0 4.5 4.0 3.5 3.0 2.5 2.0 1.5 1.0 0.5
f1 (ppm)

^{1}H NMR (400 MHz, $CDCl_3$)

1i

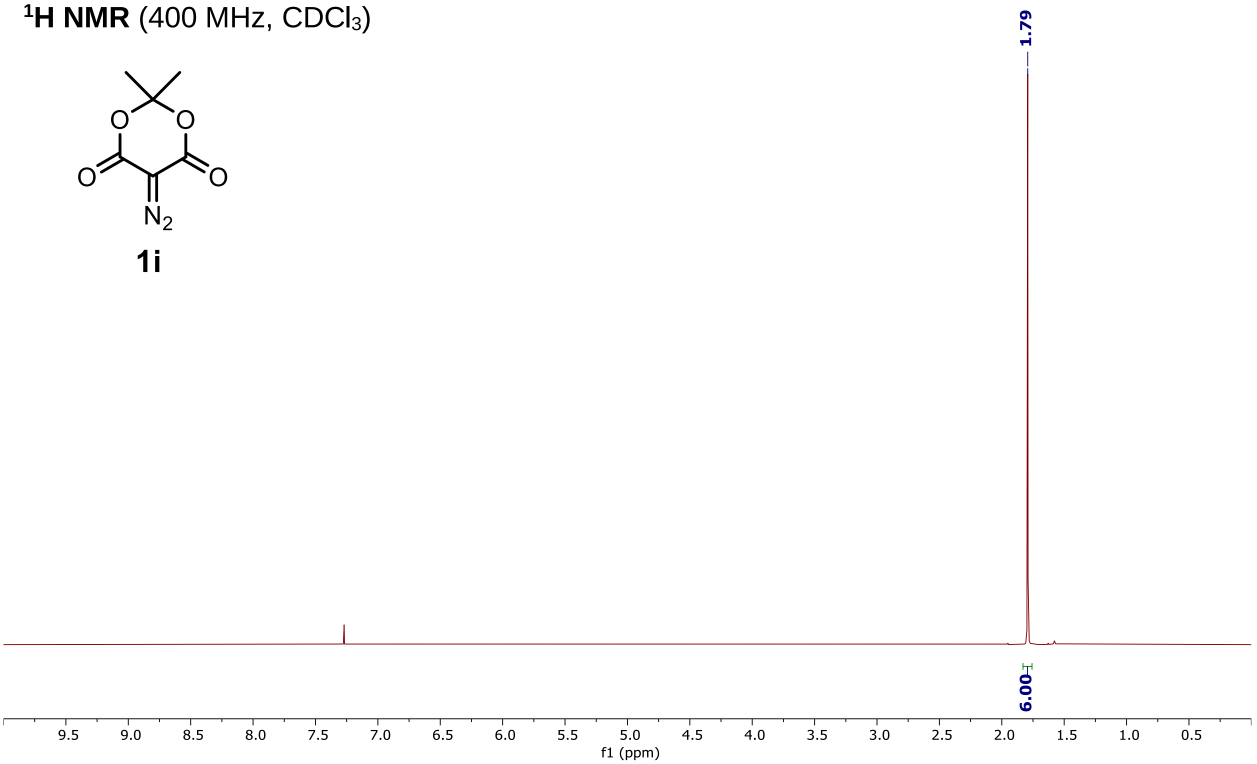

^{1}H NMR (500 MHz, $CDCl_3$)

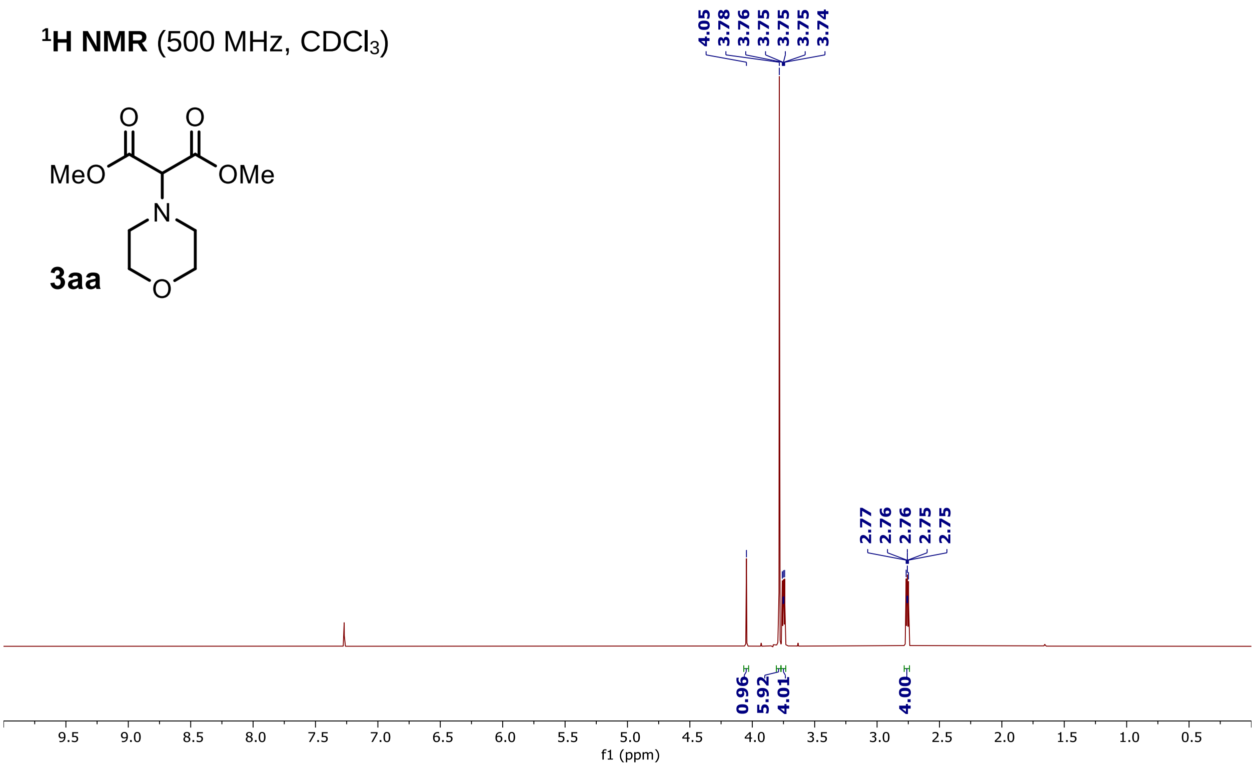

^{13}C NMR (126 MHz, $CDCl_3$)

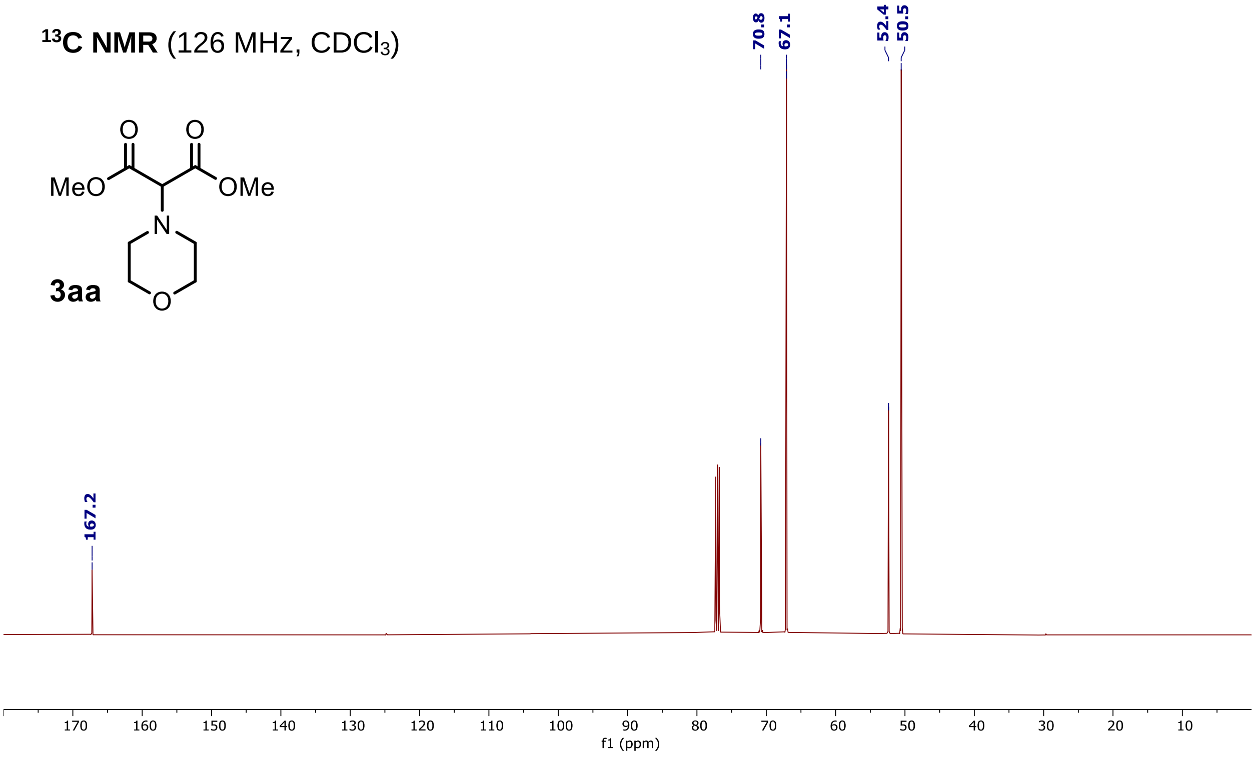

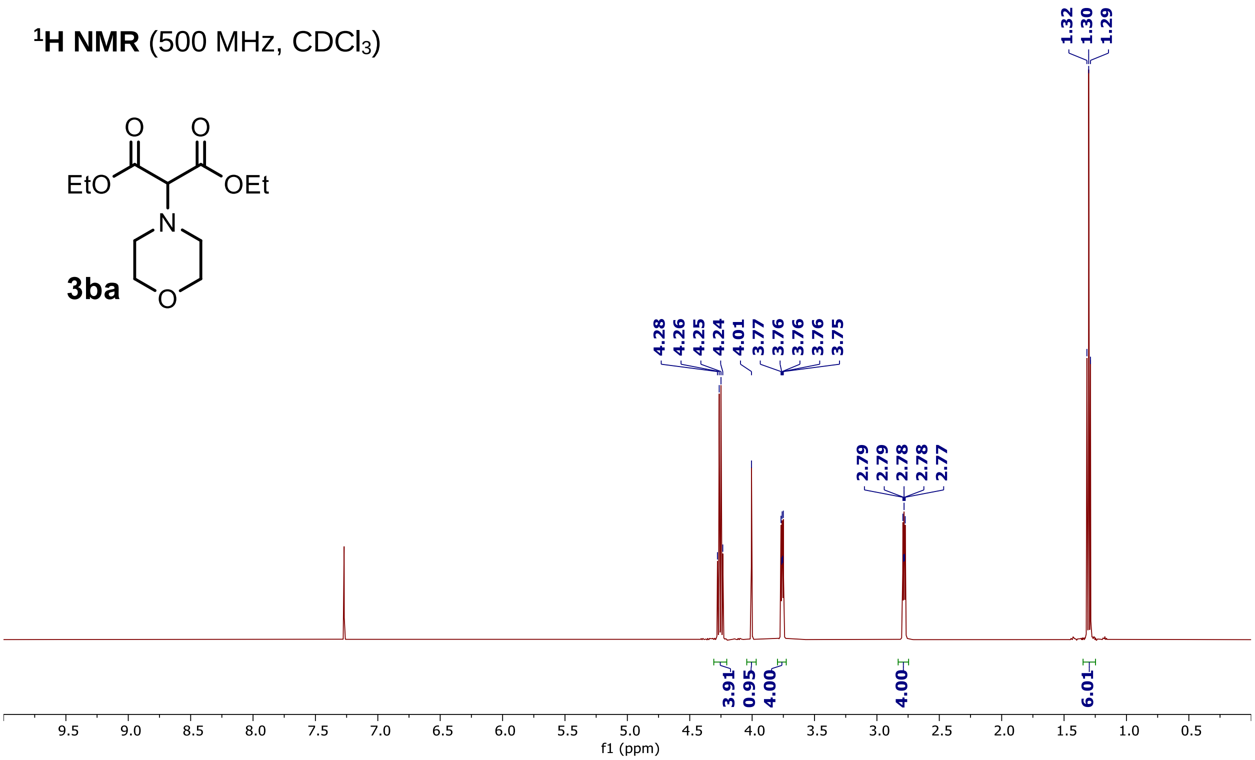
1H NMR (500 MHz, CDCl3)
EtO
OEt
O
O
N
O
3ba
4.28
4.26
4.25
4.24
4.01
3.77
3.76
3.76
3.76
3.75
2.79
2.79
2.78
2.78
2.77
1.32
1.30
1.29
3.91
0.95
4.00
4.00
6.01
f1 (ppm)

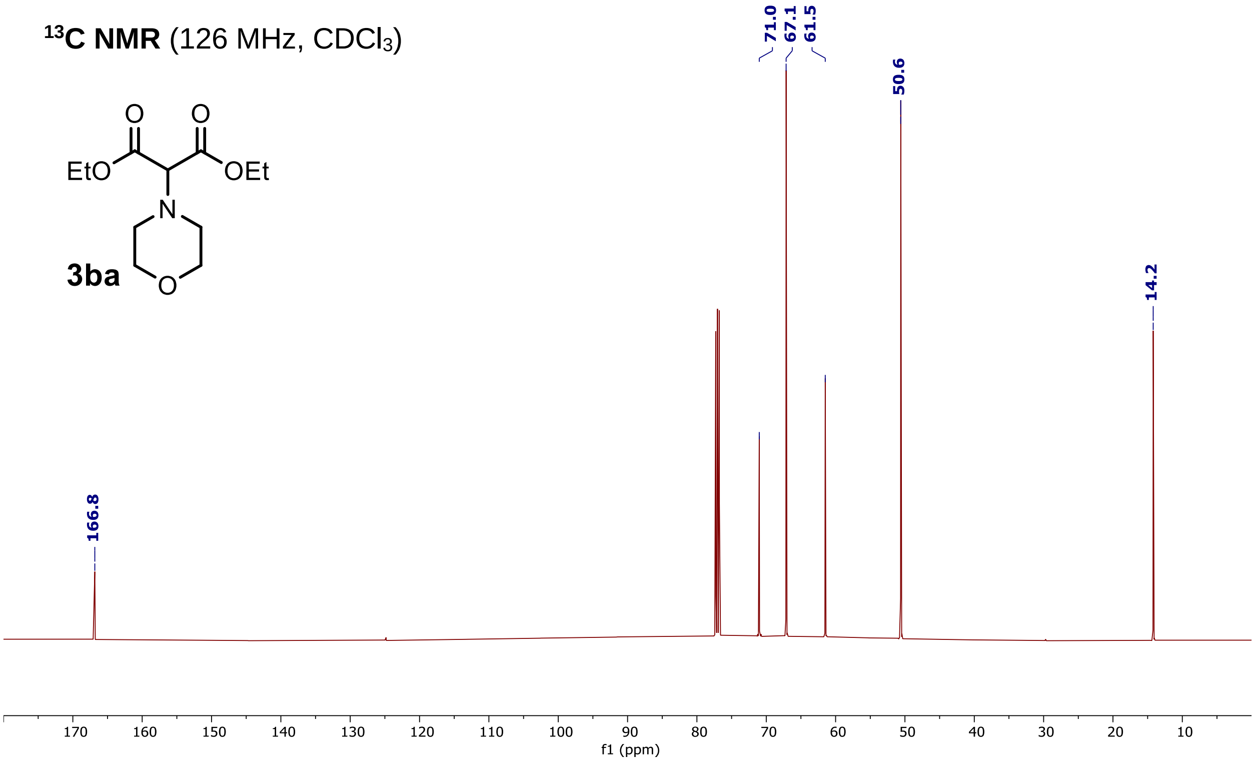
13C NMR (126 MHz, CDCl3)
EtO
OEt
O
O
N
O
3ba
166.8
71.0
67.1
61.5
50.6
14.2
f1 (ppm)

^{1}H NMR (500 MHz, $CDCl_3$)

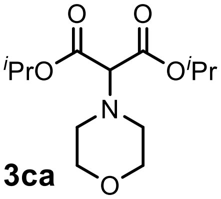

3ca

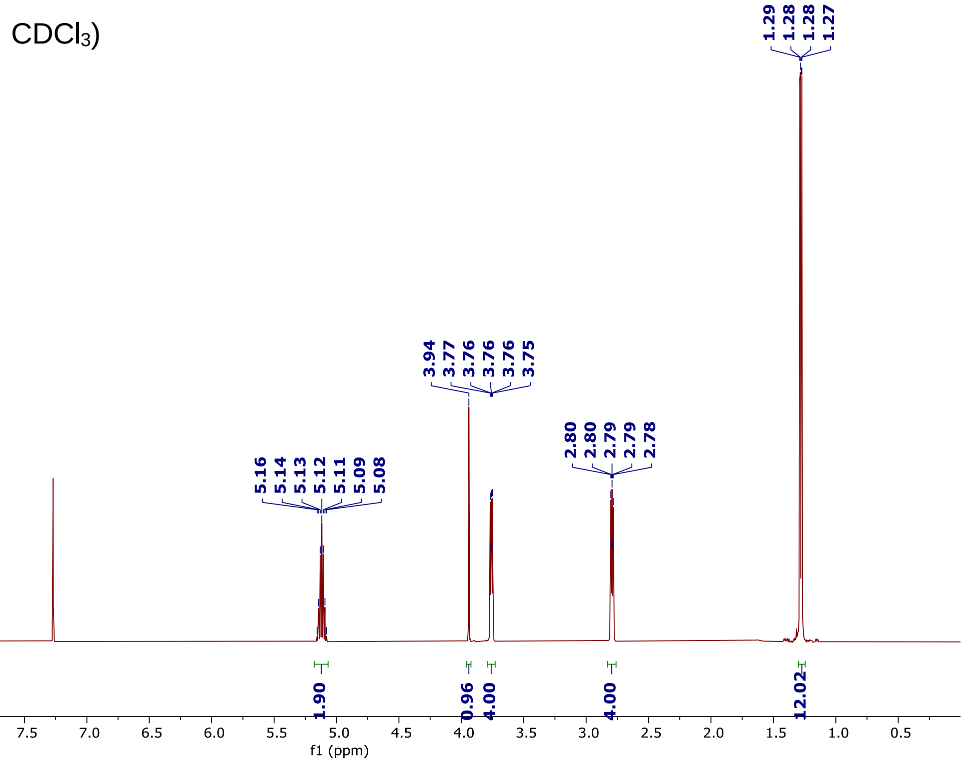

^{13}C NMR (126 MHz, $CDCl_3$)

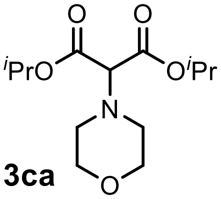

3ca

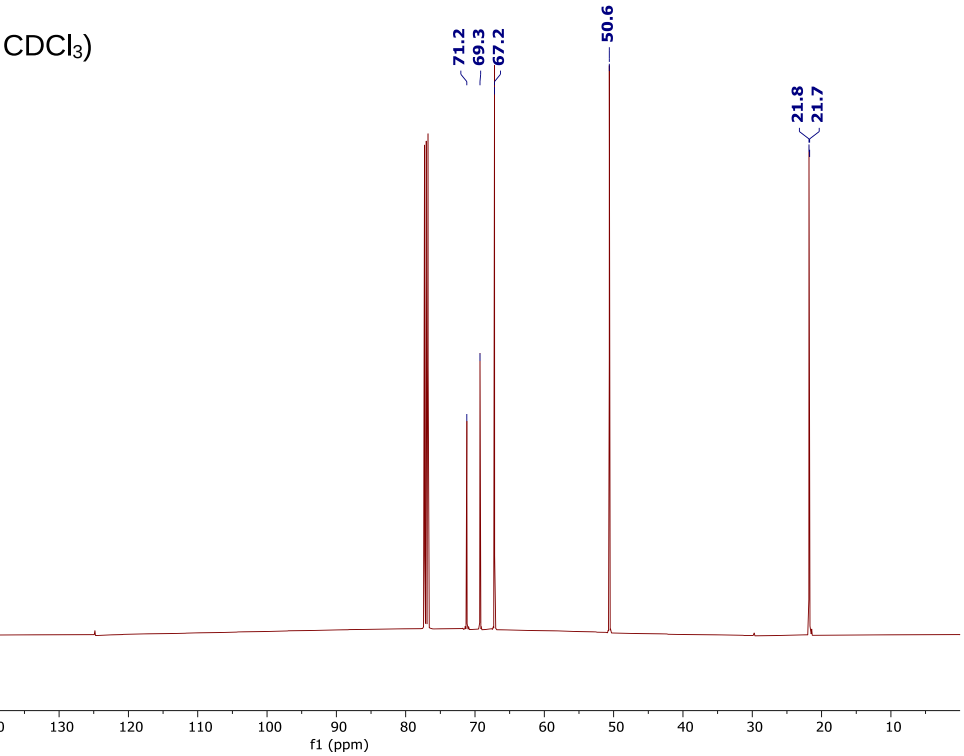

^{1}H NMR (500 MHz, $CDCl_3$)

3da

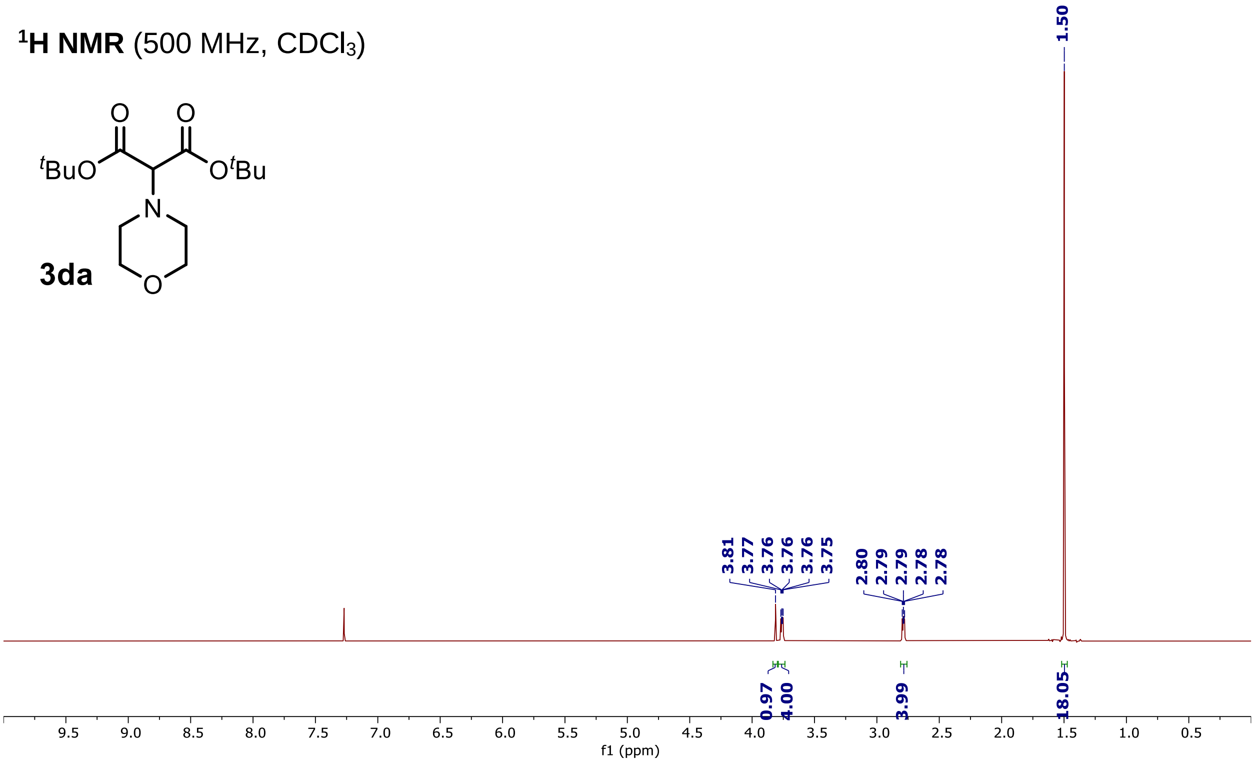

^{13}C NMR (126 MHz, $CDCl_3$)

3da

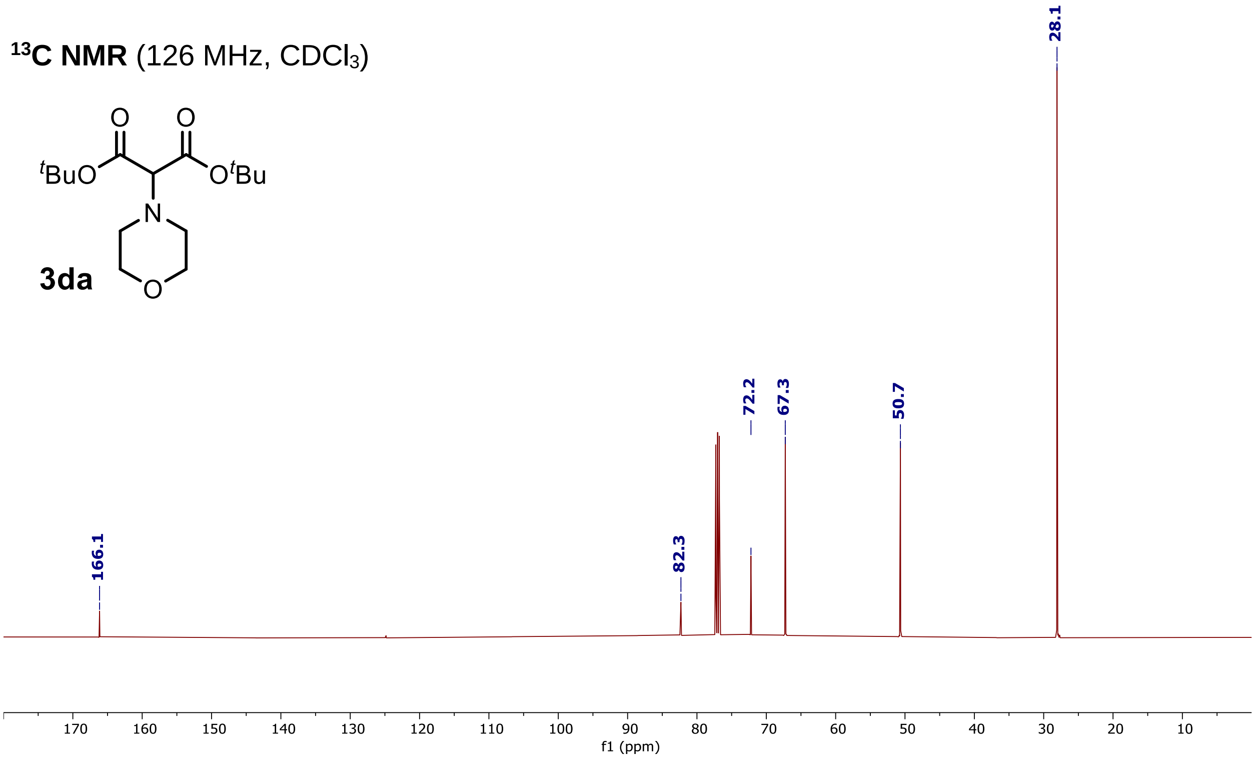

^{1}**H NMR** (500 MHz, $CDCl_3$)

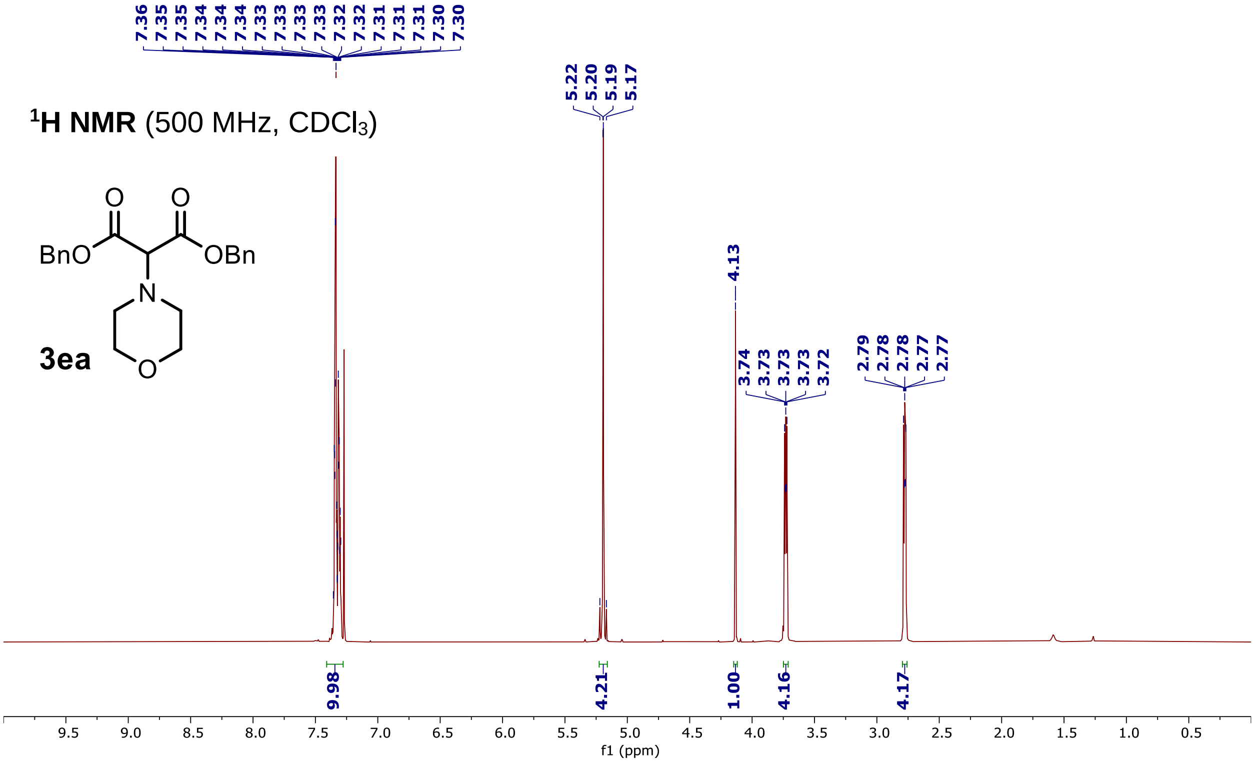

^{13}**C NMR** (126 MHz, $CDCl_3$)

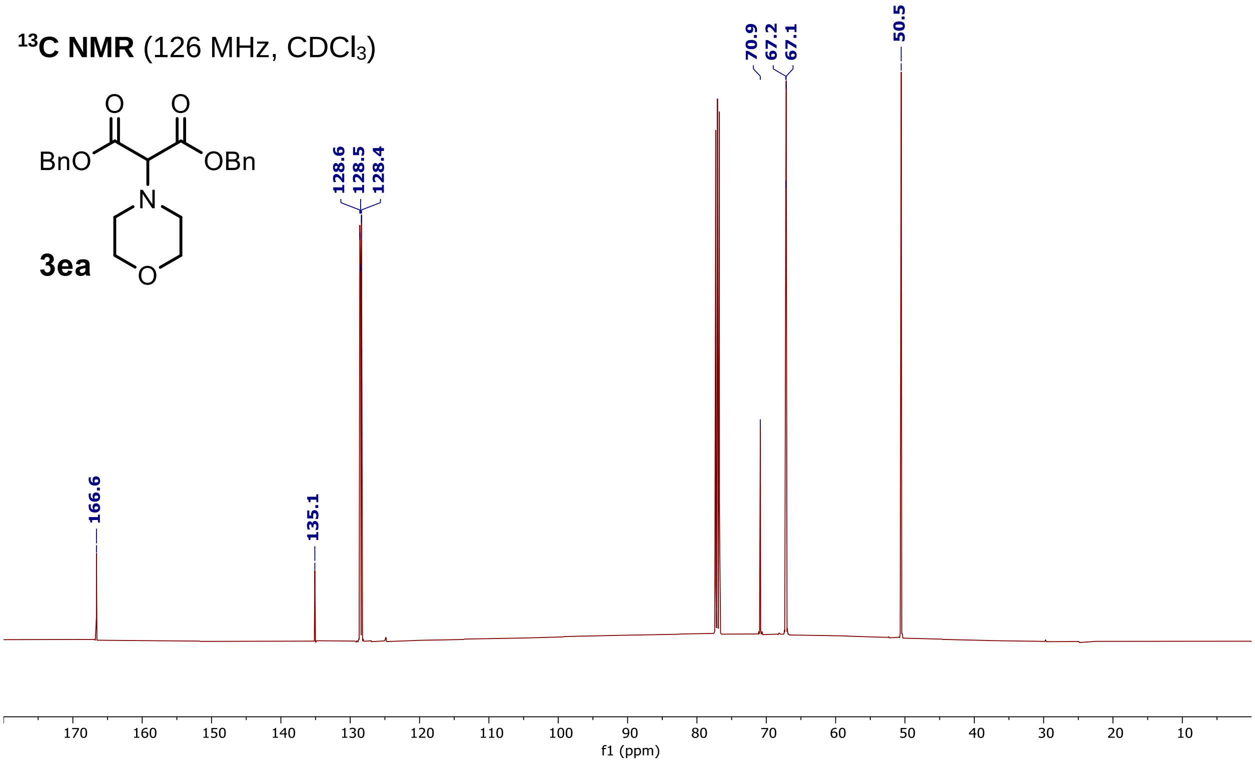

^{1}H NMR (500 MHz, $CDCl_3$)

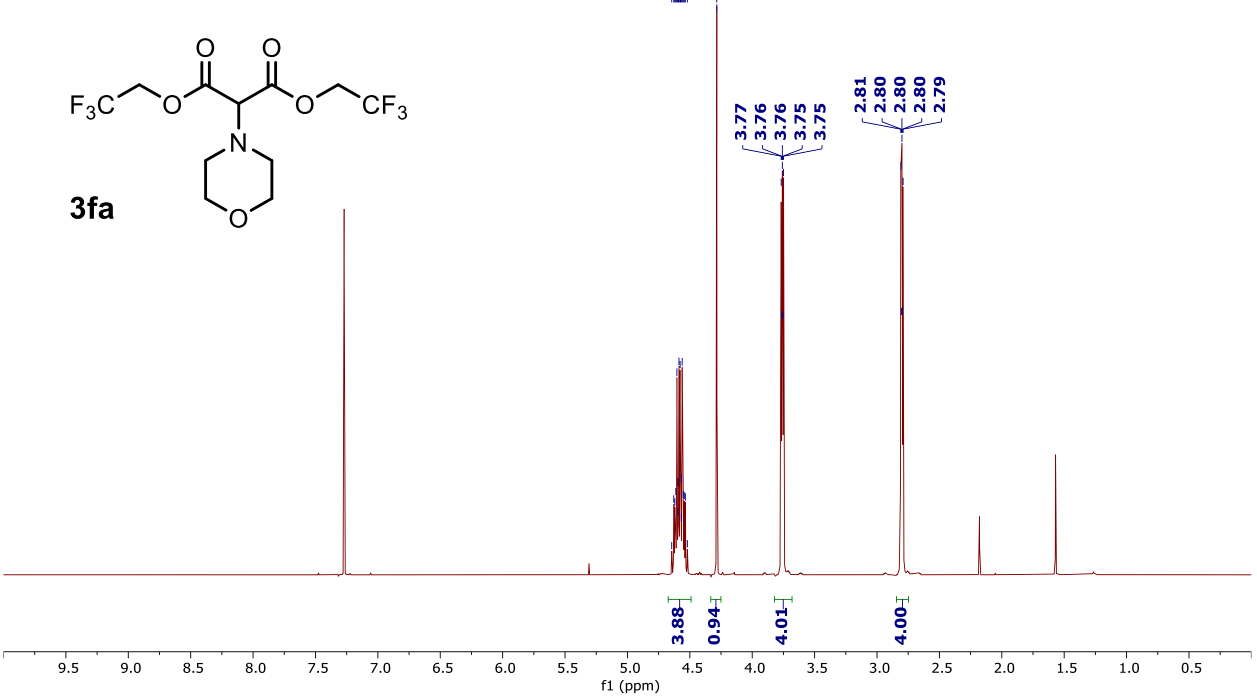

^{13}C NMR (126 MHz, $CDCl_3$)

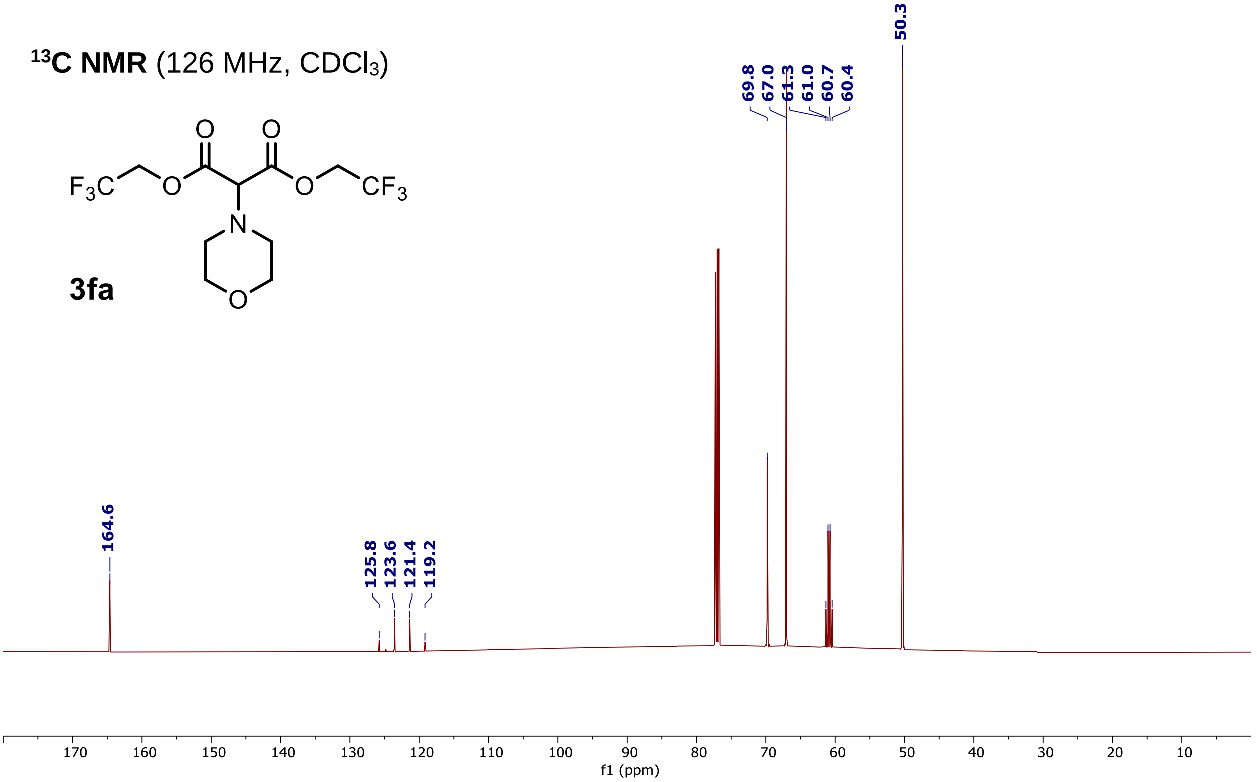

^{19}F NMR (282 MHz, $CDCl_3$)

3fa

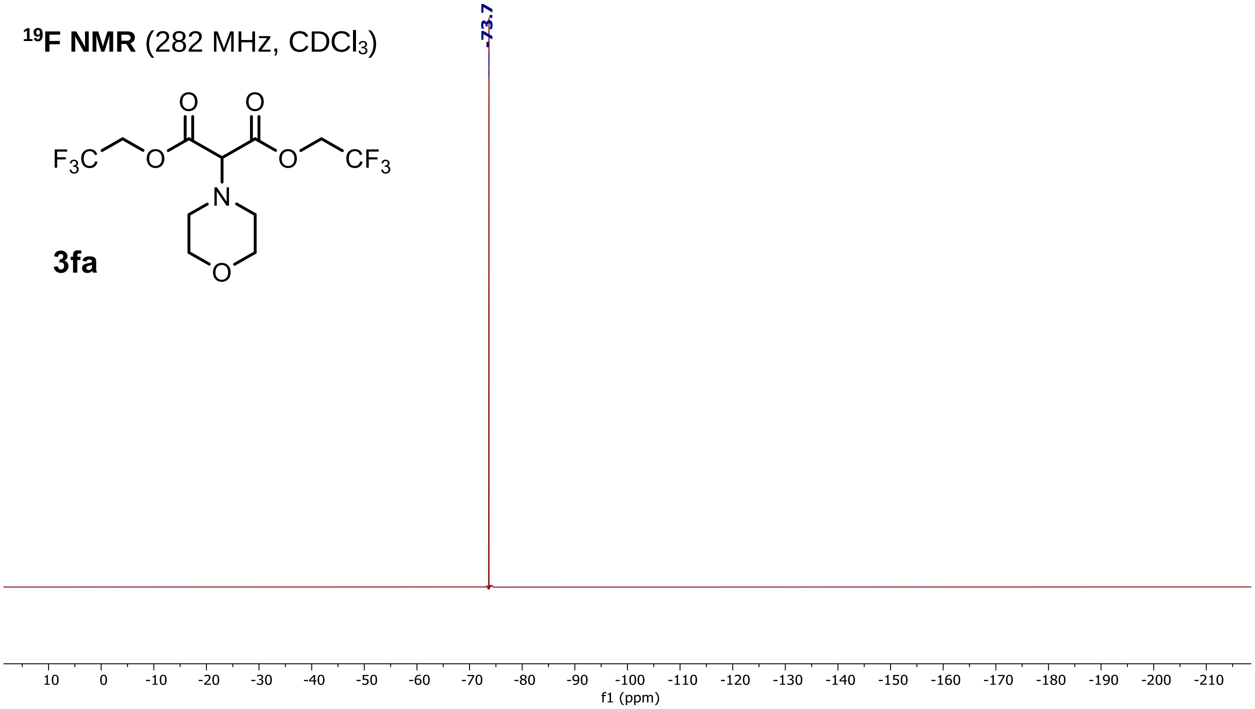

^{1}H NMR (500 MHz, $CDCl_3$)

3ga

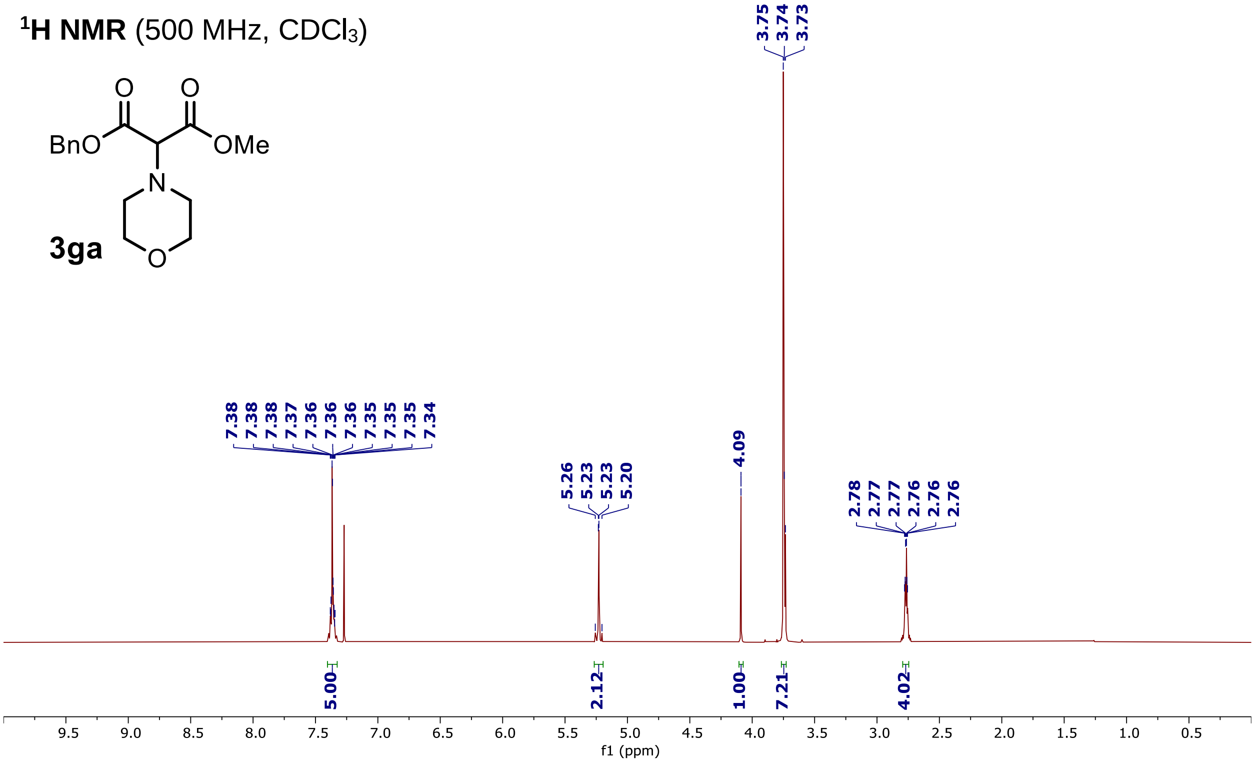

^{13}C NMR (126 MHz, $CDCl_3$)

3ga

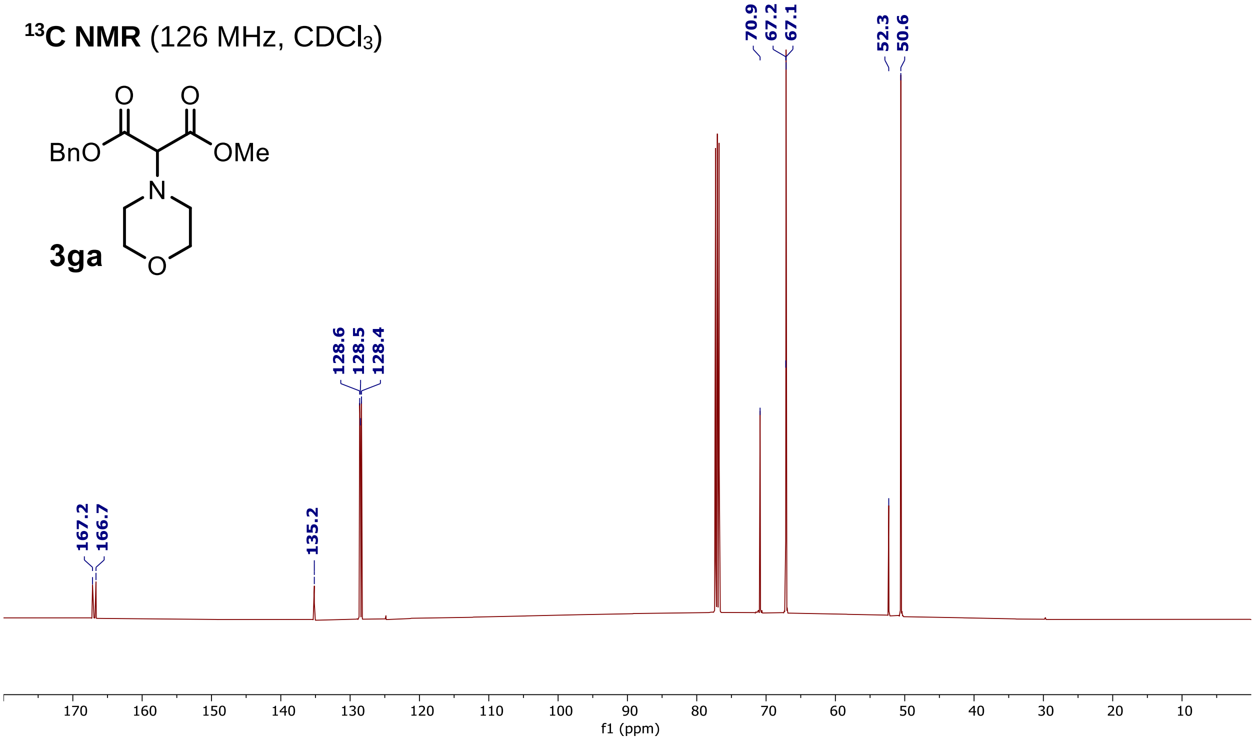

^{1}H NMR (500 MHz, $CDCl_3$)

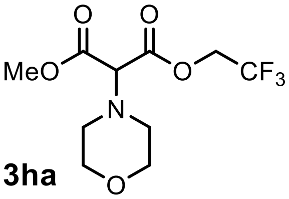

3ha

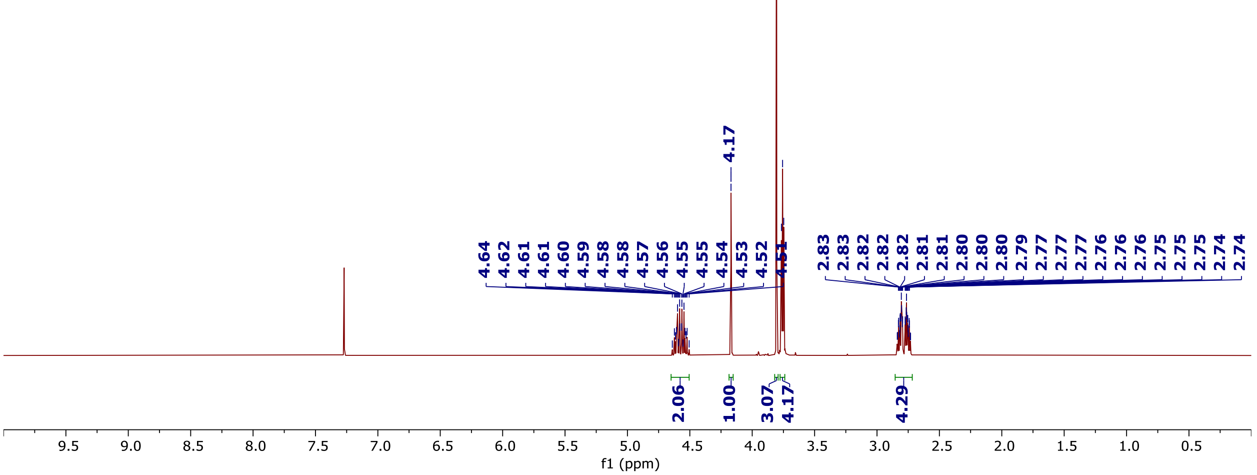

^{13}C NMR (126 MHz, $CDCl_3$)

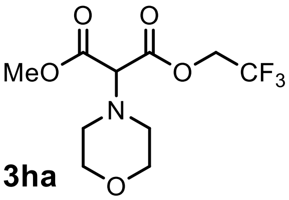

3ha

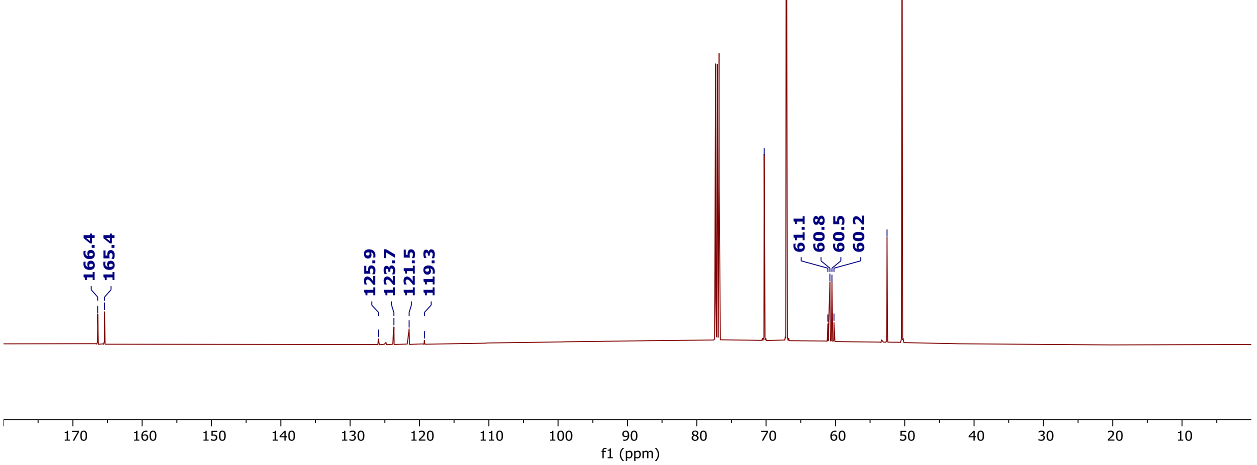

^{19}F NMR (282 MHz, $CDCl_3$)

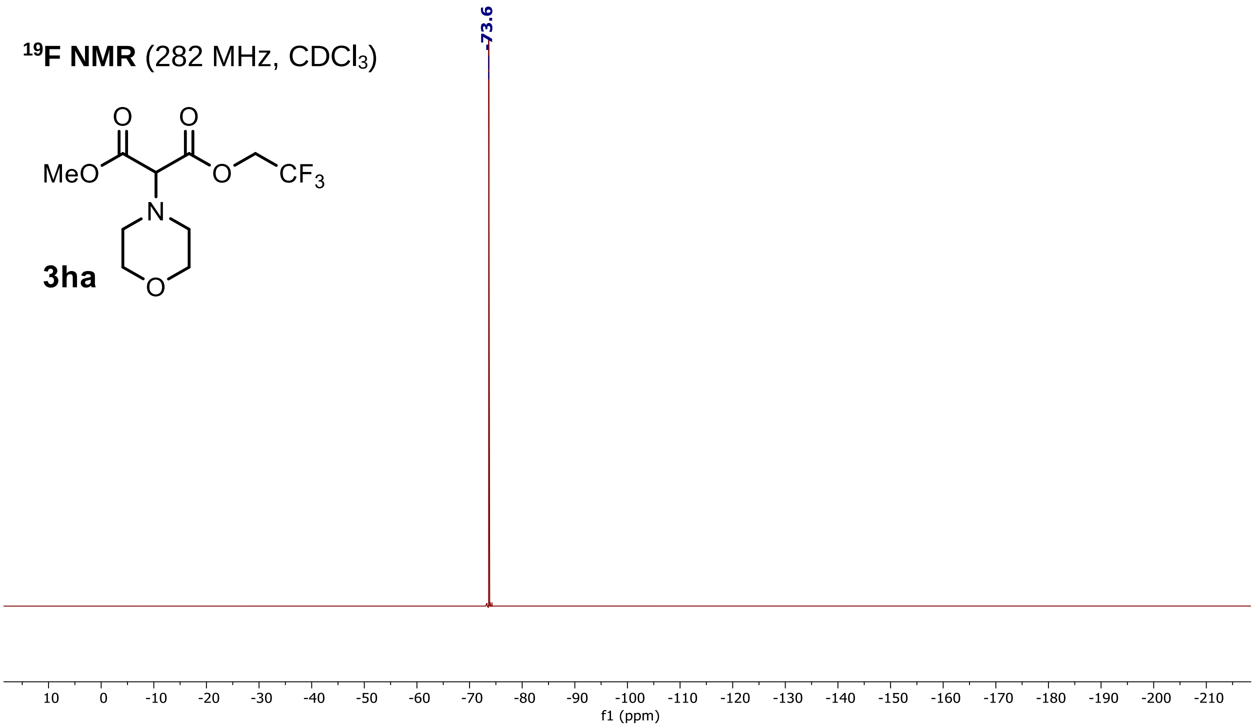

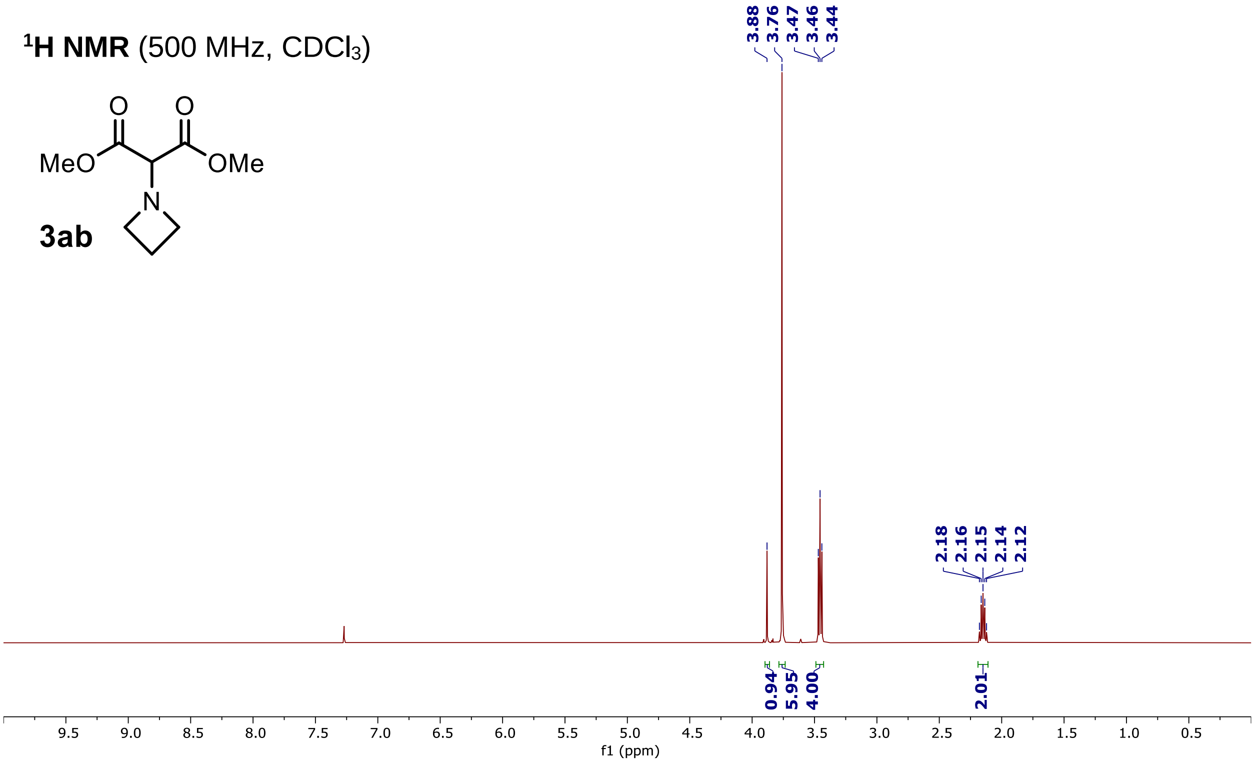
1H NMR (500 MHz, CDCl3)
MeO
OMe
O
O
N
3ab
3.88
3.76
3.47
3.46
3.44
2.18
2.16
2.15
2.14
2.12
0.94
5.95
4.00
2.01
f1 (ppm)

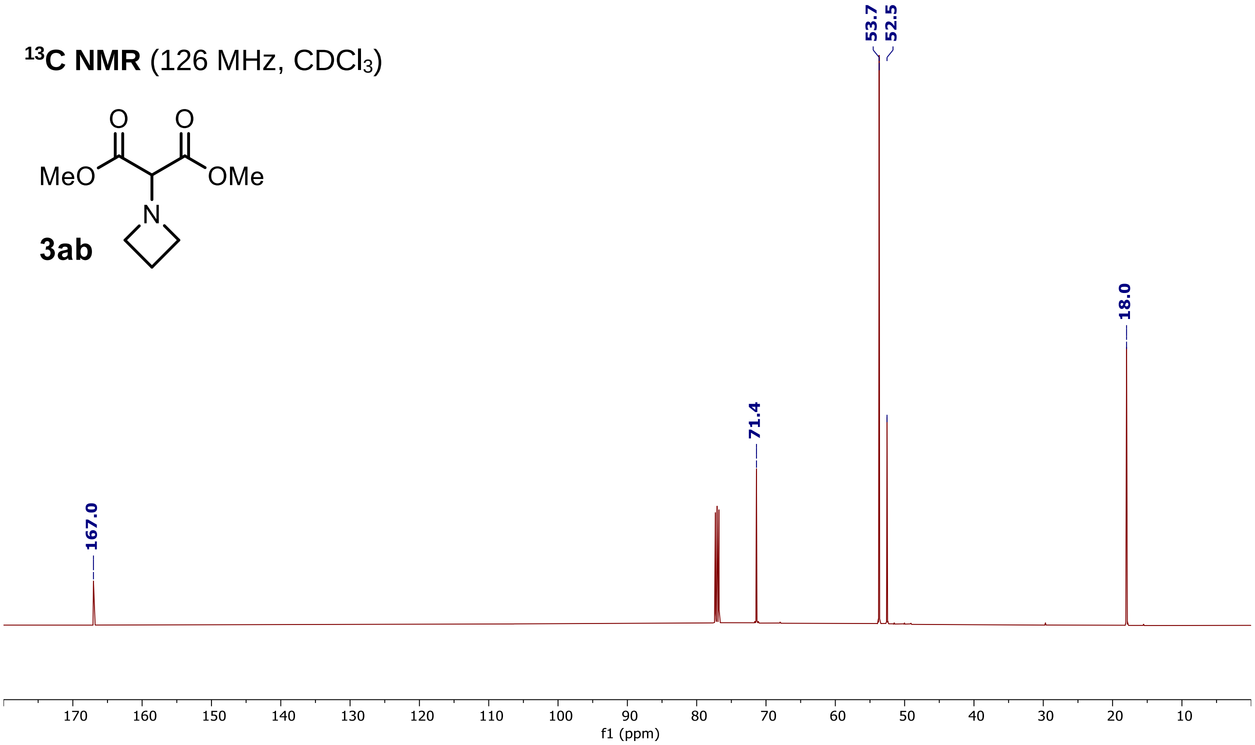
13C NMR (126 MHz, CDCl3)
MeO
OMe
O
O
N
3ab
167.0
71.4
53.7
52.5
18.0
f1 (ppm)

^{1}H NMR (500 MHz, $CDCl_3$)

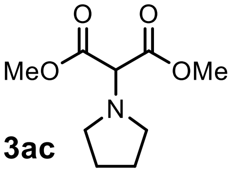

3ac

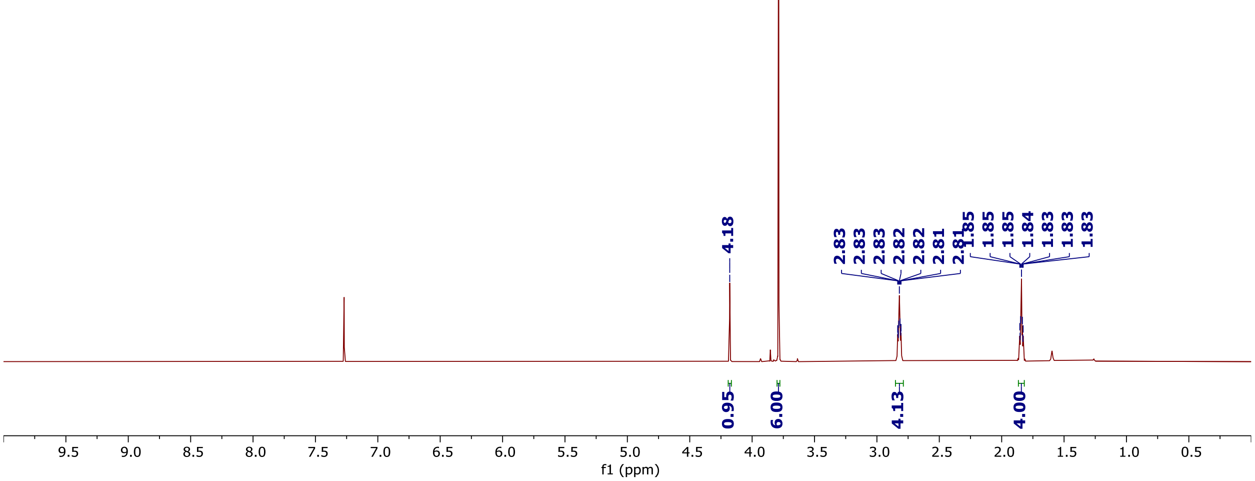

^{13}C NMR (126 MHz, $CDCl_3$)

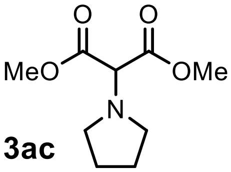

3ac

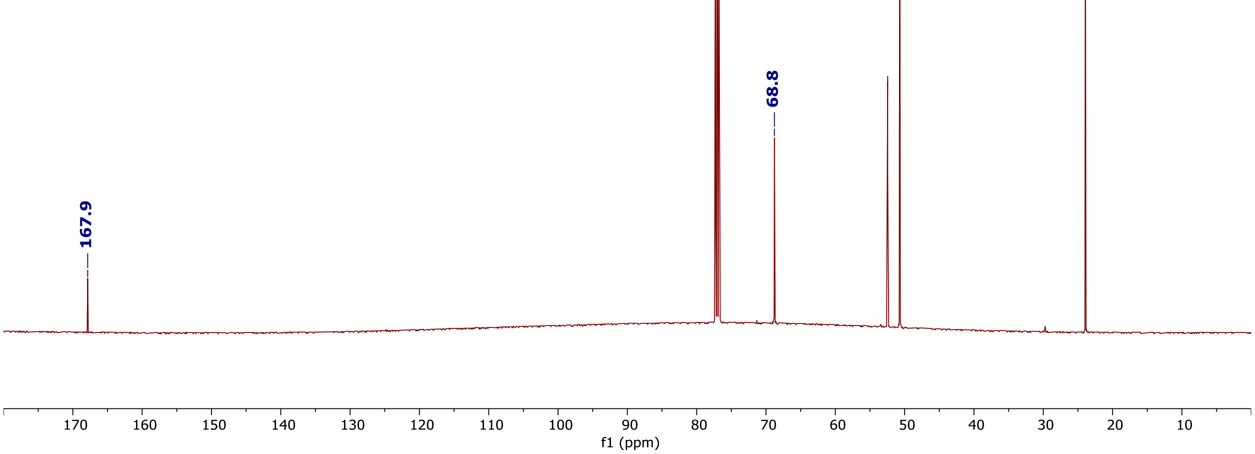

^{1}H NMR (500 MHz, $CDCl_3$)

3ad

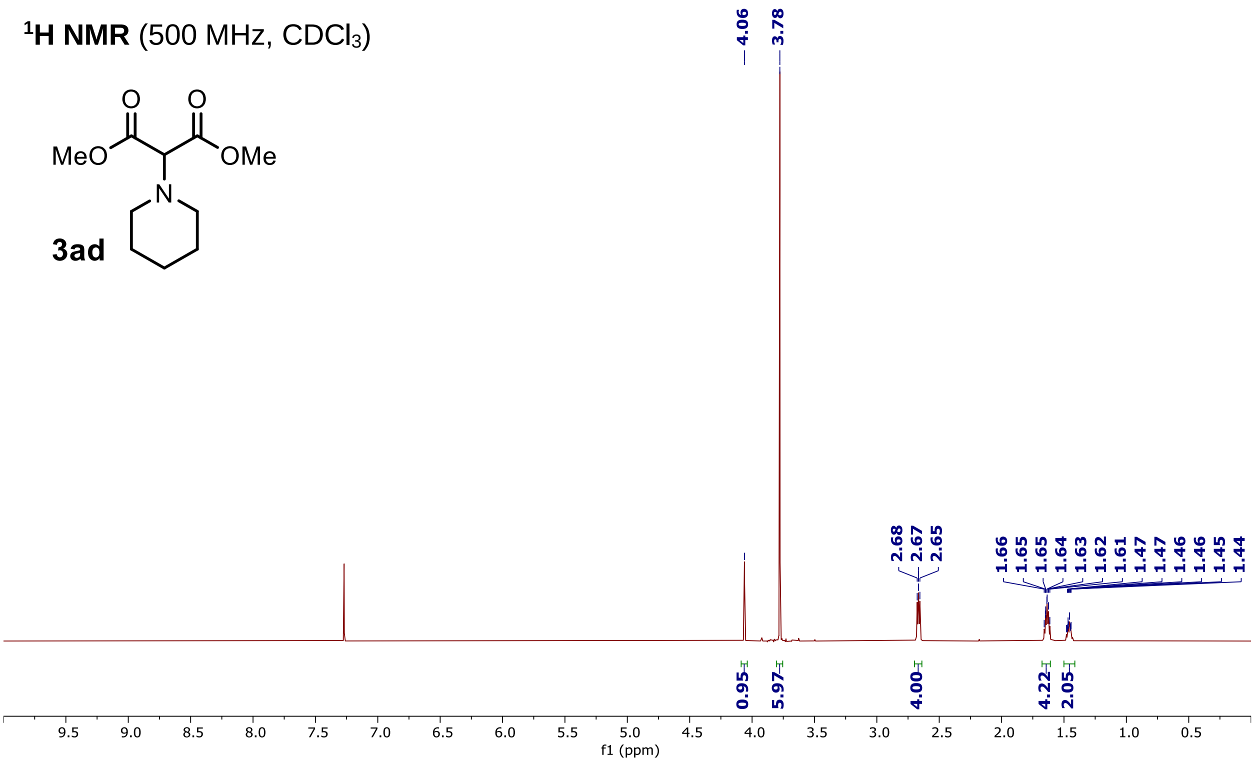

^{13}C NMR (126 MHz, $CDCl_3$)

3ad

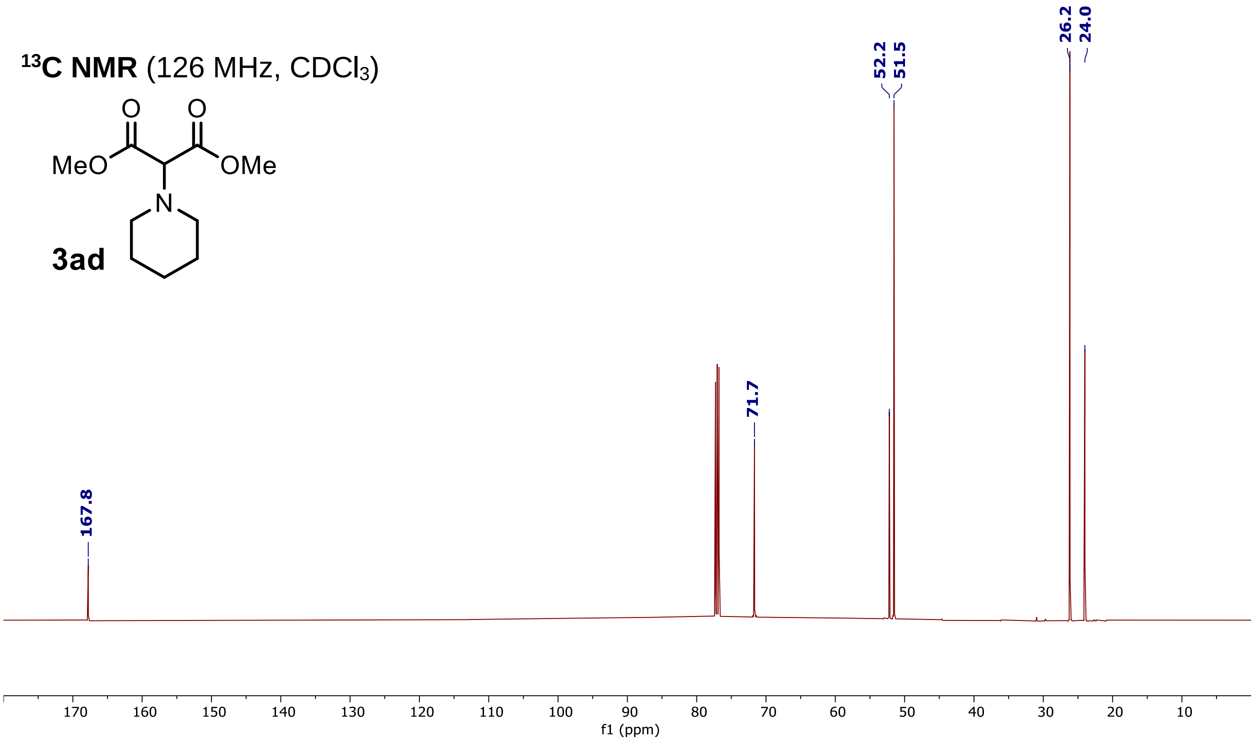

^{1}H NMR (500 MHz, $CDCl_3$)

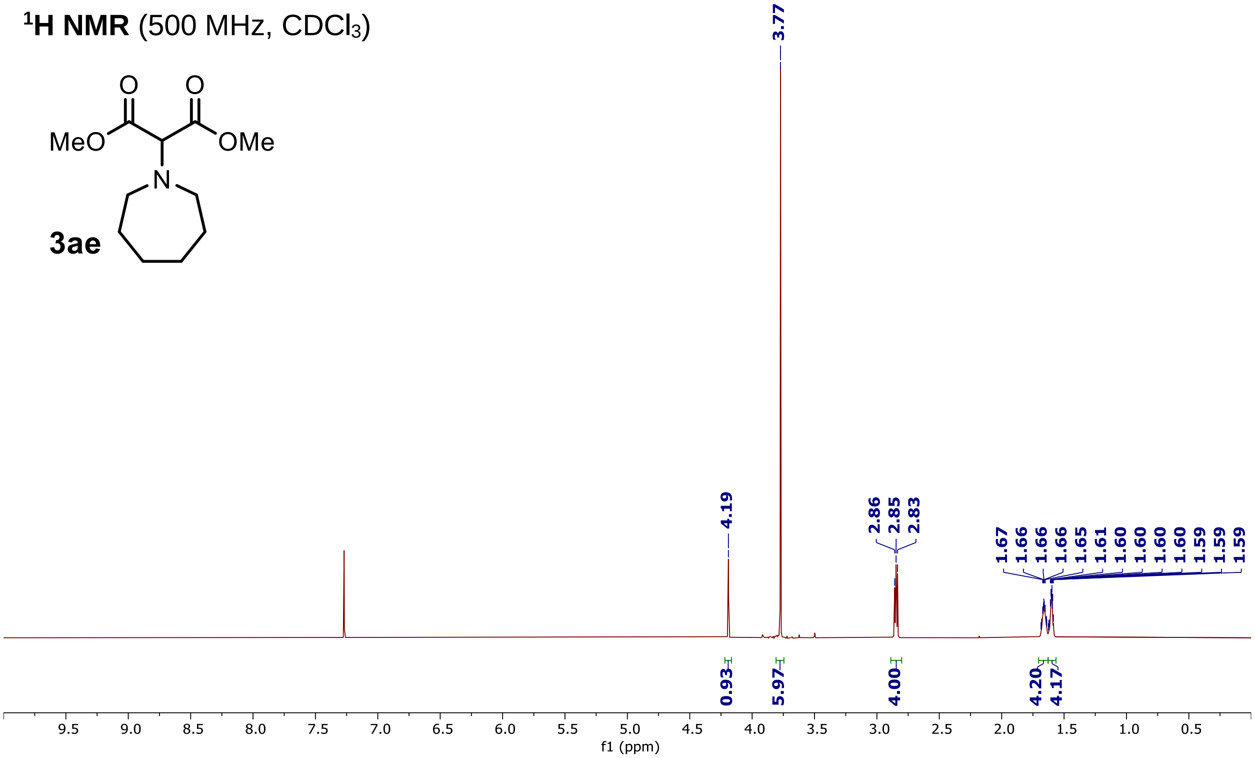

^{13}C NMR (126 MHz, $CDCl_3$)

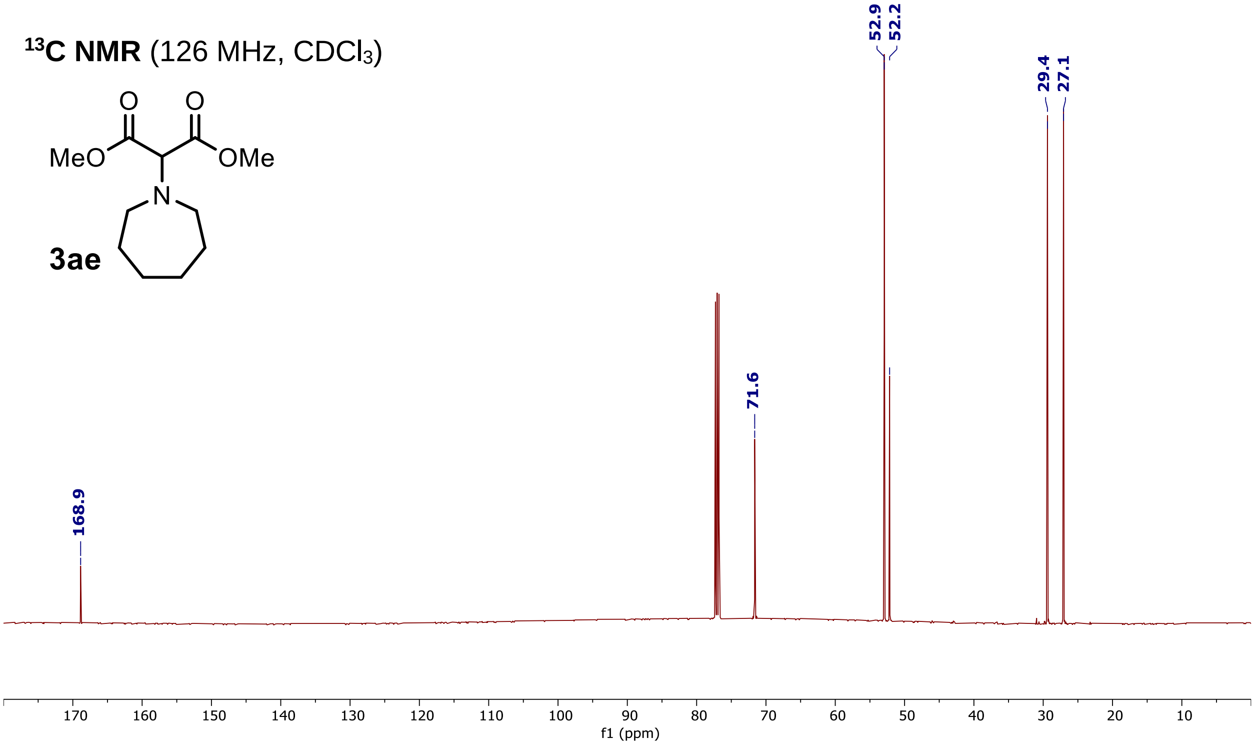

^{1}H NMR (500 MHz, $CDCl_3$)

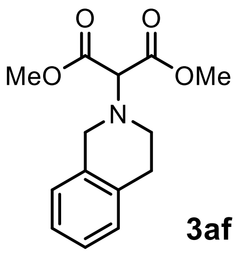

3af

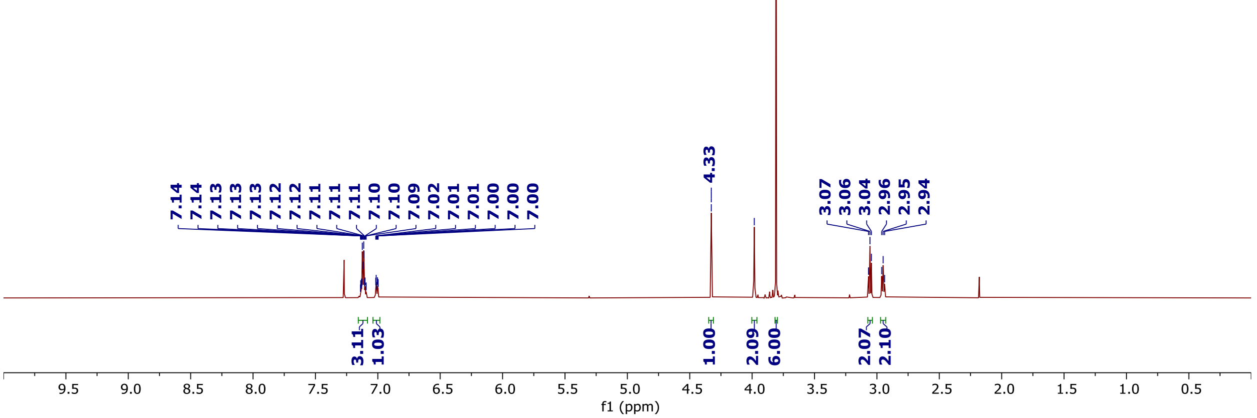

^{13}C NMR (126 MHz, $CDCl_3$)

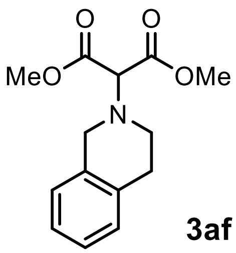

3af

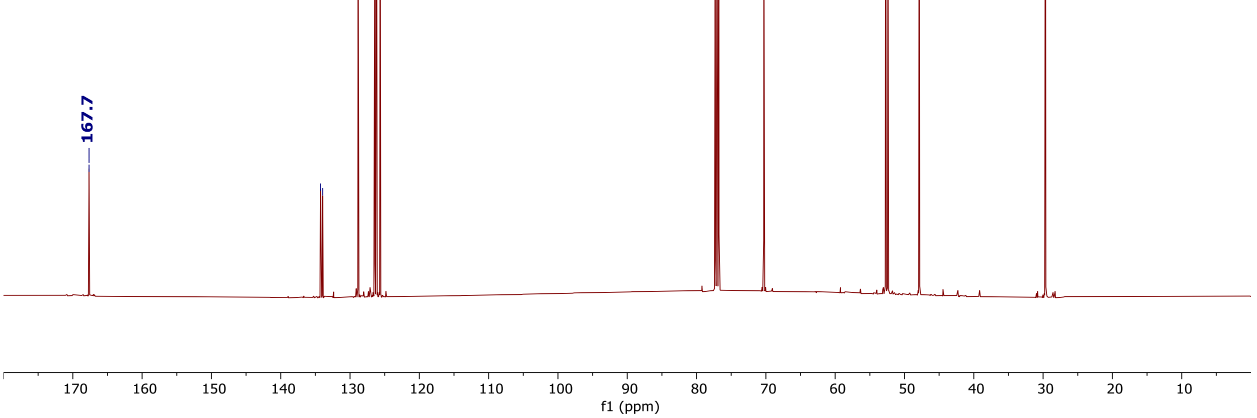

^{1}H NMR (500 MHz, $CDCl_3$)

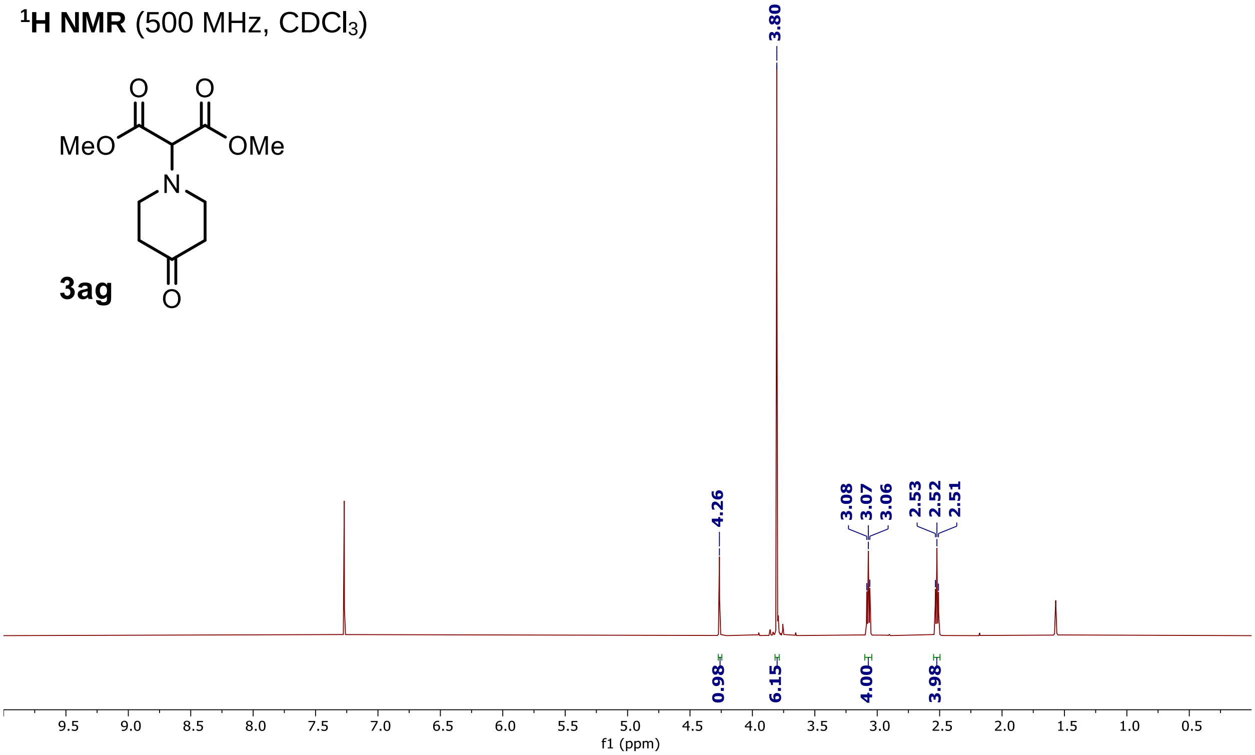

^{13}C NMR (126 MHz, $CDCl_3$)

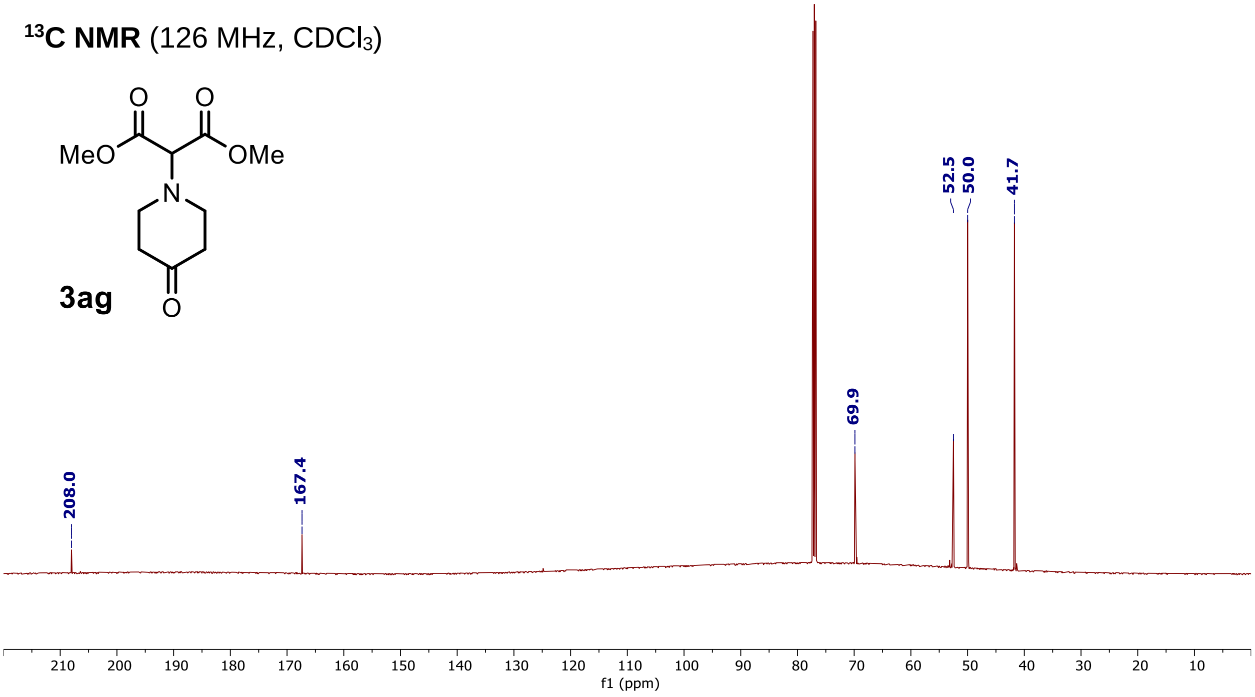

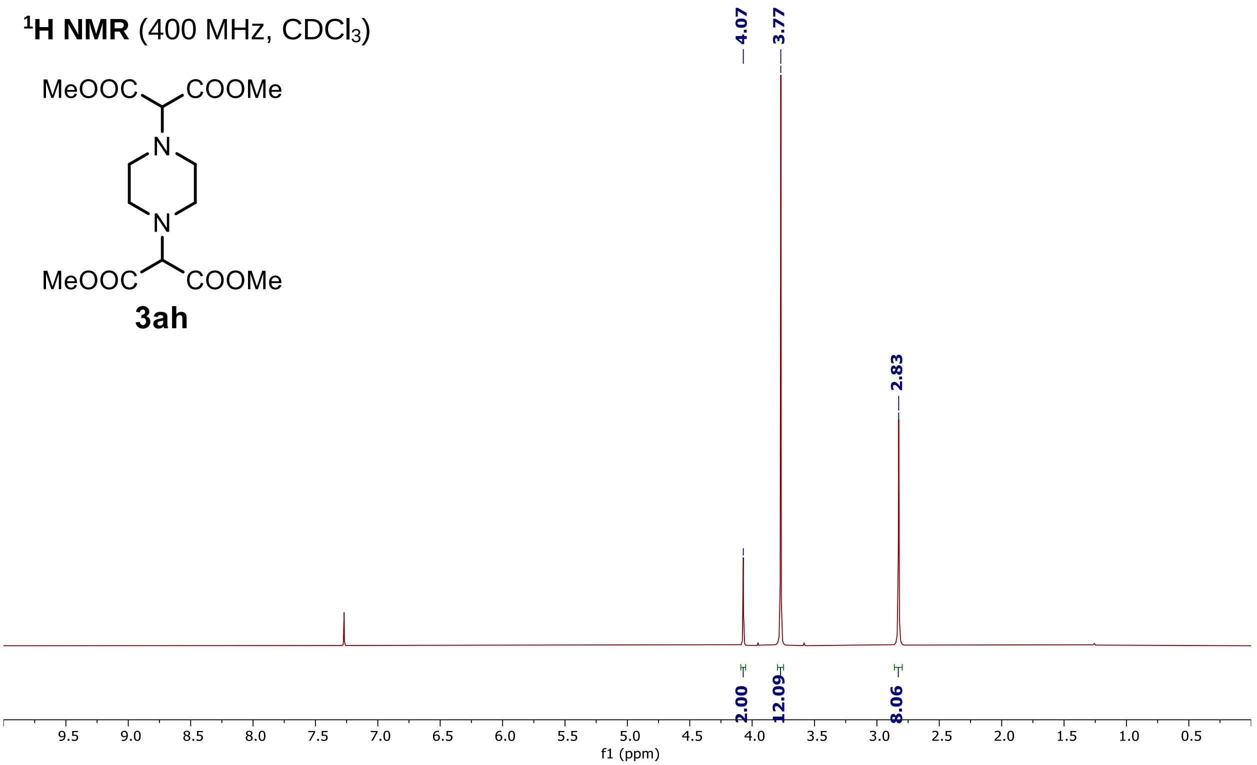

^{1}H NMR (400 MHz, CDCl$_3$)
MeOOC
COOMe
N
N
MeOOC
COOMe
3ah
4.07
3.77
2.83
2.00
12.09
8.06
9.5 9.0 8.5 8.0 7.5 7.0 6.5 6.0 5.5 5.0 4.5 4.0 3.5 3.0 2.5 2.0 1.5 1.0 0.5
f1 (ppm)

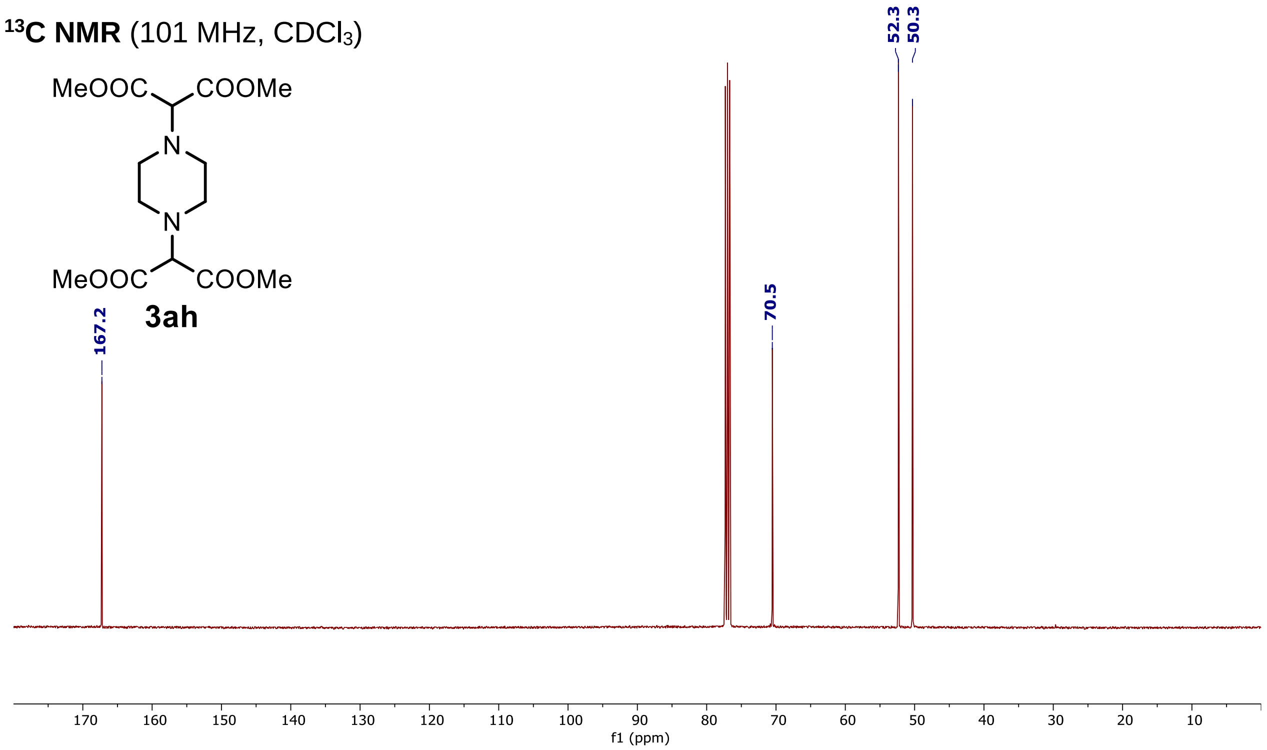

^{13}C NMR (101 MHz, CDCl$_3$)
MeOOC
COOMe
N
N
MeOOC
COOMe
3ah
167.2
70.5
52.3
50.3
170 160 150 140 130 120 110 100 90 80 70 60 50 40 30 20 10
f1 (ppm)

^{1}H NMR (500 MHz, $CDCl_3$)

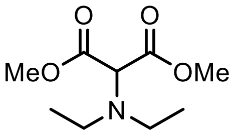

3ai

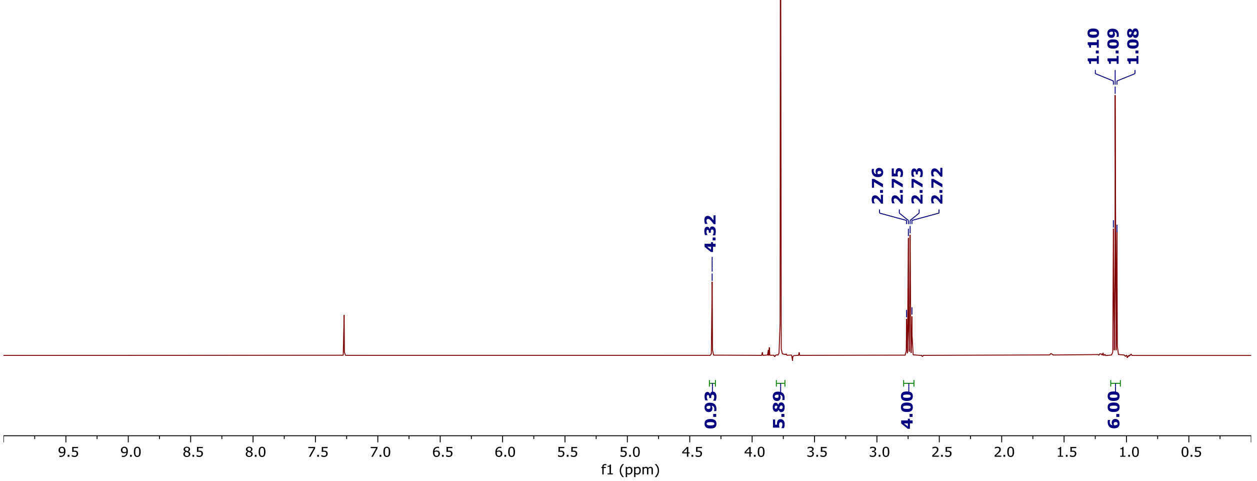

^{13}C NMR (126 MHz, $CDCl_3$)

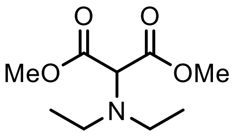

3ai

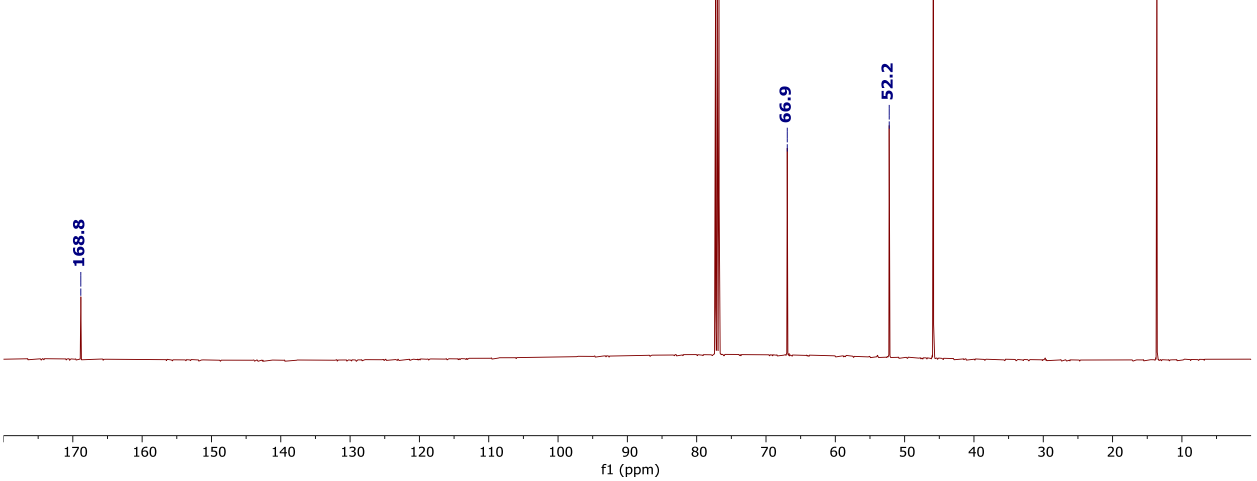

^{1}H NMR (400 MHz, $CDCl_3$)

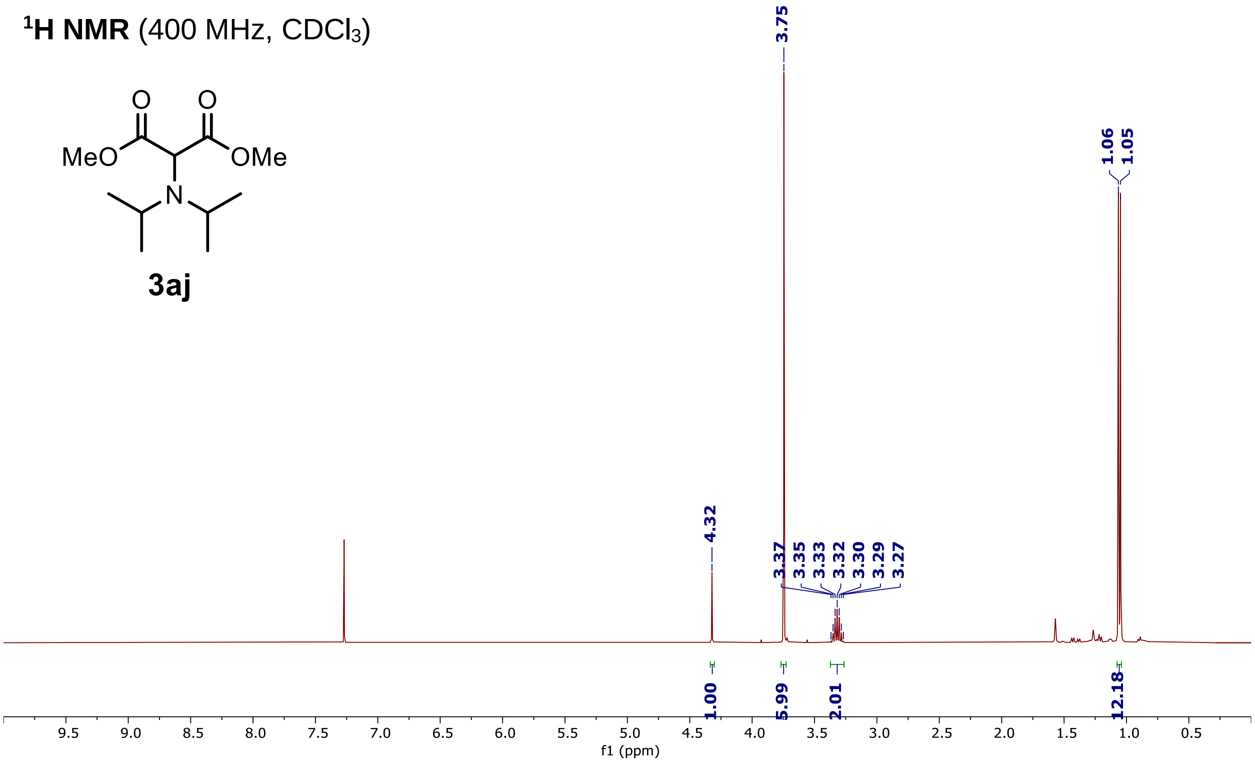

3aj

^{13}C NMR (101 MHz, $CDCl_3$)

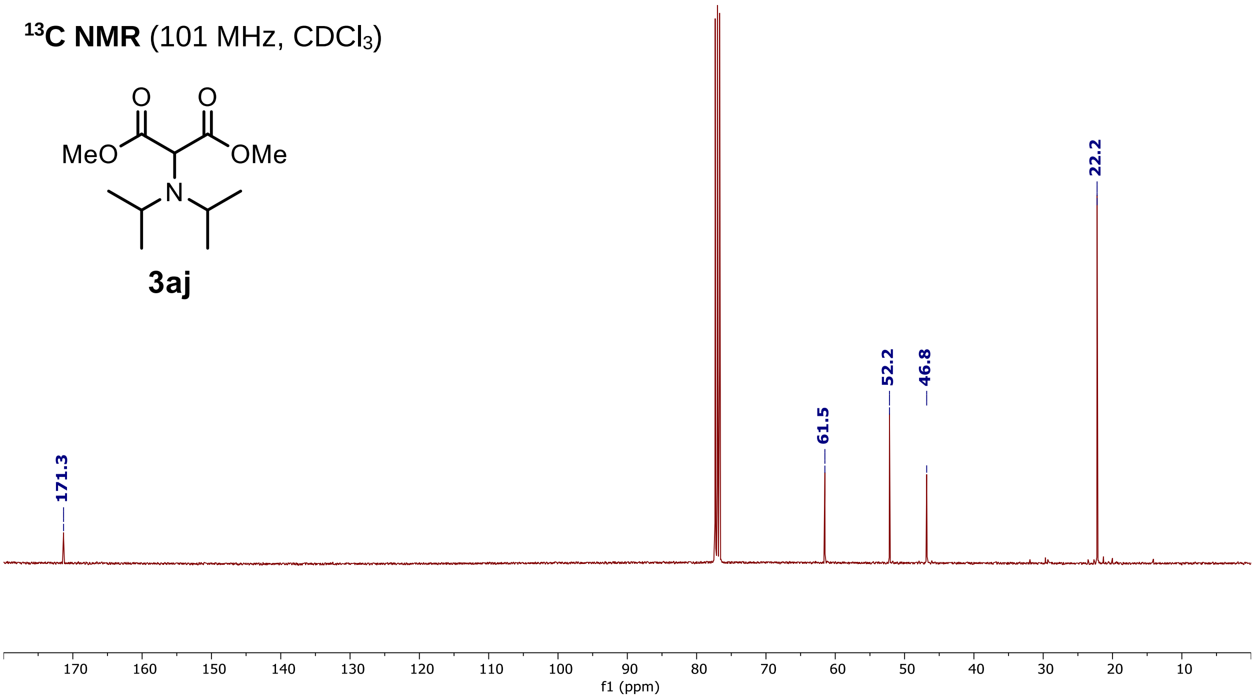

3aj

^{1}H NMR (500 MHz, $CDCl_3$)

3ak

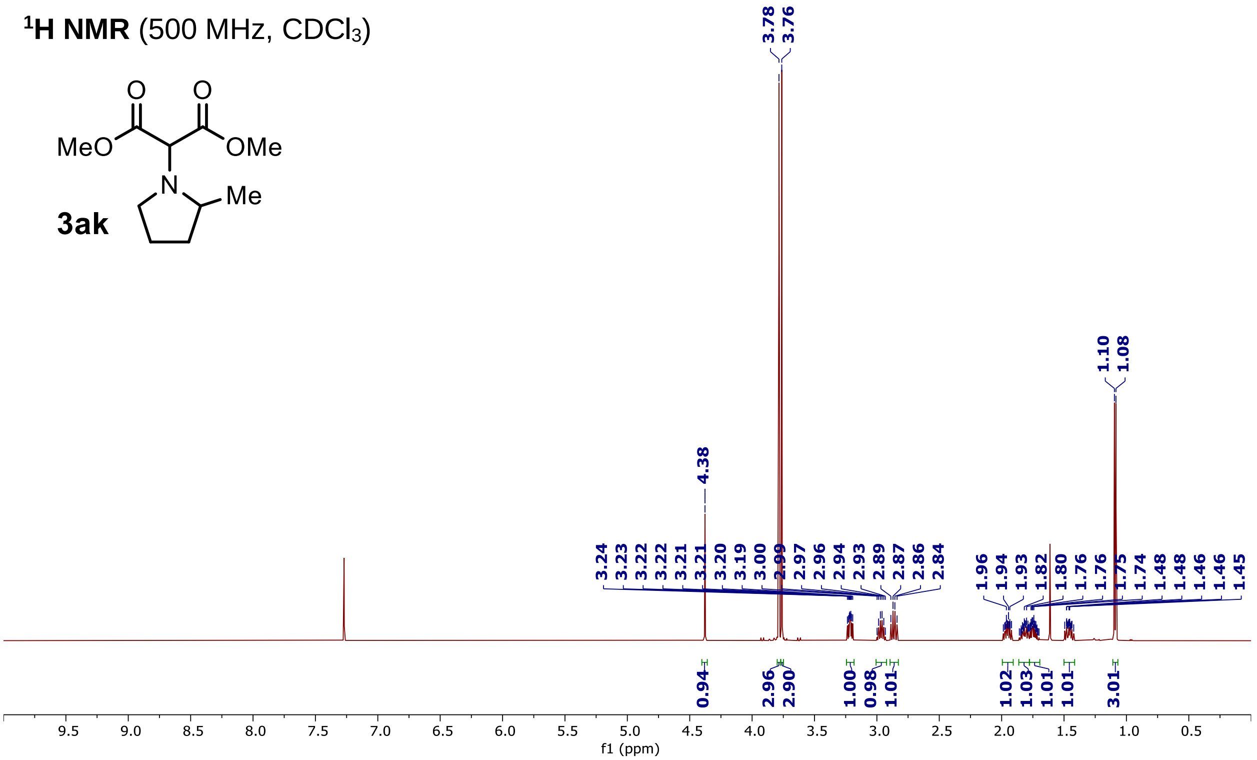

^{13}C NMR (126 MHz, $CDCl_3$)

3ak

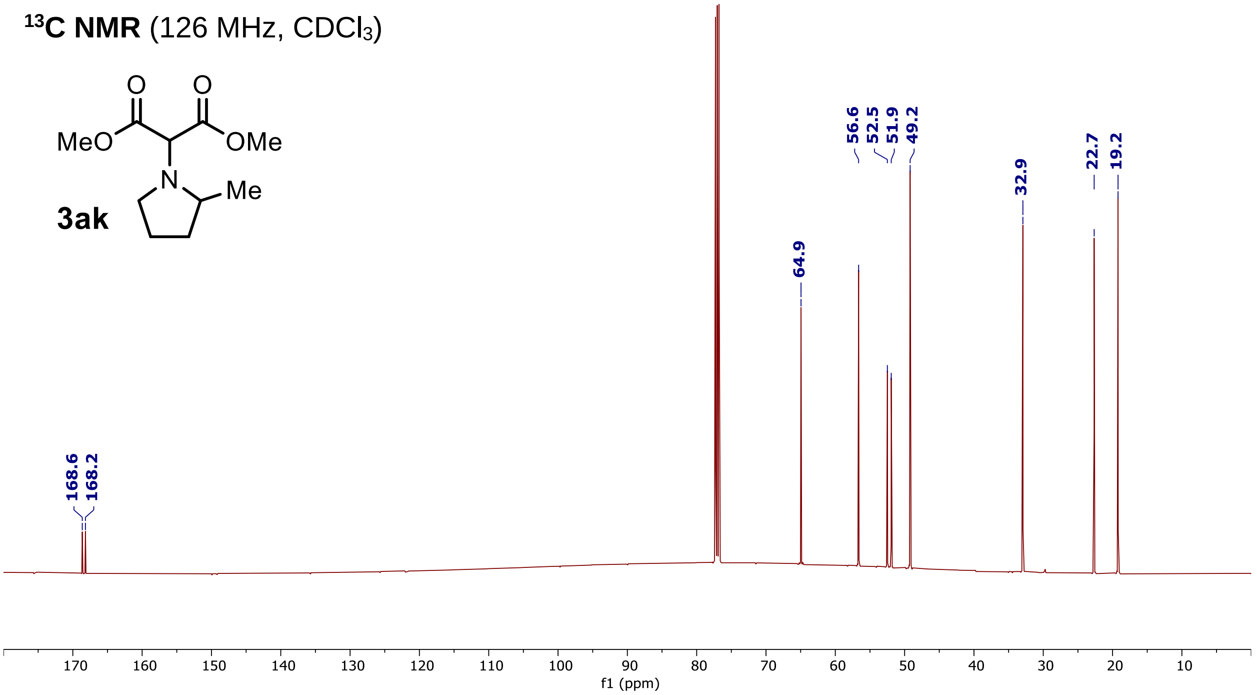

¹H NMR (500 MHz, $CDCl_3$)

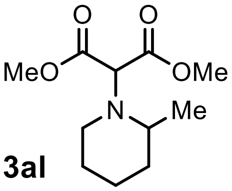

3al

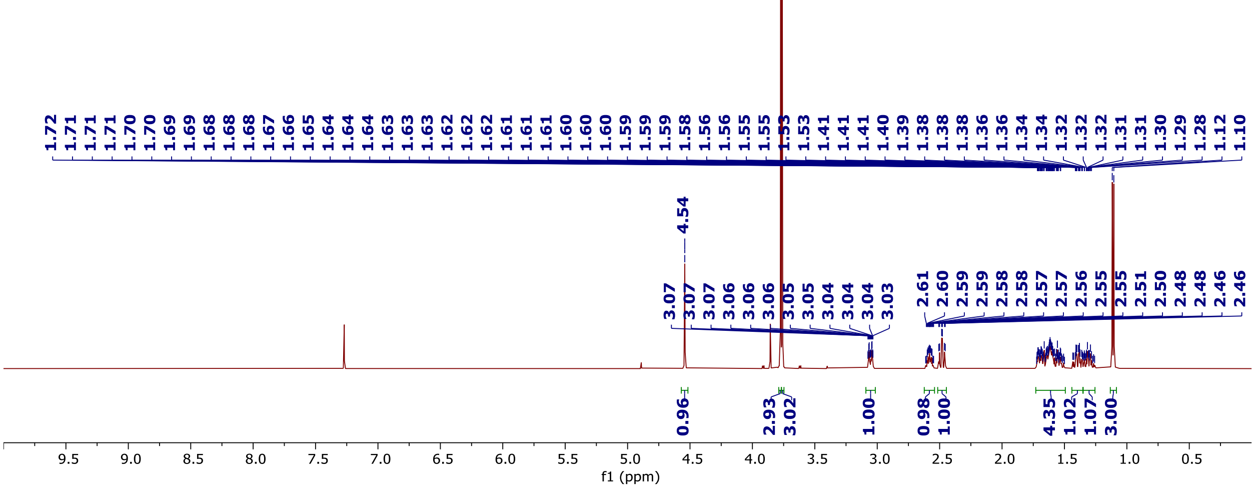

¹³C NMR (126 MHz, $CDCl_3$)

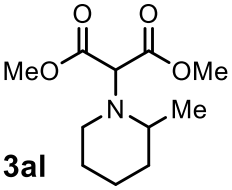

3al

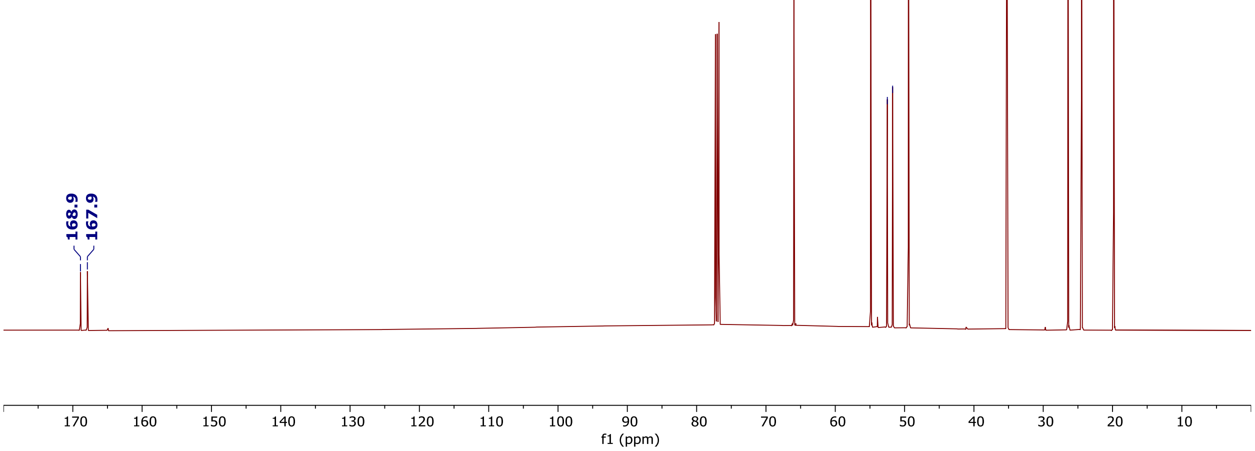

^{1}H NMR (500 MHz, $CDCl_3$)

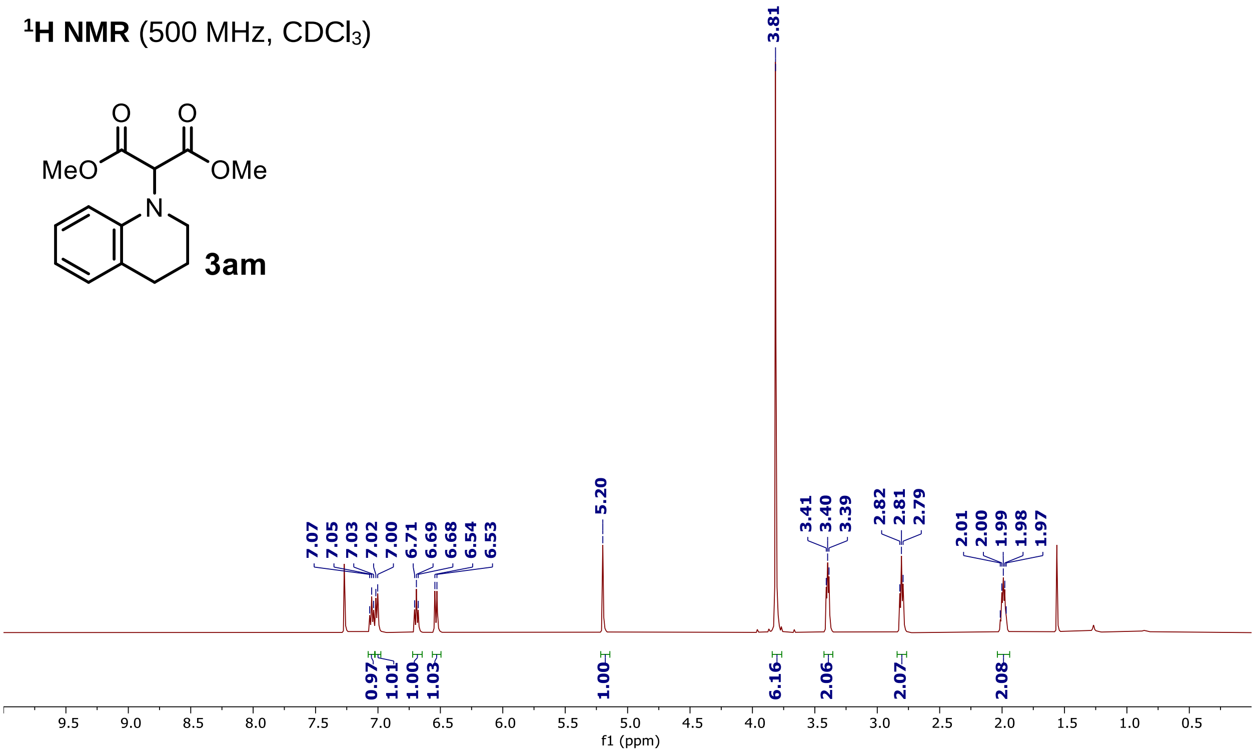

^{13}C NMR (126 MHz, $CDCl_3$)

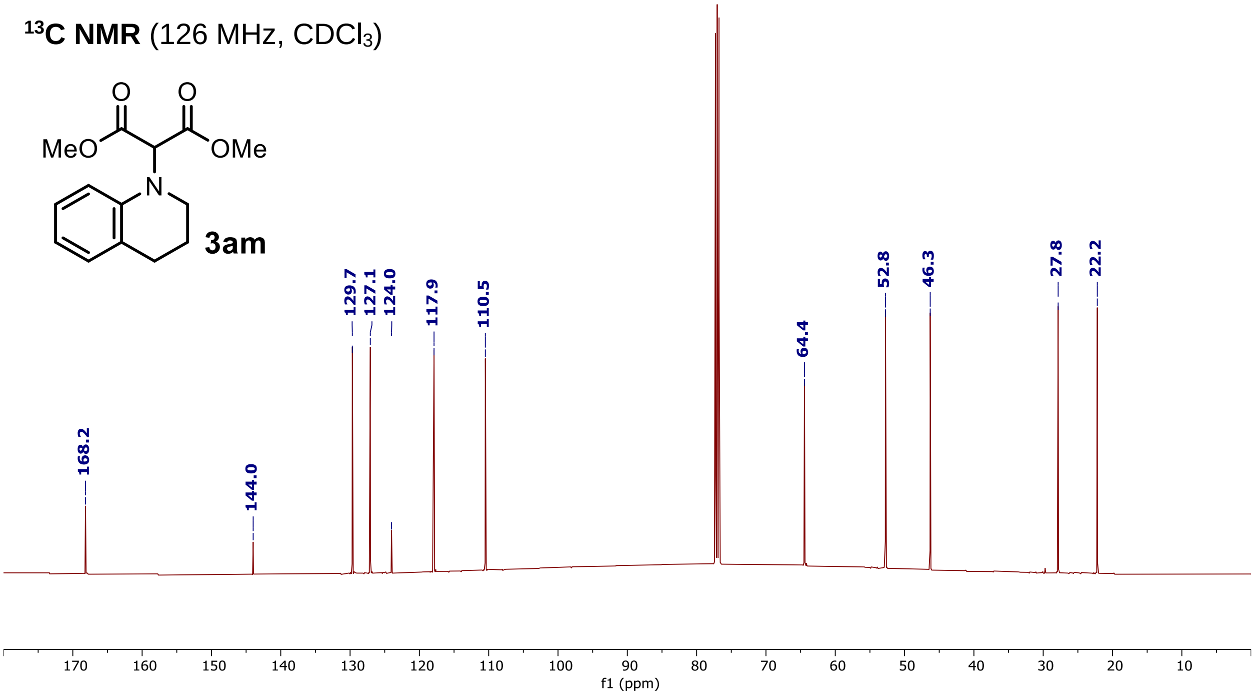

^{1}**H NMR** (500 MHz, $CDCl_3$)

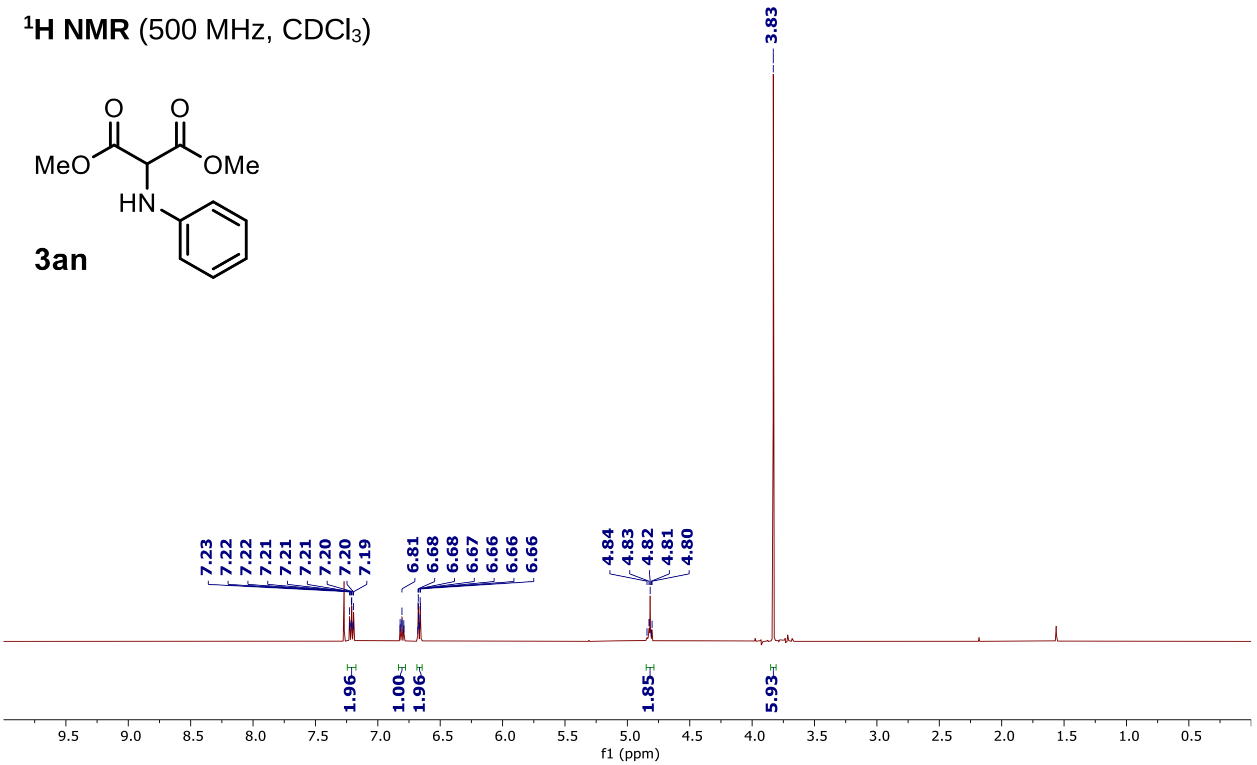

^{13}**C NMR** (126 MHz, $CDCl_3$)

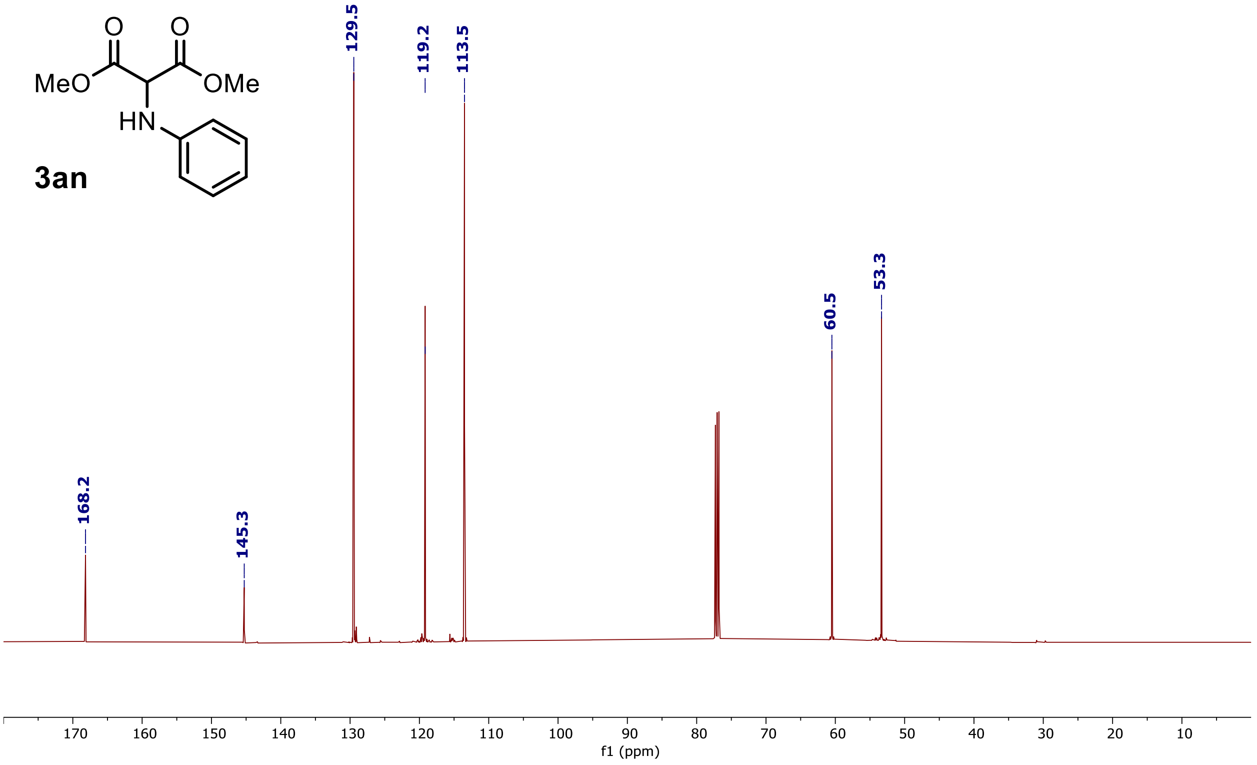

^{1}H NMR (500 MHz, $CDCl_3$)

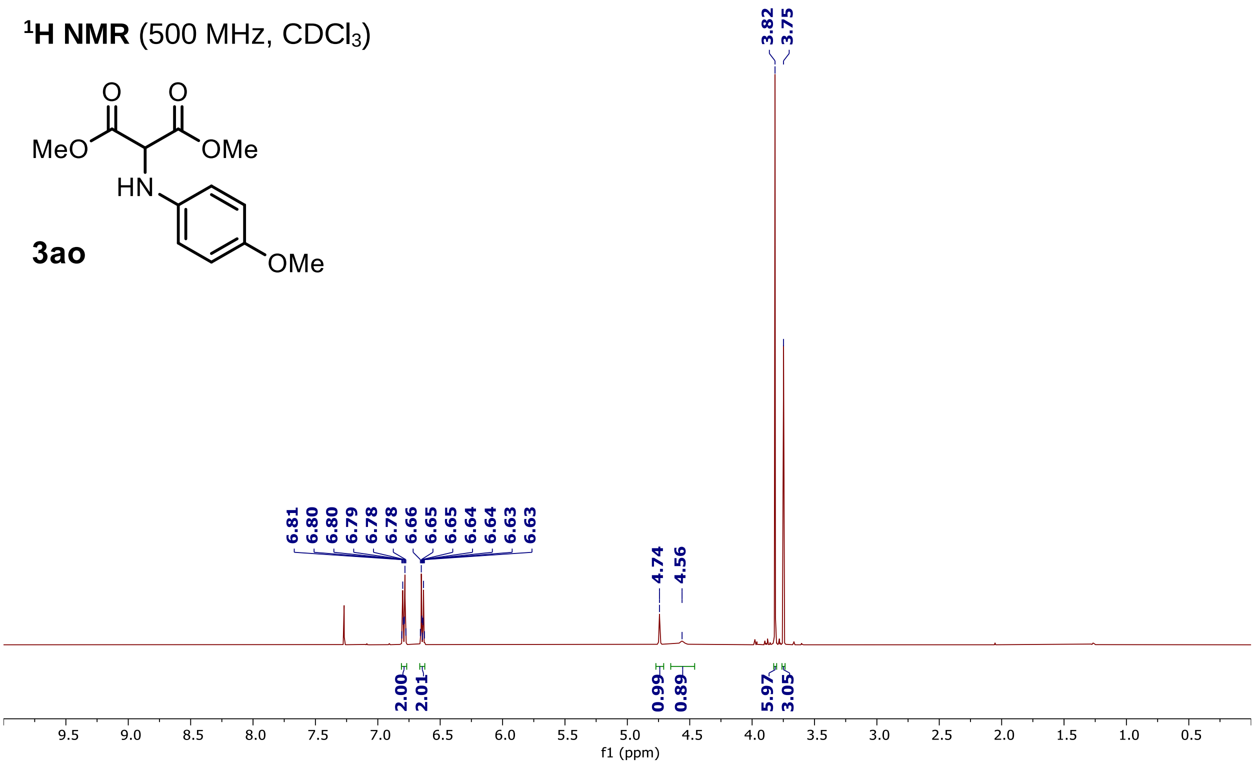

3ao

^{13}C NMR (126 MHz, $CDCl_3$)

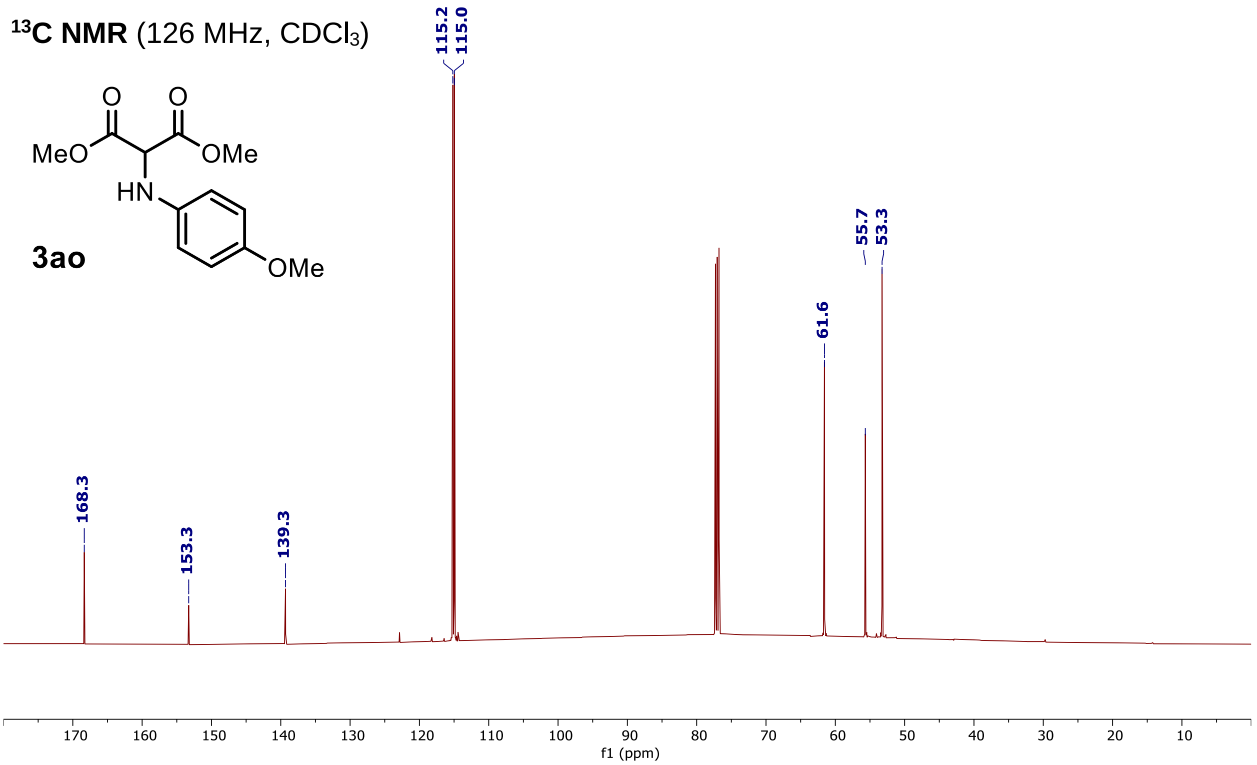

3ao

^{1}H NMR (500 MHz, $CDCl_3$)

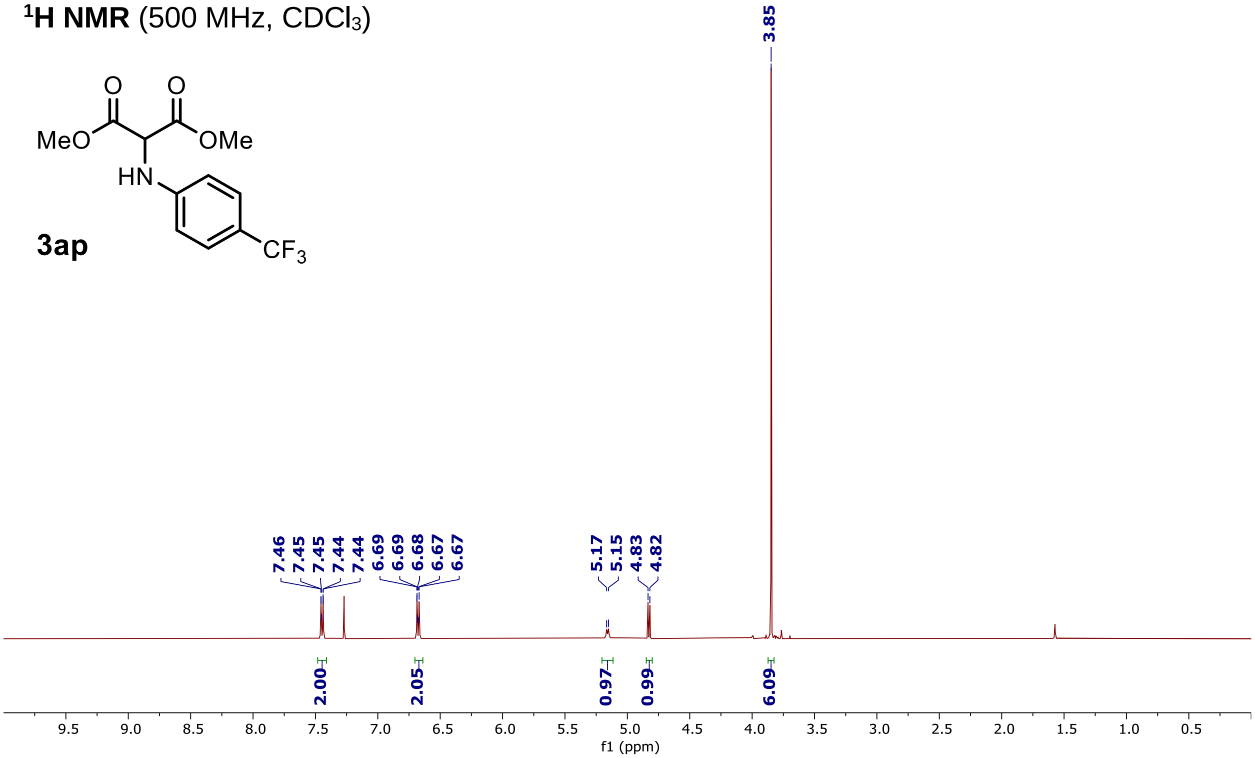

^{13}C NMR (126 MHz, $CDCl_3$)

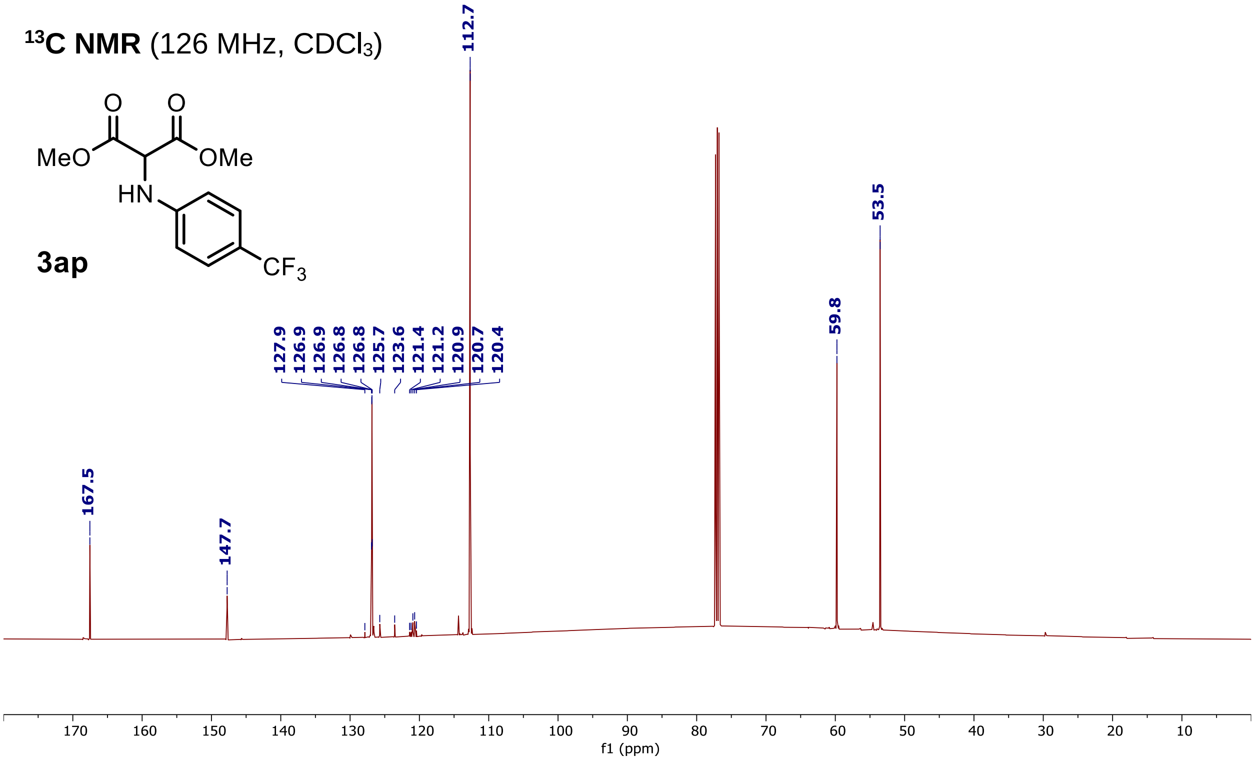

^{19}F NMR (282 MHz, $CDCl_3$)

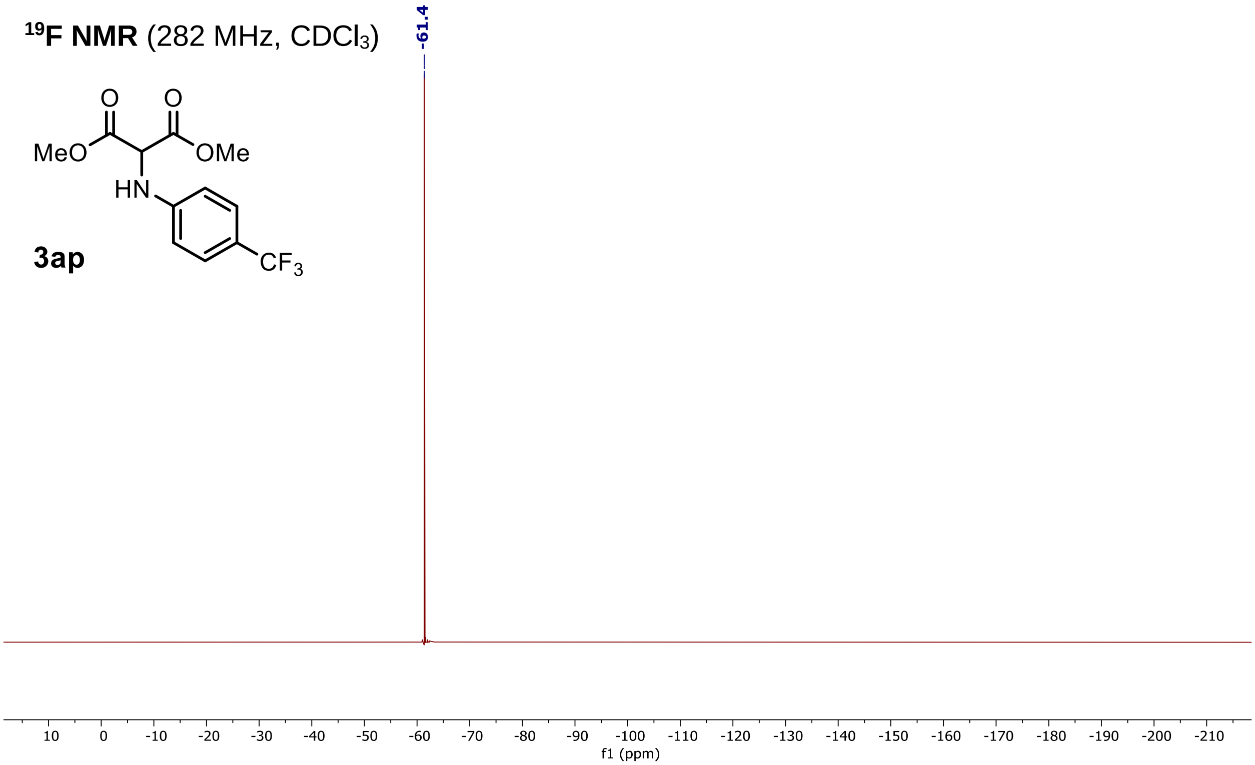

3ap

^{1}H NMR (500 MHz, $CDCl_3$)

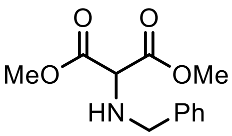

3aq

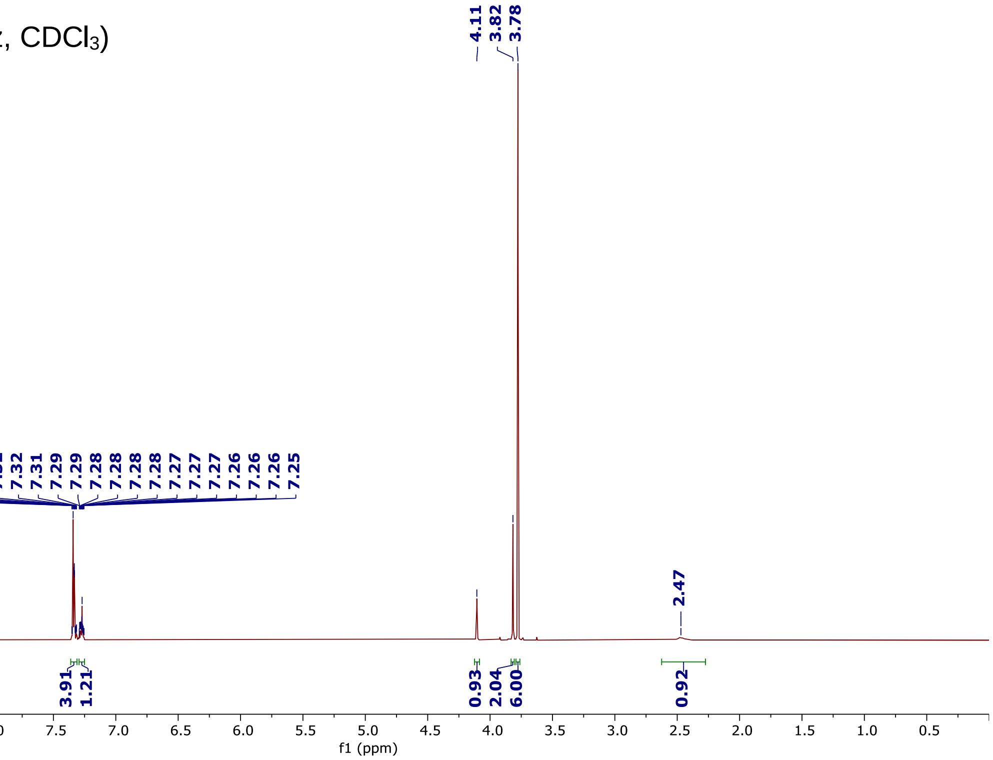

^{13}C NMR (126 MHz, $CDCl_3$)

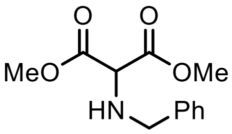

3aq

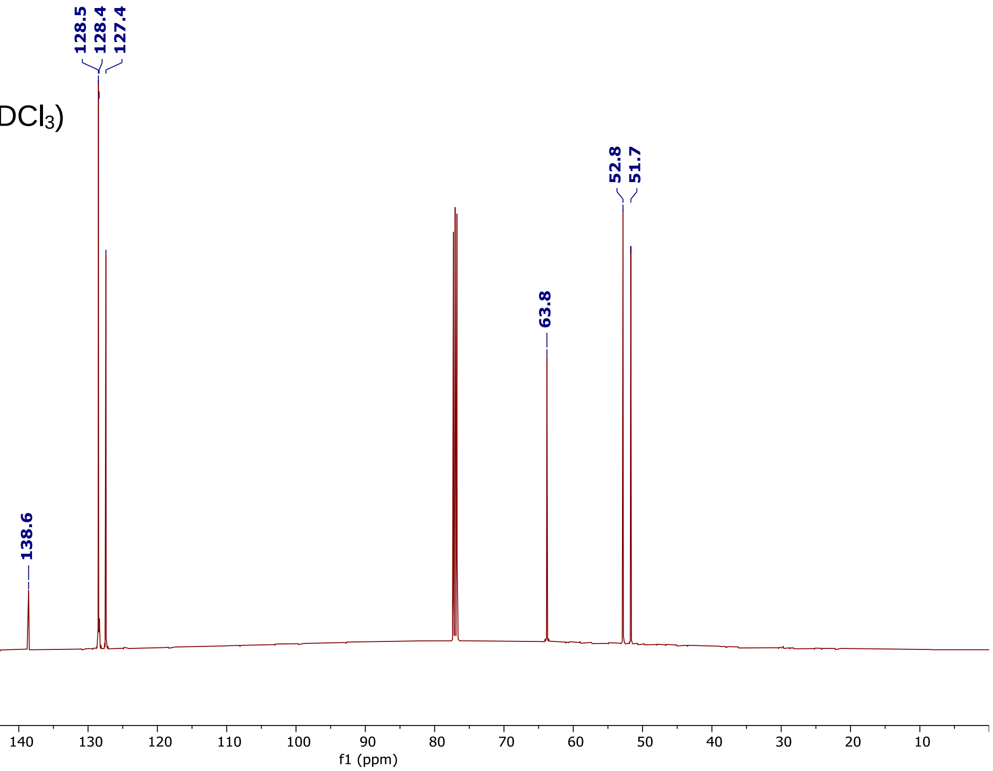

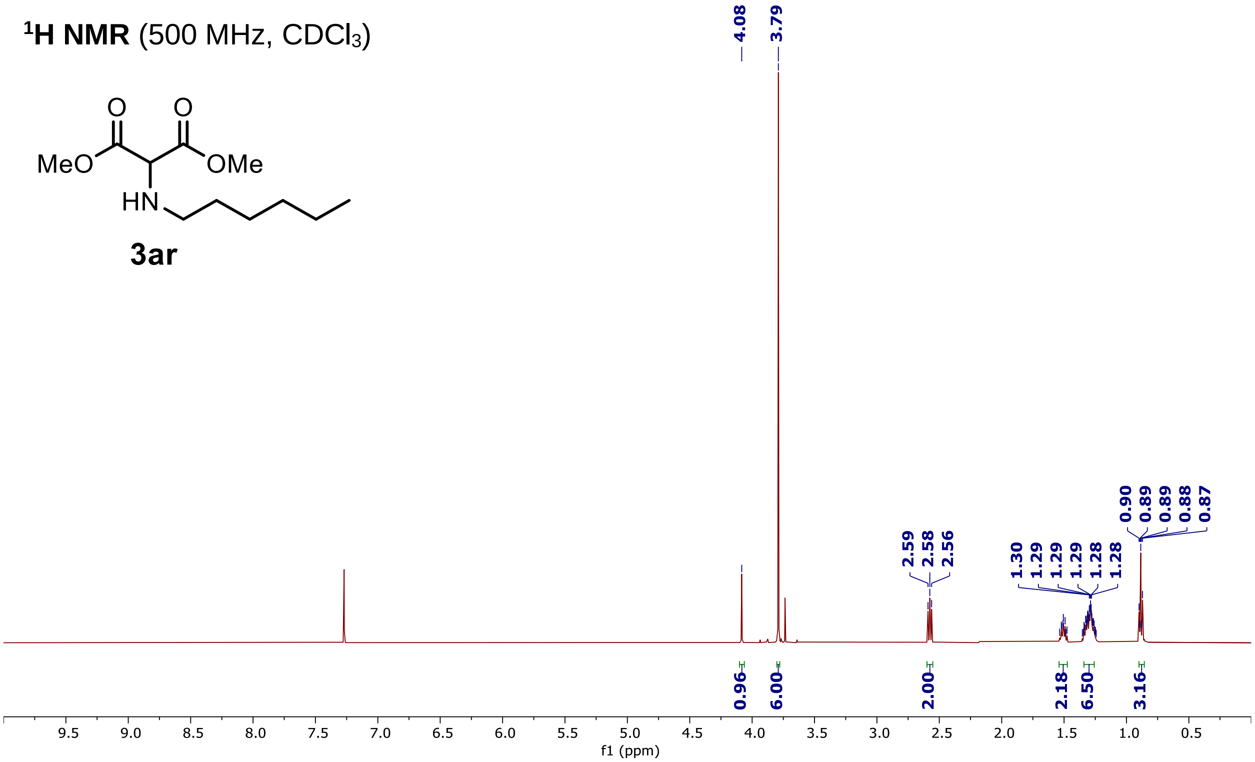
1H NMR (500 MHz, CDCl3)
O O
MeO OMe
HN
3ar
4.08
3.79
2.59 2.58 2.56
1.30 1.29 1.29 1.29 1.28 1.28
0.90 0.89 0.89 0.88 0.87
0.96
6.00
2.00
2.18
6.50
3.16
9.5 9.0 8.5 8.0 7.5 7.0 6.5 6.0 5.5 5.0 4.5 4.0 3.5 3.0 2.5 2.0 1.5 1.0 0.5
f1 (ppm)

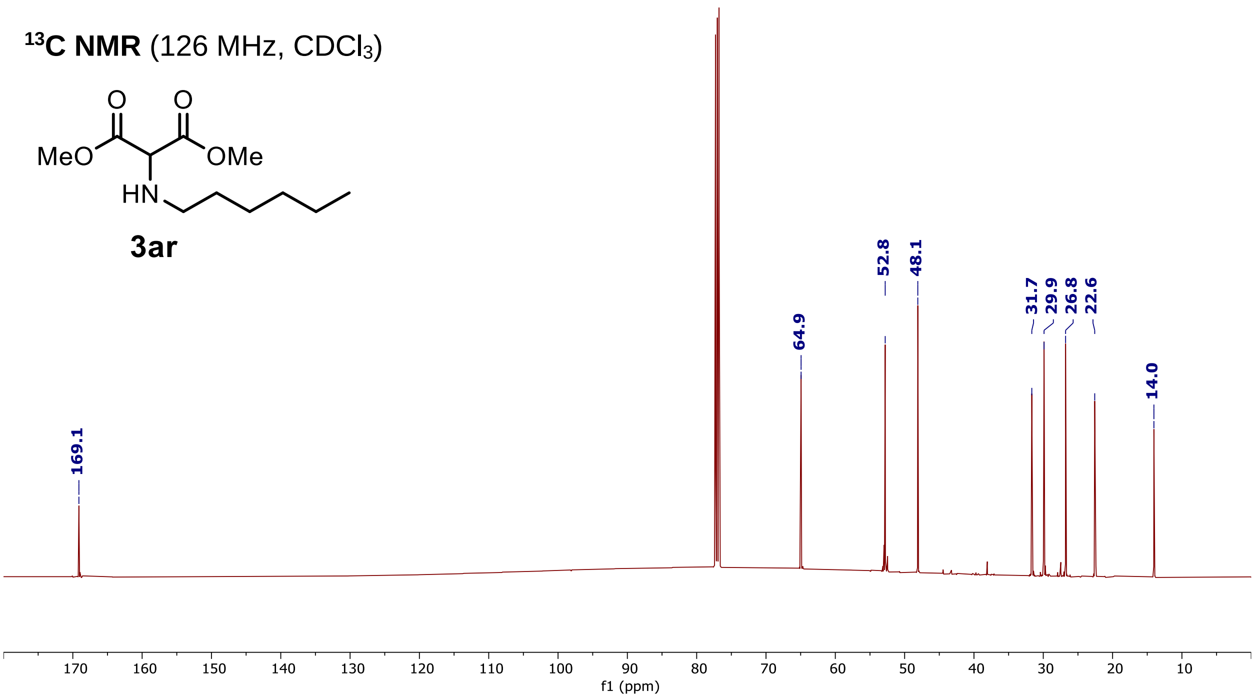
13C NMR (126 MHz, CDCl3)
O O
MeO OMe
HN
3ar
169.1
64.9
52.8
48.1
31.7 29.9 26.8 22.6
14.0
170 160 150 140 130 120 110 100 90 80 70 60 50 40 30 20 10
f1 (ppm)

^{1}H NMR (500 MHz, $CDCl_3$)

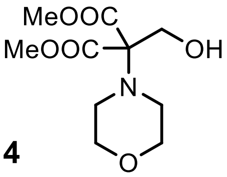

4

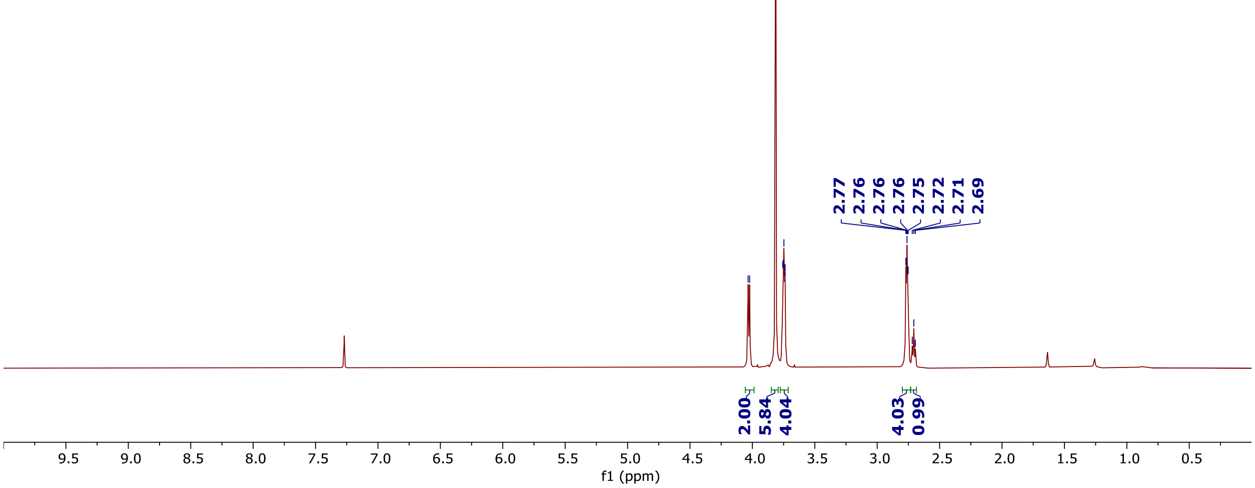

^{13}C NMR (126 MHz, $CDCl_3$)

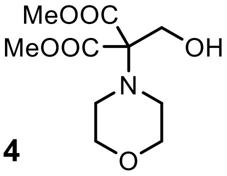

4

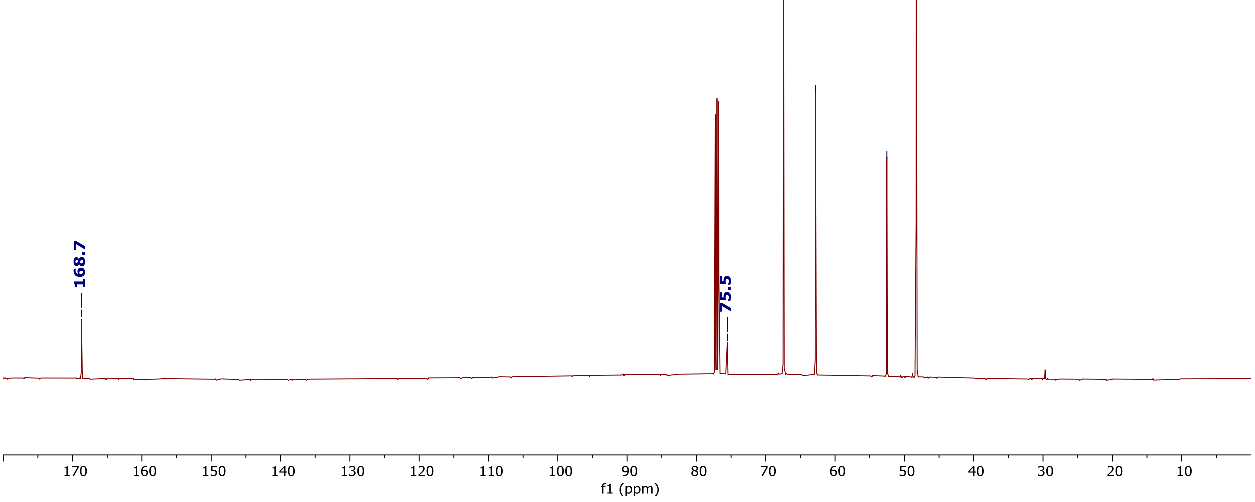

^{1}H NMR (500 MHz, $CDCl_3$)

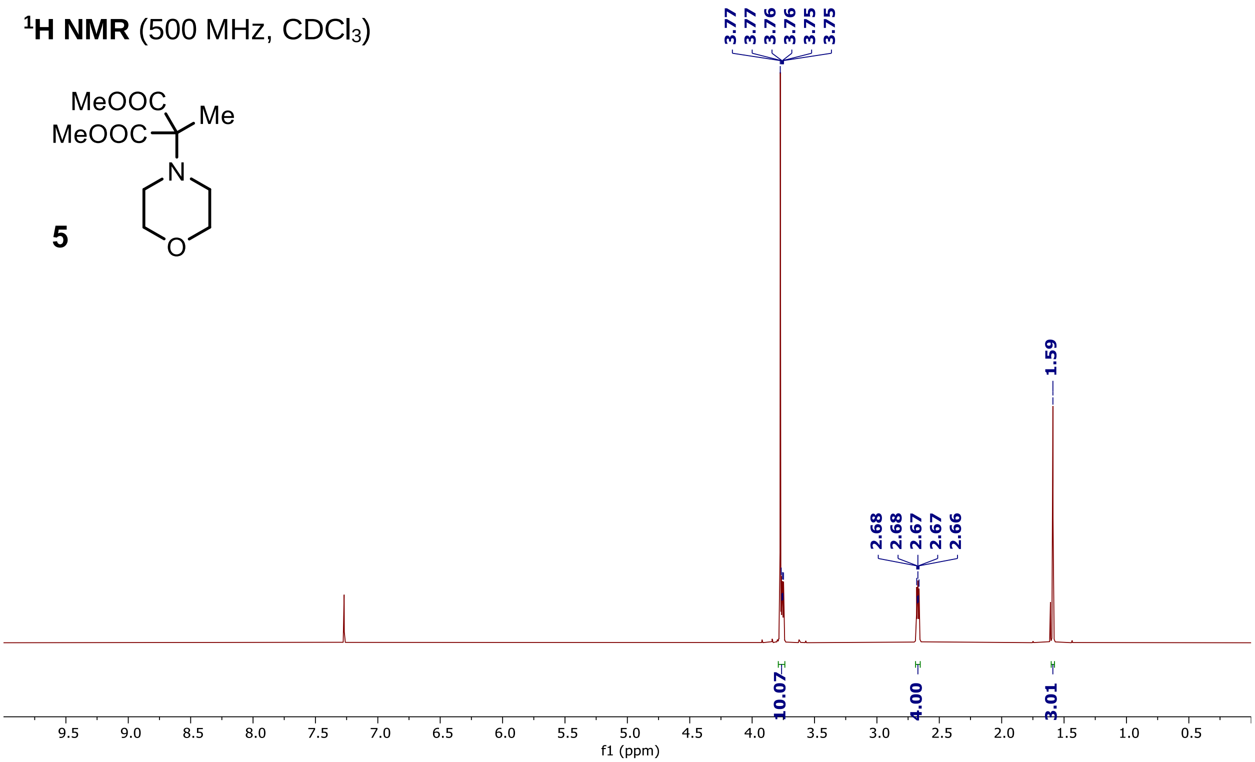

^{13}C NMR (126 MHz, $CDCl_3$)

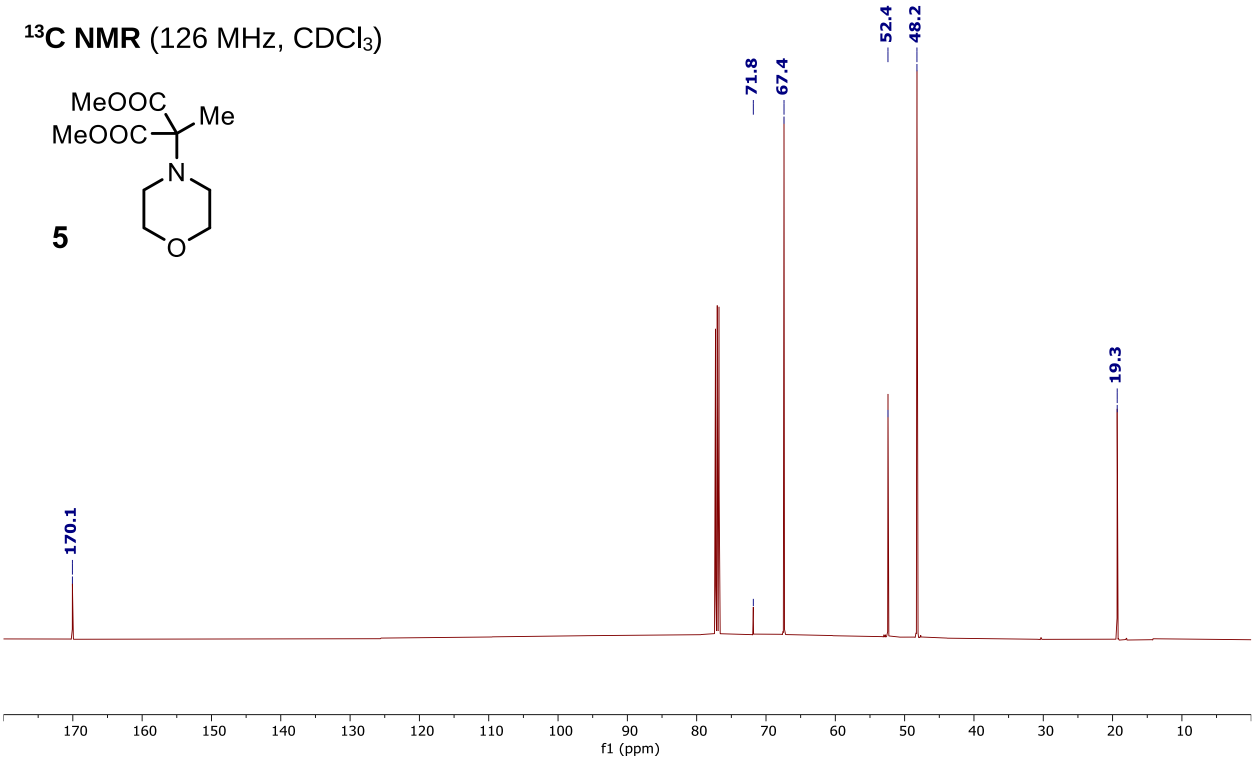

^{1}**H NMR** (500 MHz, $CDCl_3$)

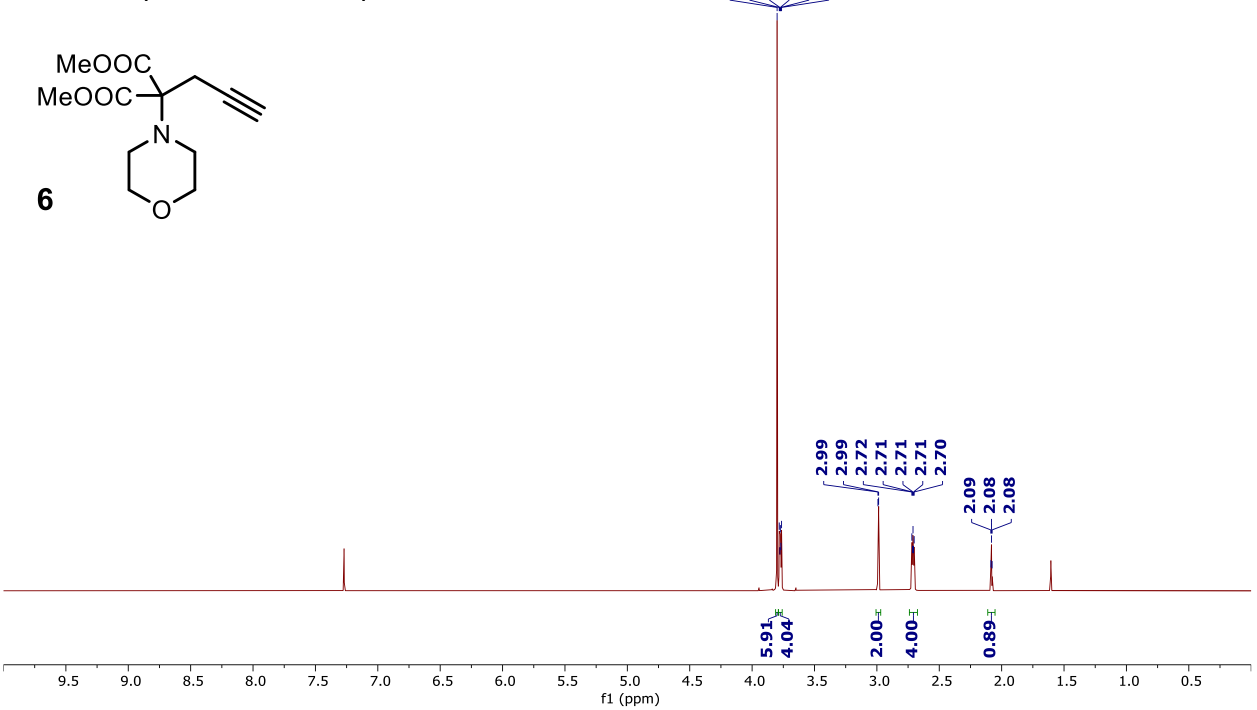

^{13}**C NMR** (126 MHz, $CDCl_3$)

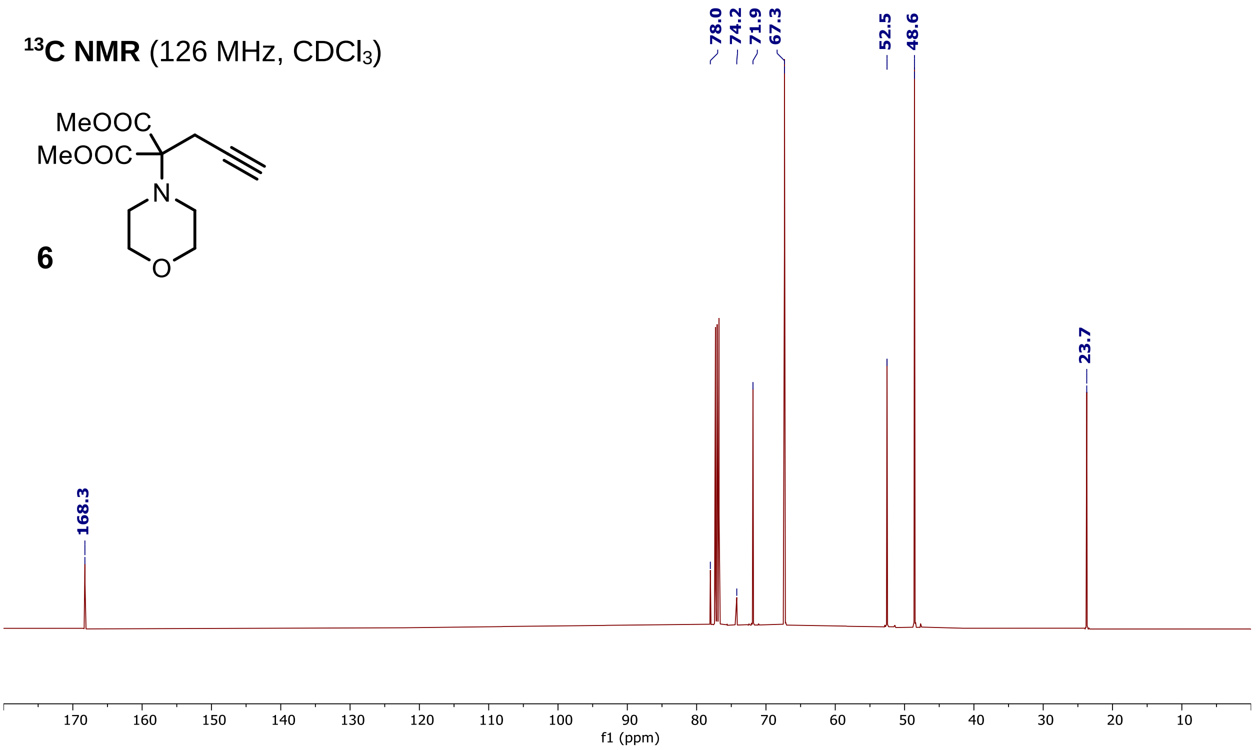

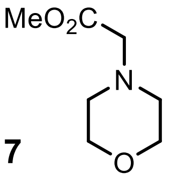

7

Compound **7** has been previously reported in the literature.[10]

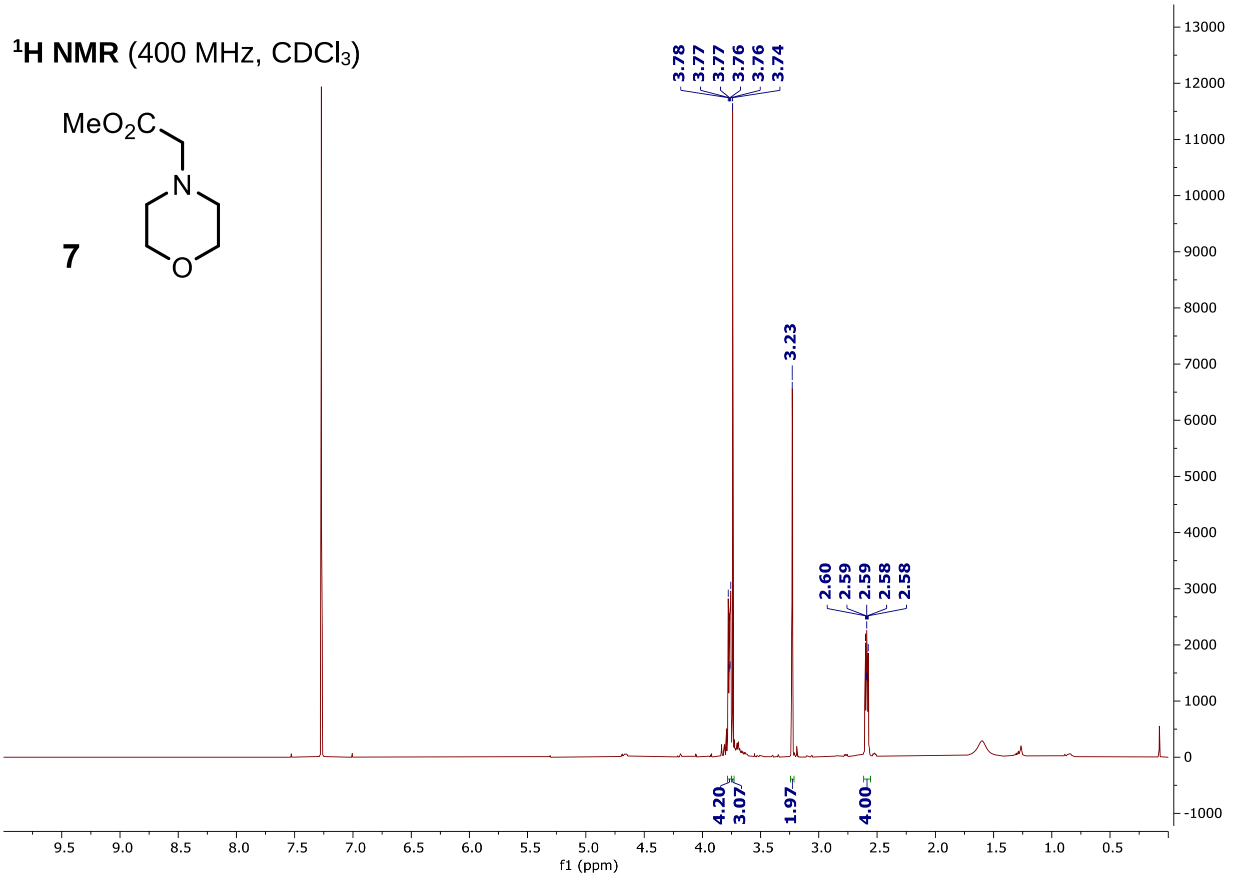

^{1}H NMR (500 MHz, $CDCl_3$)

8

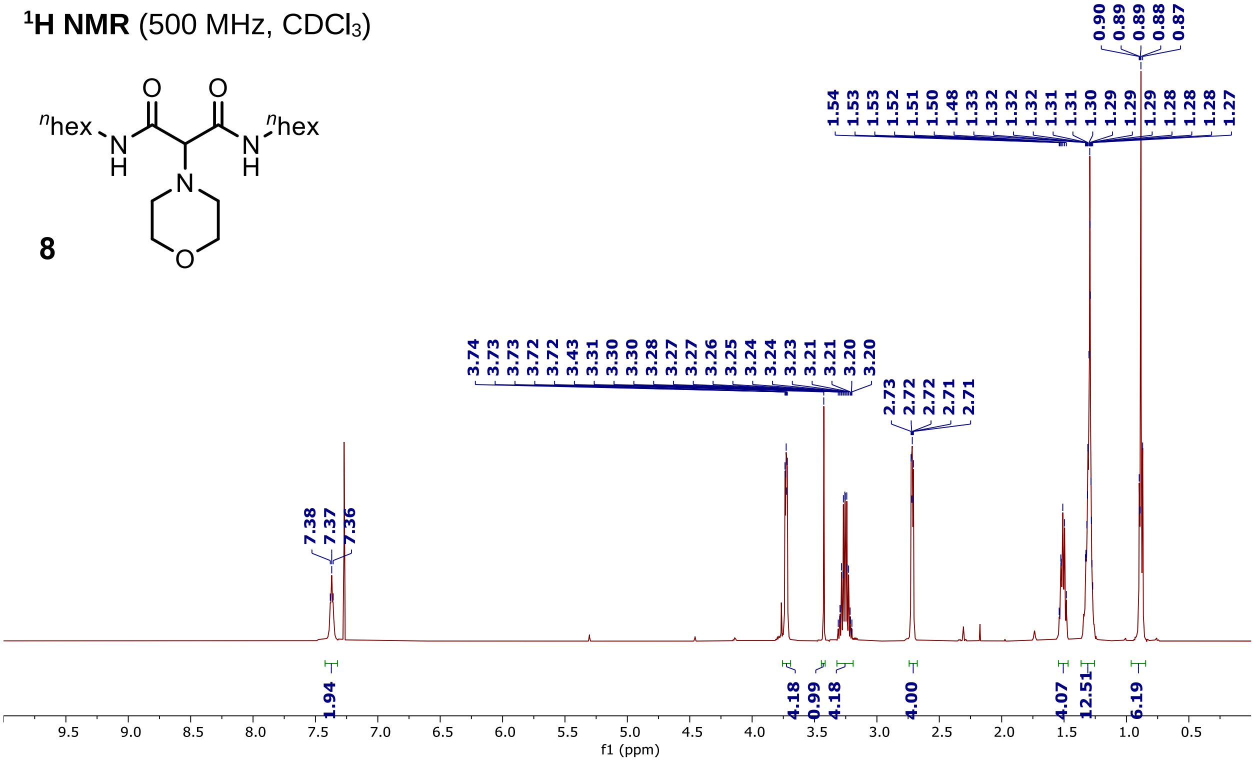

^{13}C NMR (126 MHz, $CDCl_3$)

8

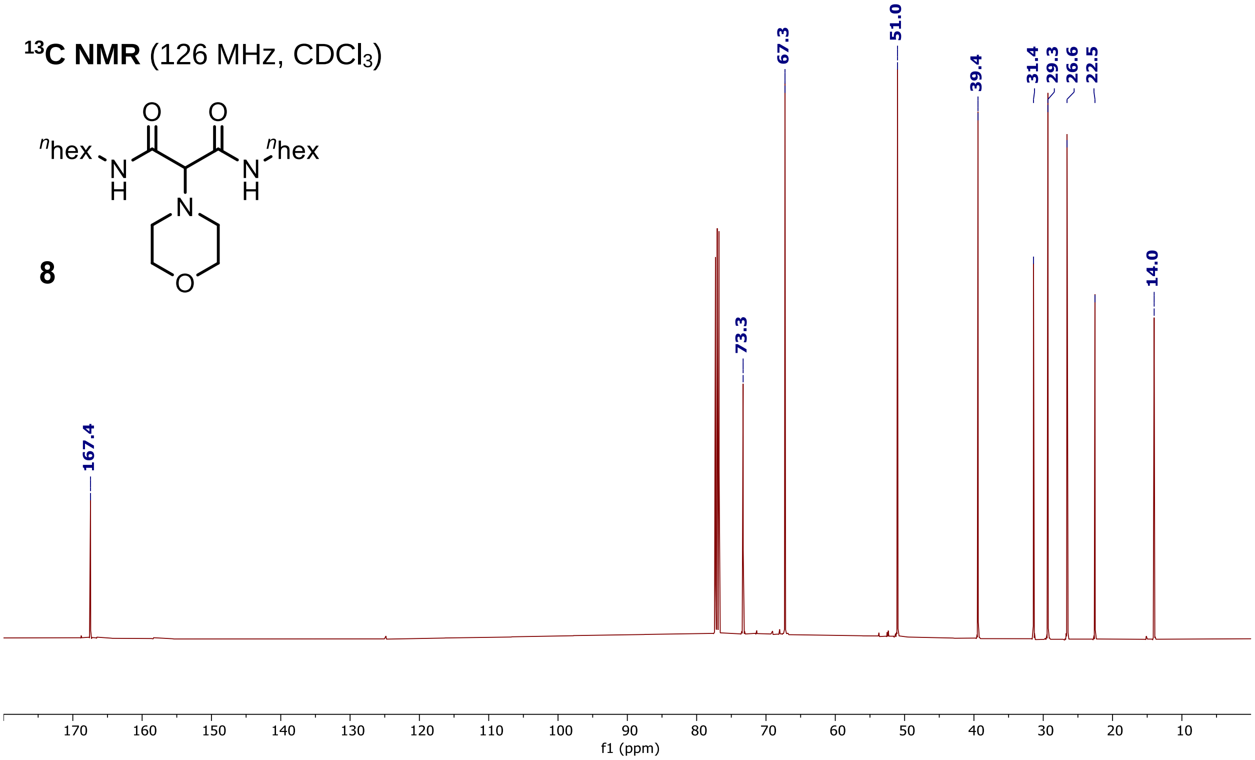

^{1}H NMR (500 MHz, $CDCl_3$)

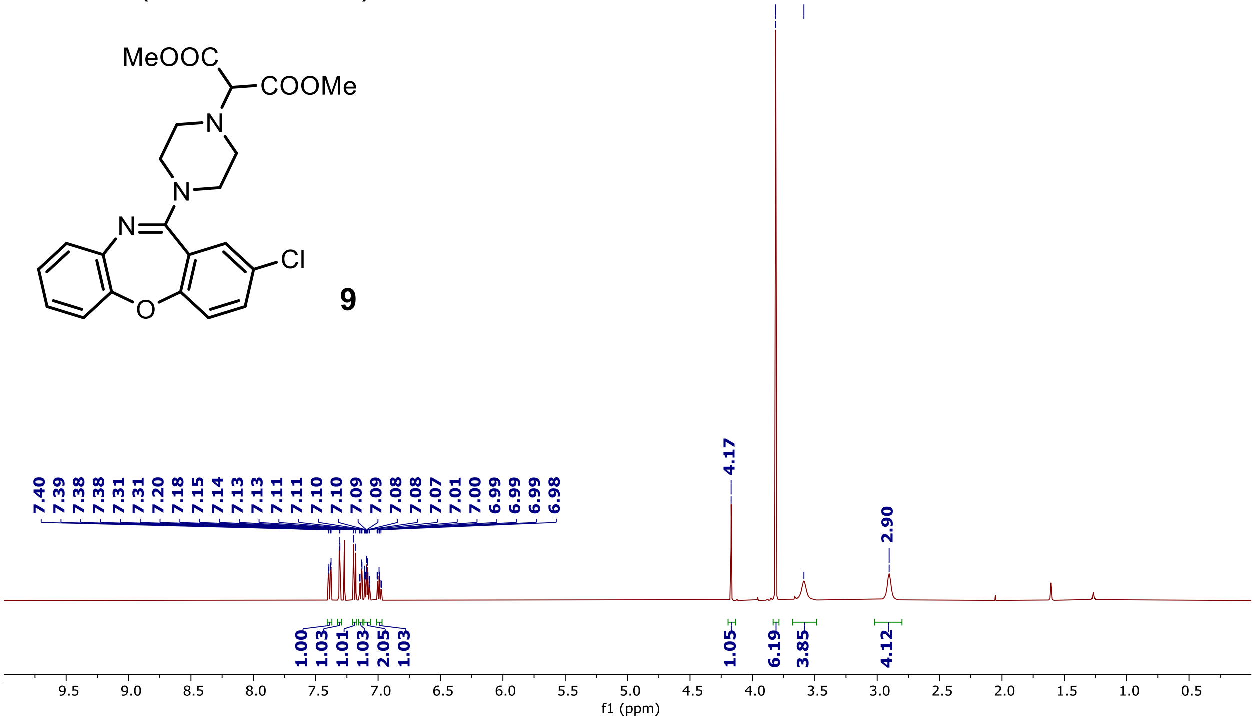

^{13}C NMR (126 MHz, $CDCl_3$)

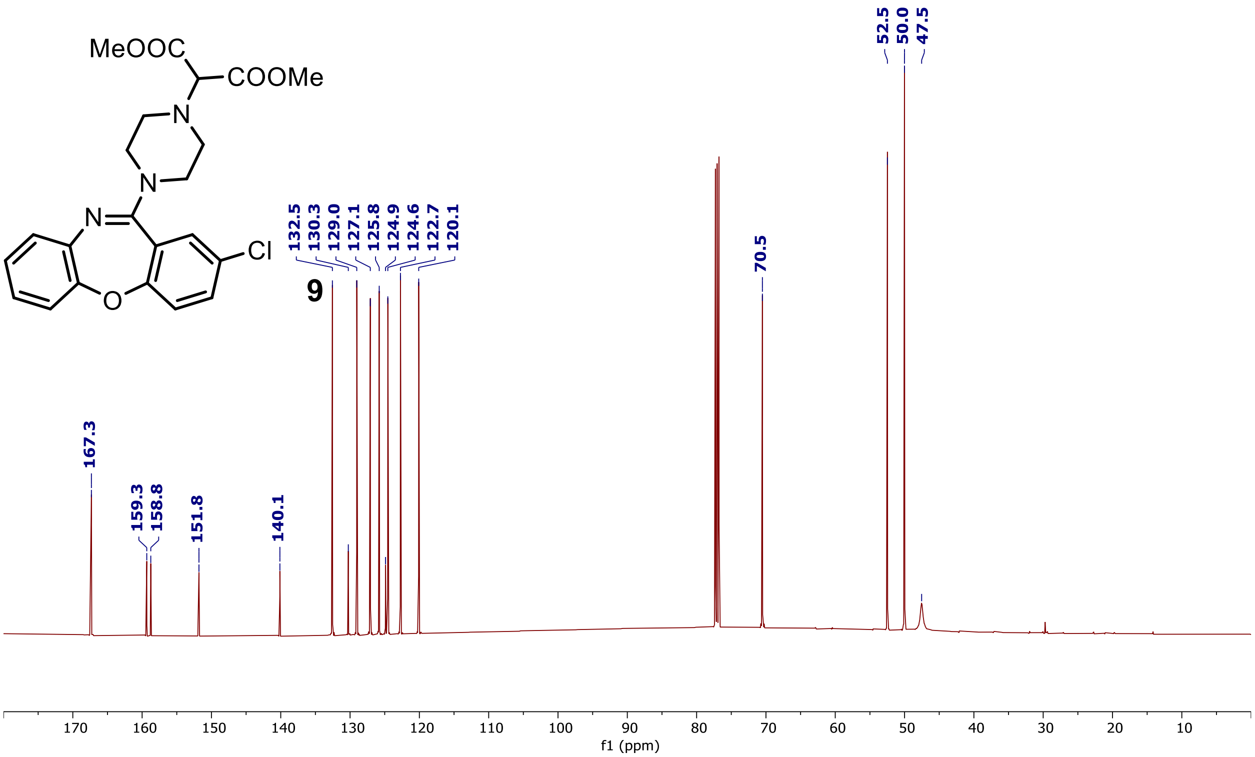

^{1}H NMR (500 MHz, $CDCl_3$)

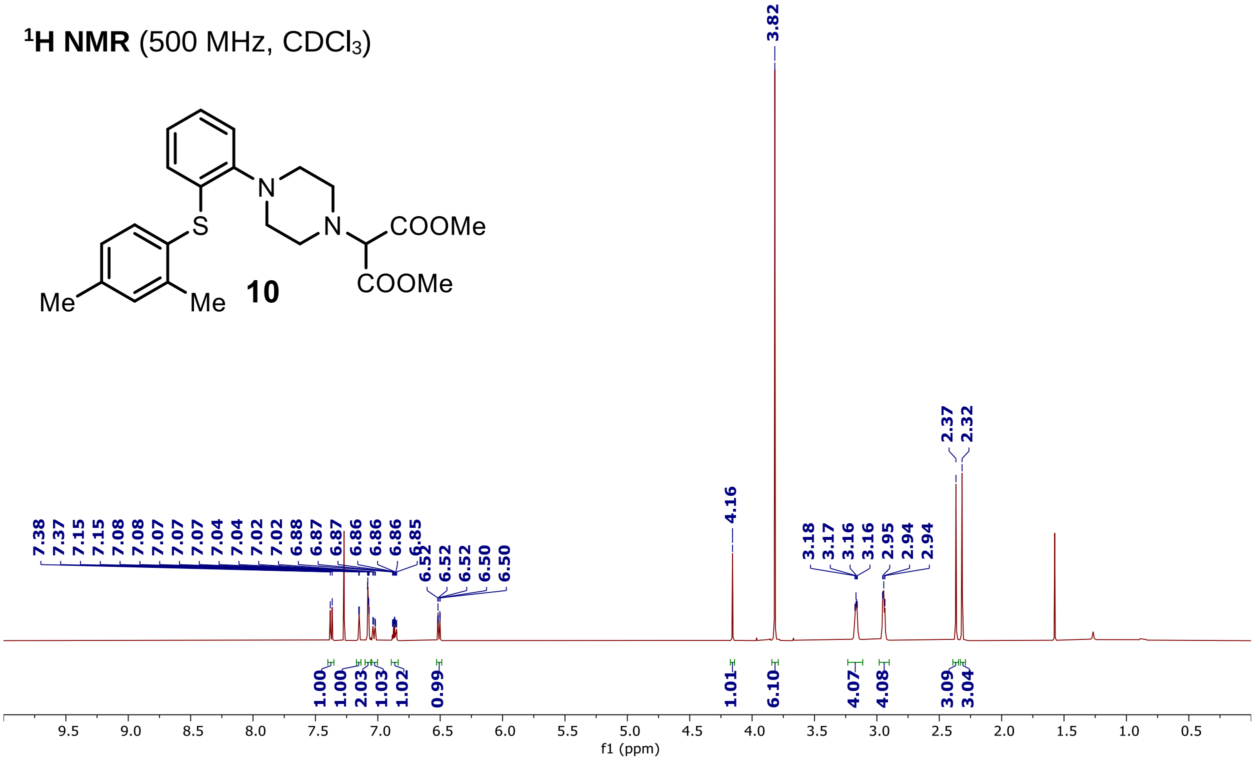

^{13}C NMR (126 MHz, $CDCl_3$)

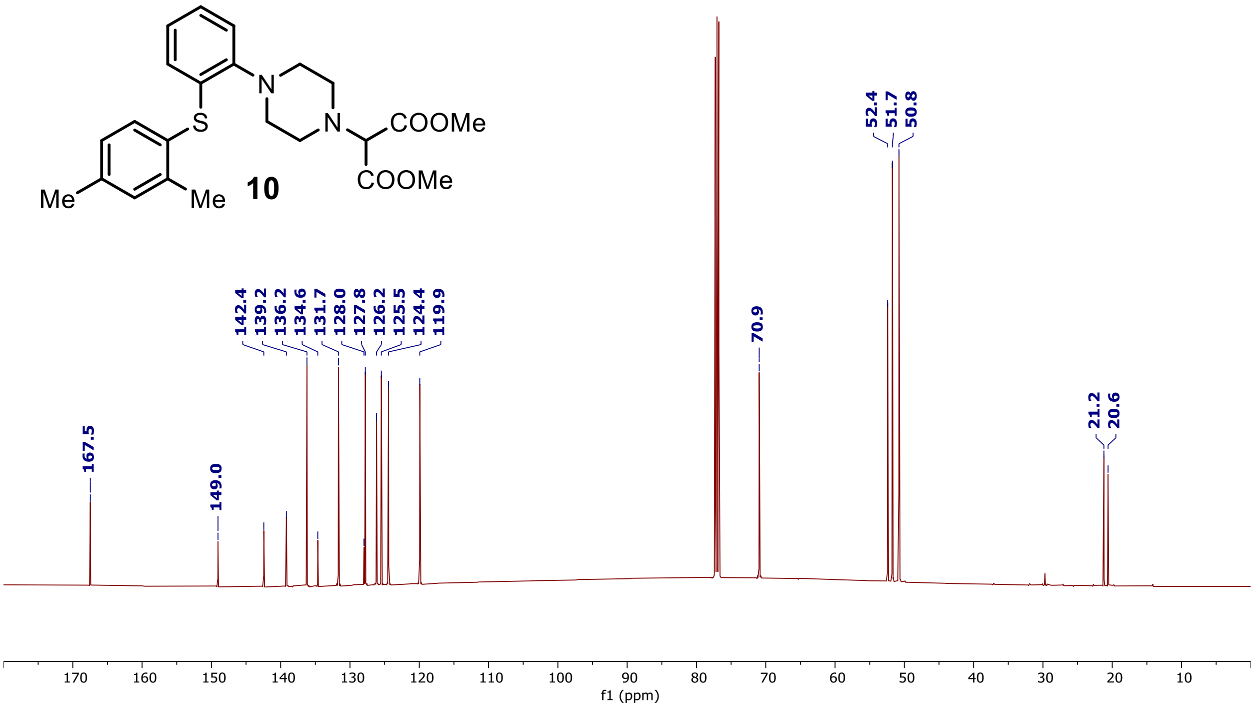

^{1}H NMR (500 MHz, $CDCl_3$)

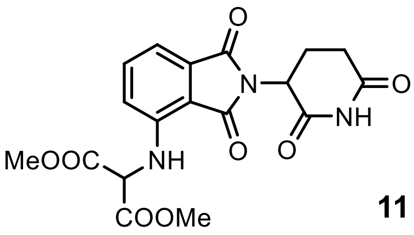

11

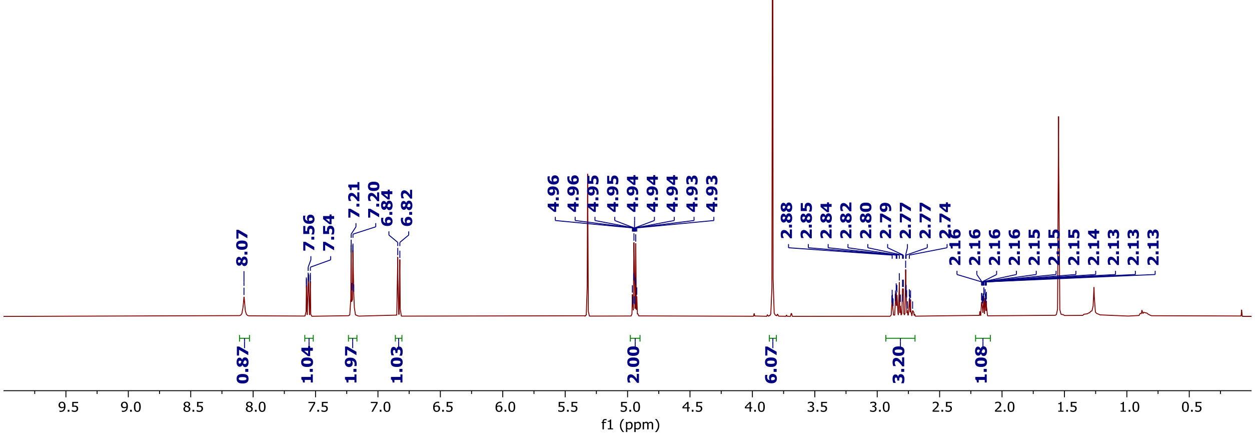

^{13}C NMR (126 MHz, $CDCl_3$)

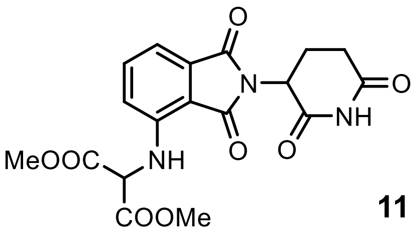

11

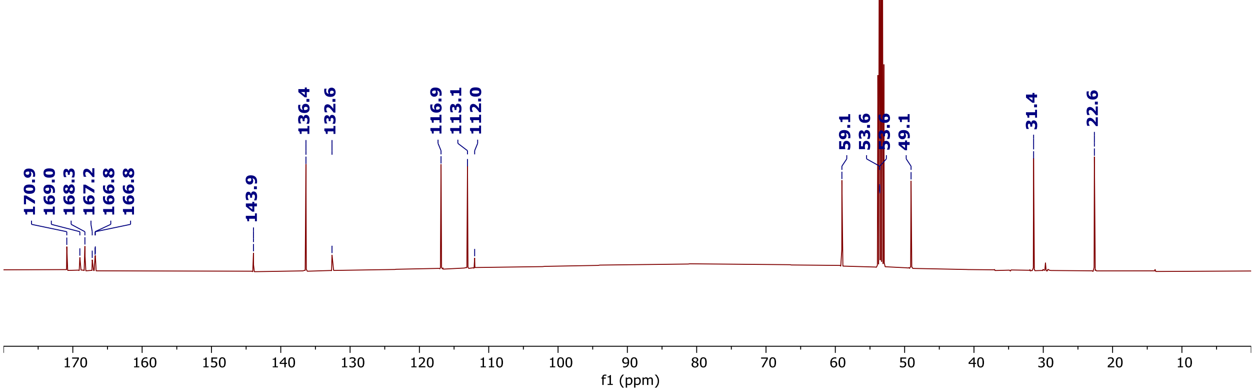

^{1}H NMR (500 MHz, $CDCl_3$)

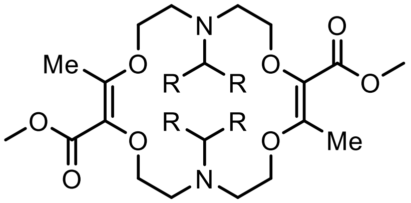

13, R = CO_2Me

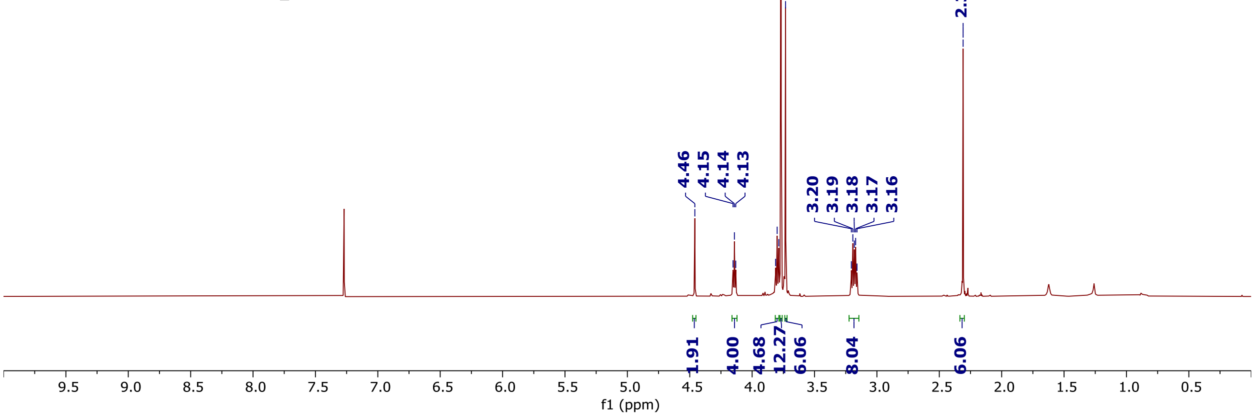

^{13}C NMR (126 MHz, $CDCl_3$)

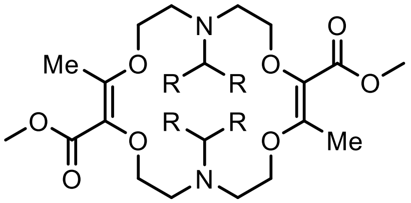

13, R = CO_2Me

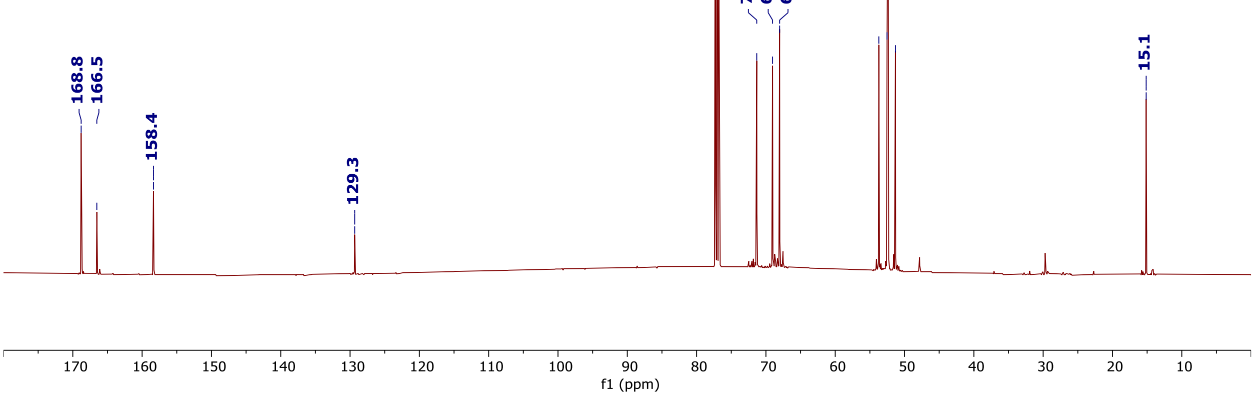

10. References

(1) Biswas, A.; Karmakar, U.; Pal, A.; Samanta, R. Copper-Catalyzed Regioselective Cascade Alkylation and Cyclocondensation of Quinoline N-Oxides with Diazo Esters: Direct Access to Conjugated π-Systems. *Chem. Eur. J.* **2016**, *22*, 13826-13830.

(2) Wang, J.; Xie, J.; Cindy Lee, W.-C.; Wang, D.-S.; Zhang, X. P. Radical differentiation of two ester groups in unsymmetrical diazomalonates for highly asymmetric olefin cyclopropanation. *Chem Catal.* **2022**, *2*, 330-344.

(3) Das, D.; Sahoo, G.; Biswas, A.; Samanta, R. RhIII-Catalyzed Synthesis of Highly Substituted 2-Pyridones using Fluorinated Diazomalonate. *Chem. Asian J.* **2020**, *15*, 360-364.

(4) Chow, S.; Green, A. I.; Arter, C.; Liver, S.; Leggott, A.; Trask, L.; Karageorgis, G.; Warriner, S.; Nelson, A. Efficient Approaches for the Synthesis of Diverse α-Diazo Amides. *Synthesis* **2020**, *52*, 1695-1706.

(5) Xu, P.; Daniliuc, C. G.; Bergander, K.; Stein, C.; Studer, A. Synthesis of Five-Membered Ring Systems Bearing gem-Difluoroalkenyl and Monofluoroalkenyl Substituents via Radical β-Bromo Fragmentation. *ACS Catal.* **2022**, *12*, 11934-11941.

(6) Dénès, F.; Beaufils, F.; Renaud, P. Thiophenol-Mediated 1,5-Hydrogen Transfer for the Preparation of Pyrrolizidines, Indolizidines, and Related Compounds. *Org. Lett.* **2007**, *9*, 4375-4378.

(7) Rueping, M.; Leonori, D.; Poisson, T. Visible light mediated azomethine ylide formation—photoredox catalyzed [3+2] cycloadditions. *Chem. Commun.* **2011**, *47*, 9615-9617.

(8) Yang, M.; Wang, X.; Li, H.; Livant, P. A New Route To Hindered Tertiary Amines. *J. Org. Chem.* **2001**, *66*, 6729-6733.

(9) Ugarriza, I.; Uria, U.; Carrillo, L.; Vicario, J. L.; Reyes, E. Base-Promoted C→N Acyl Rearrangement: An Unconventional Approach to α-Amino Acid Derivatives. *Chem. Eur. J.* **2014**, *20*, 11650-11654.

(10) Zhang, J.; Jiang, J.; Li, Y.; Zhao, Y.; Wan, X. A New Strategy for the Construction of α-Amino Acid Esters via Decarboxylation. *Org. Lett.* **2013**, *15*, 3222-3225.

(11) Homberg, A.; Poggiali, D.; Vishe, M.; Besnard, C.; Guénée, L.; Lacour, J. One-Step Synthesis of Diaza Macrocycles by Rh(II)-Catalyzed [3 + 6 + 3 + 6] Condensations of Morpholines and α-Diazo-β-ketoesters. *Org. Lett.* **2019**, *21*, 687-691.

(12) Dolomanov, O. V.; Bourhis, L. J.; Gildea, R. J.; Howard, J. A. K.; Puschmann, H. OLEX2: a complete structure solution, refinement and analysis program. *J. Appl. Cryst.* **2009**, *42*, 339-341.

(13) Sheldrick, G. SHELXT - Integrated space-group and crystal-structure determination. *Acta Cryst. A* **2015**, *71*, 3-8.

(14) Sheldrick, G. Crystal structure refinement with SHELXL. *Acta Cryst. C* **2015**, *71*, 3-8.
